# Supplementary figures and images for: Melanocortin 1 receptor regulates cholesterol and bile acid metabolism in the liver
Source: eLife. 2023 Jul 25;12:e84782. doi: 10.7554/eLife.84782 (PMC10368426; doi:10.7554/eLife.84782)

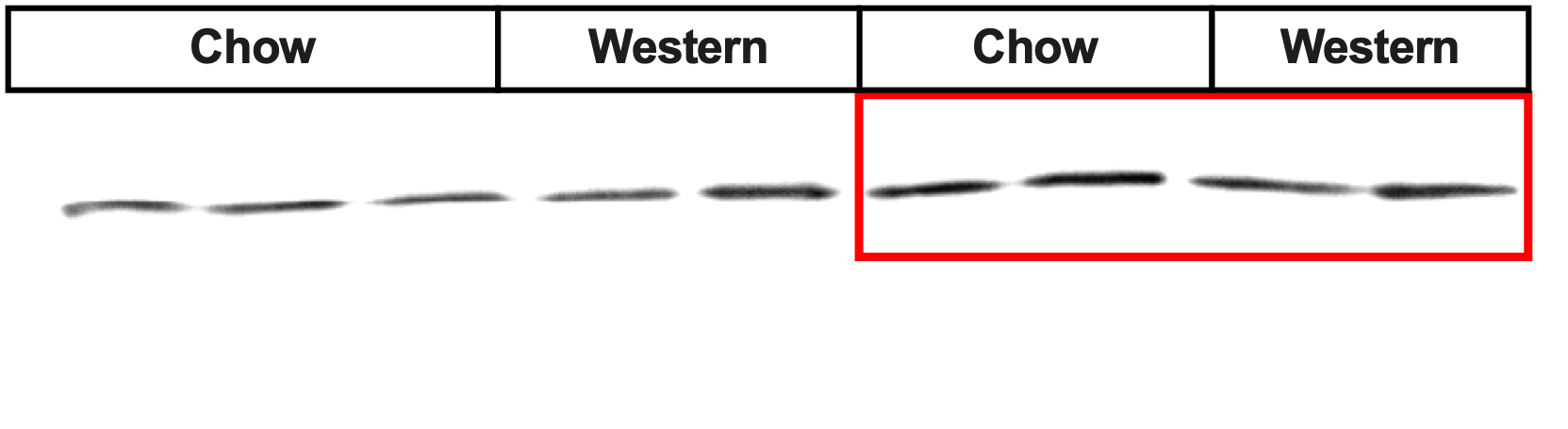

Supplement: Figure 1—source data 1. [file elife-84782-fig1-data1.zip › Figure 1D_vinculin_labeled.tiff]

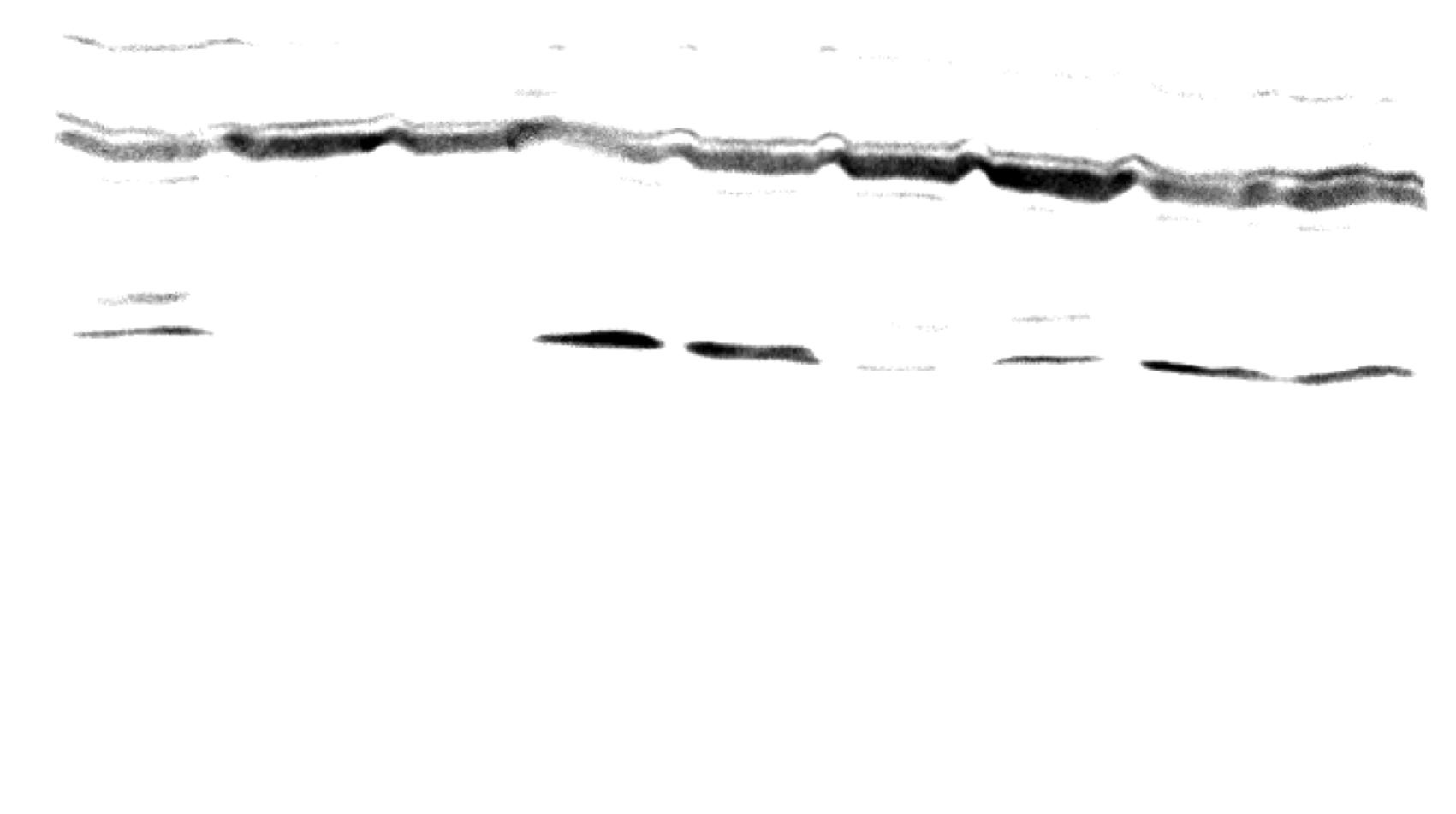

Supplement: Figure 1—source data 1. [file elife-84782-fig1-data1.zip › Figure 1D_MC1-R.tiff]

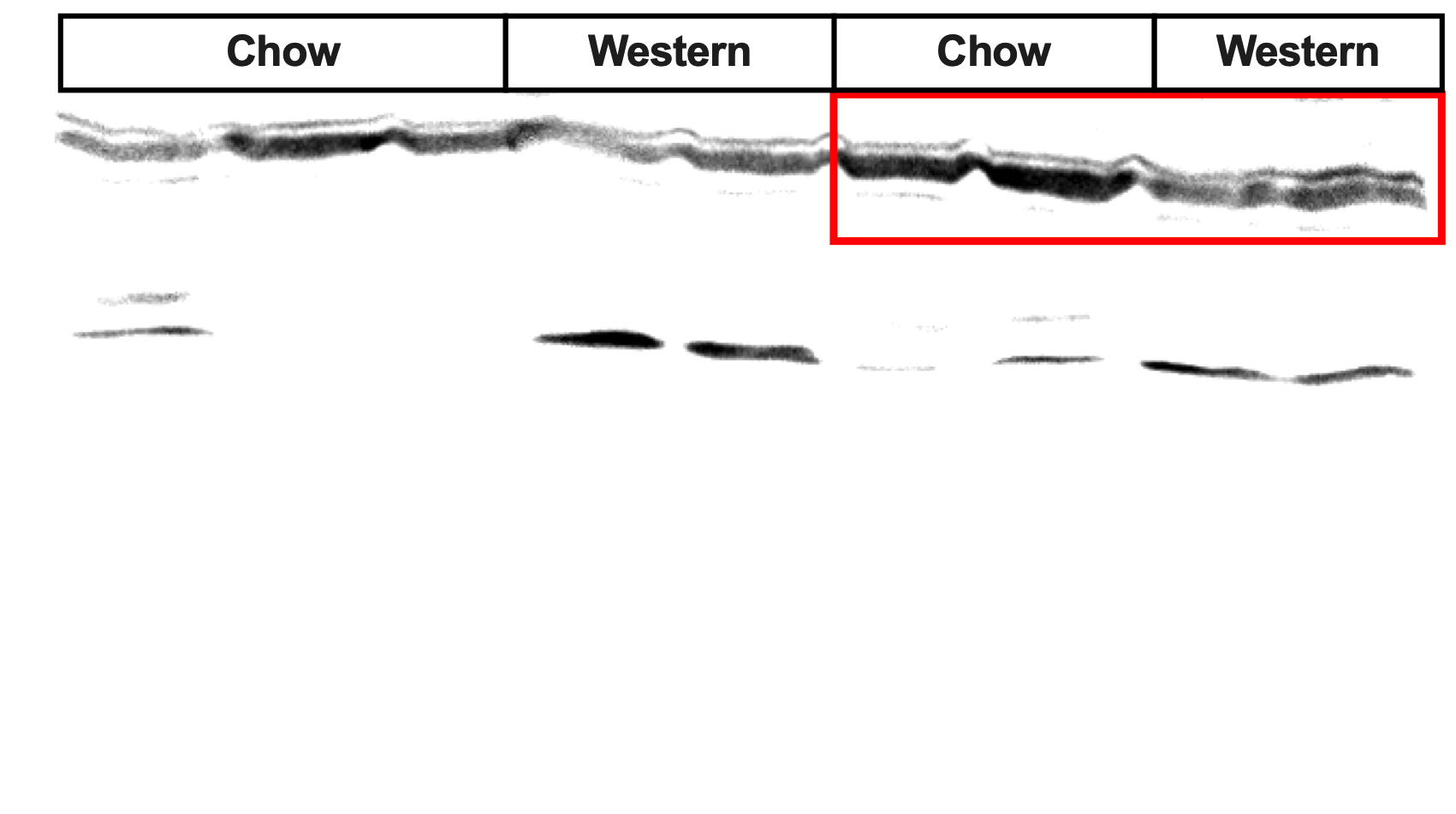

Supplement: Figure 1—source data 1. [file elife-84782-fig1-data1.zip › Figure 1D_MC1-R_labeled.tiff]

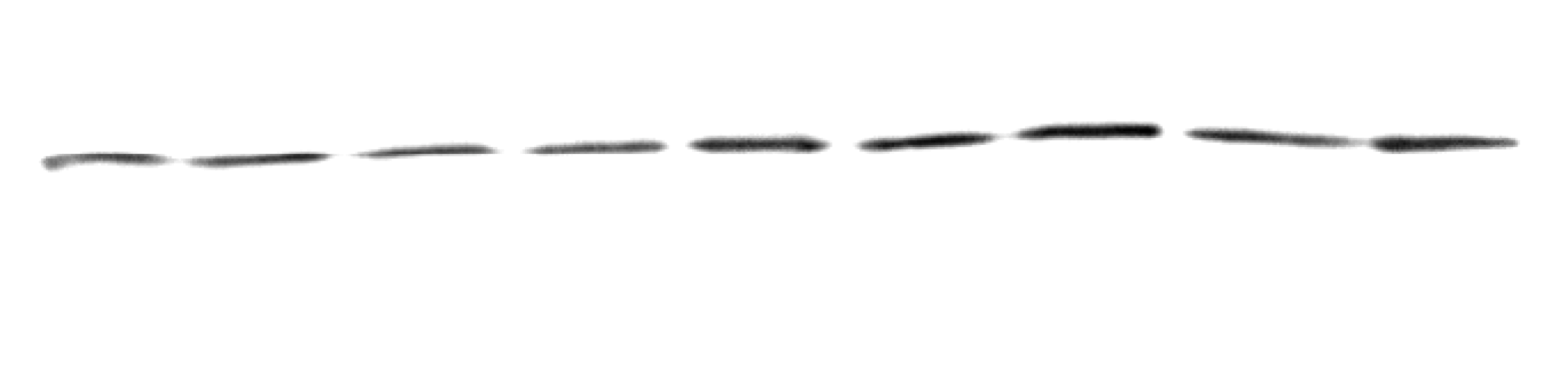

Supplement: Figure 1—source data 1. [file elife-84782-fig1-data1.zip › Figure 1D_vinculin.tiff]

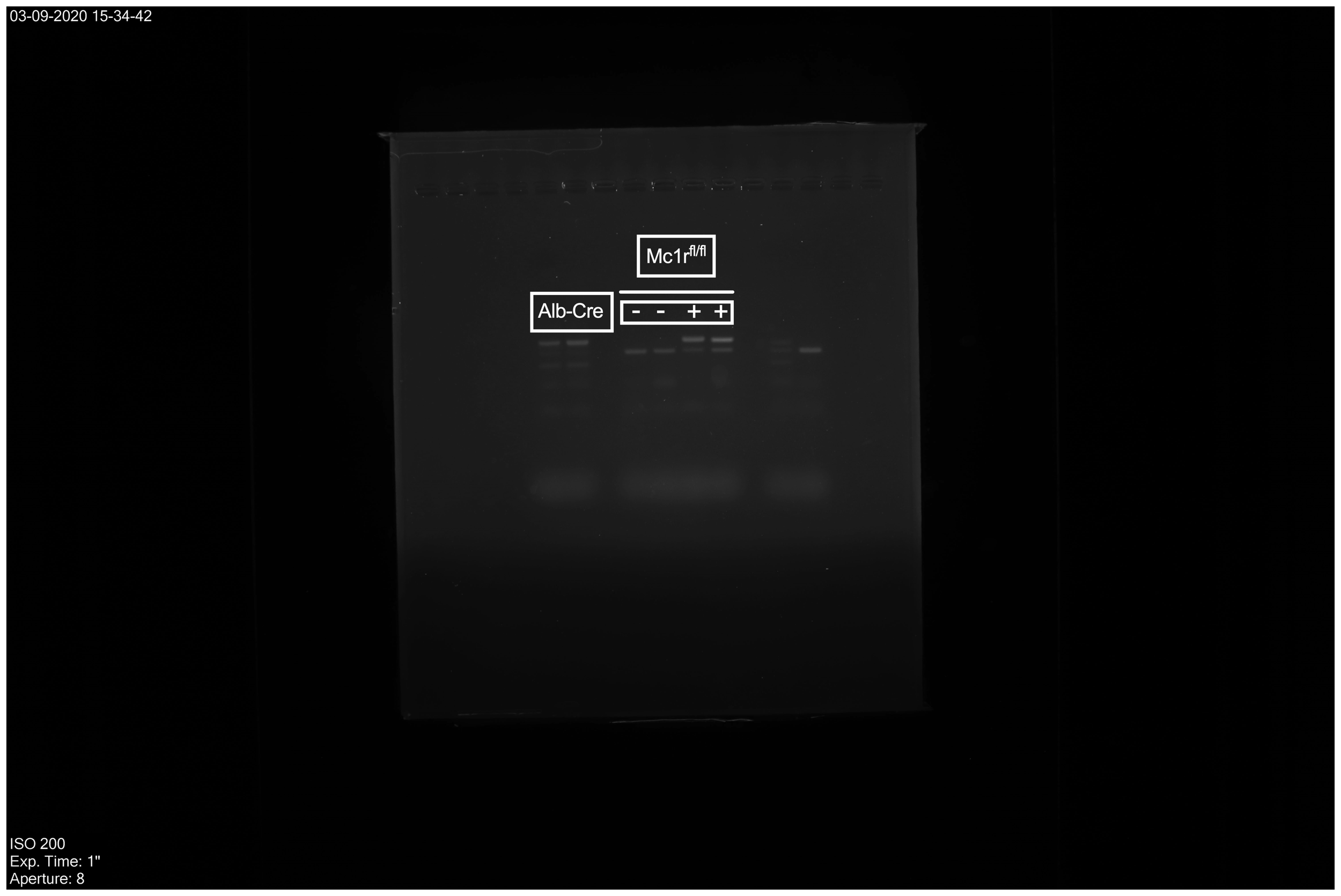

Supplement: Figure 1—source data 1. [file elife-84782-fig1-data1.zip › Figure 1F_labelled.tiff]

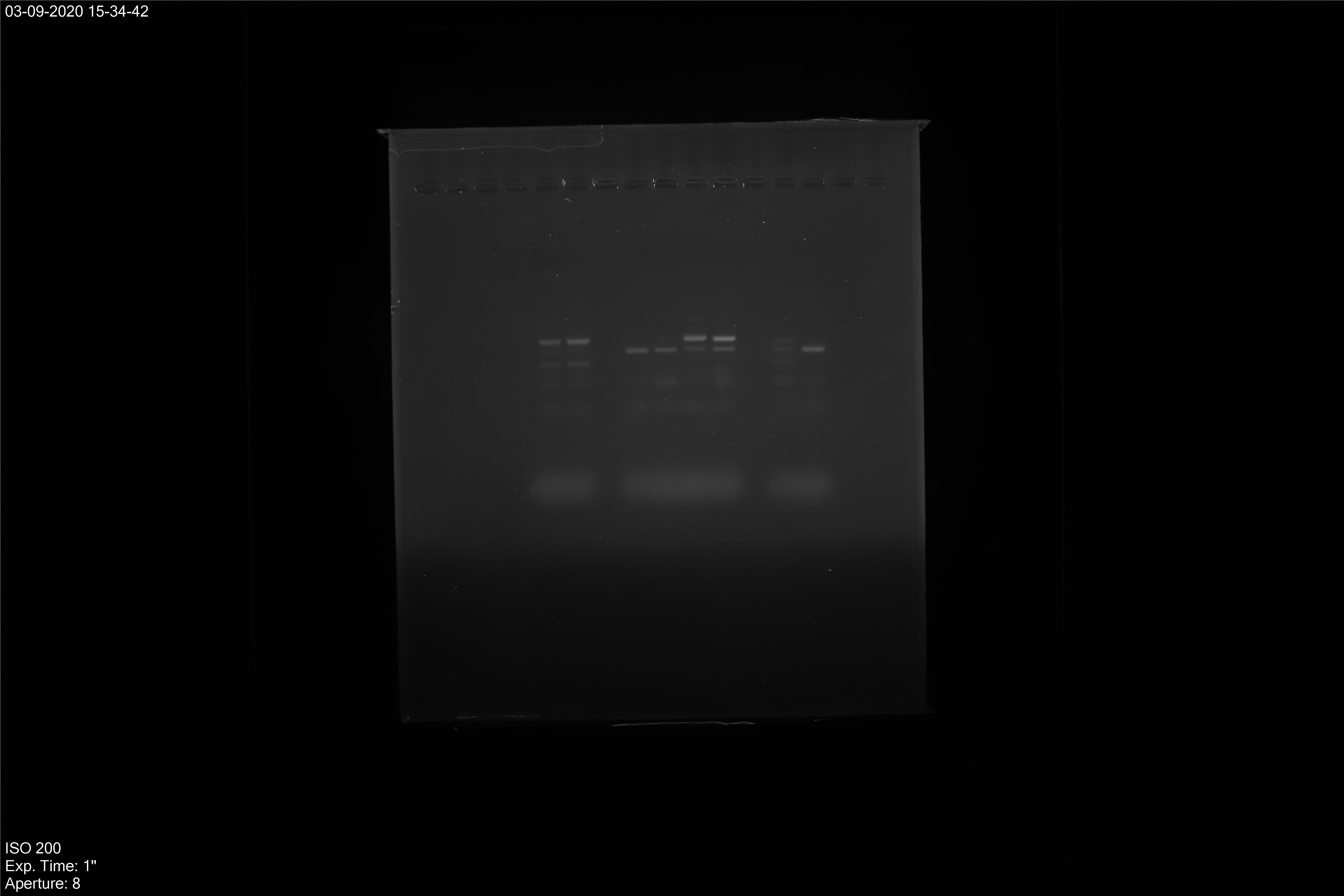

Supplement: Figure 1—source data 1. [file elife-84782-fig1-data1.zip › Figure 1F.tiff]

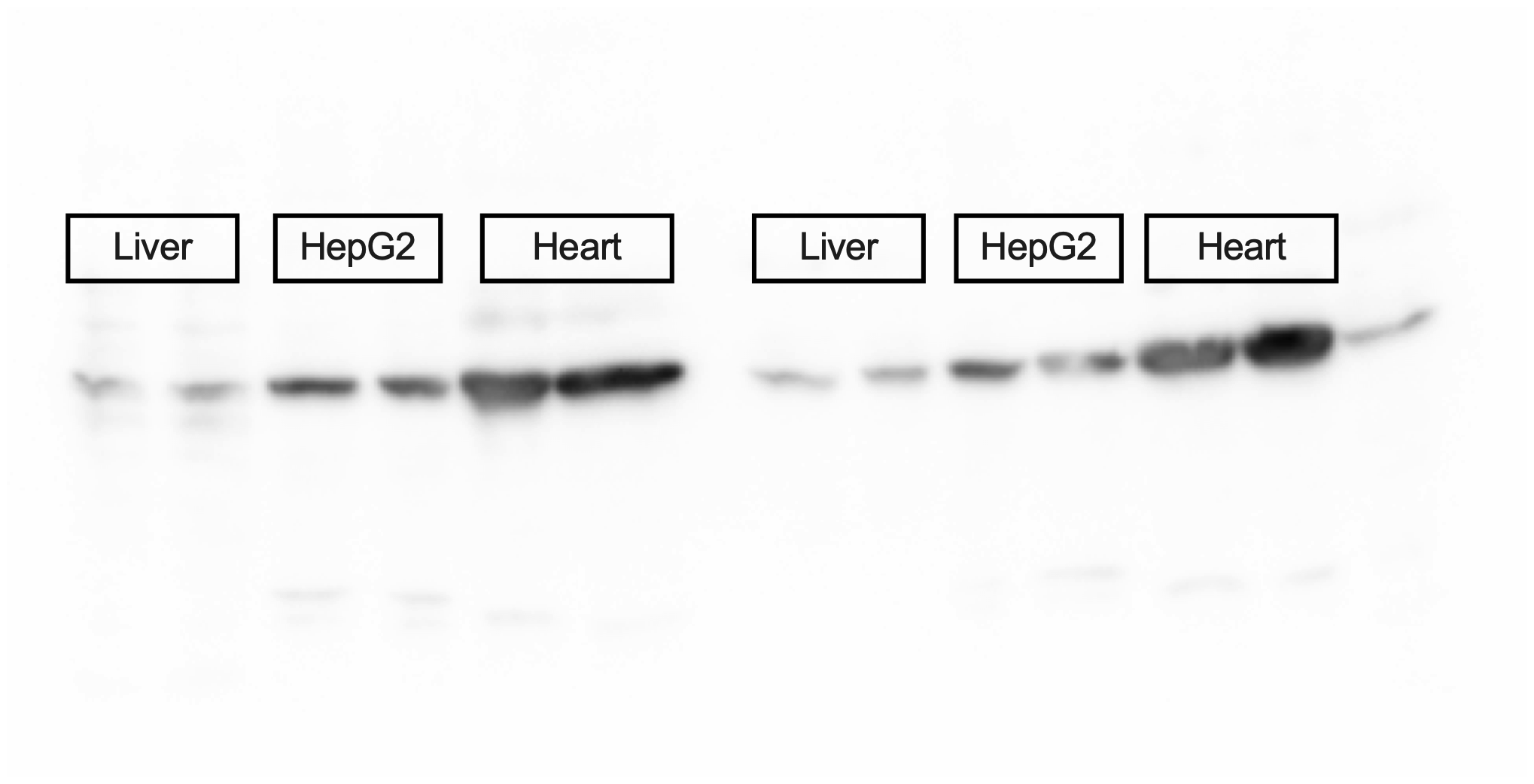

Supplement: Figure 1—figure supplement 2—source data 1. [file elife-84782-fig1-figsupp2-data1.zip › Figure S2_b-actin_labeled.tiff]

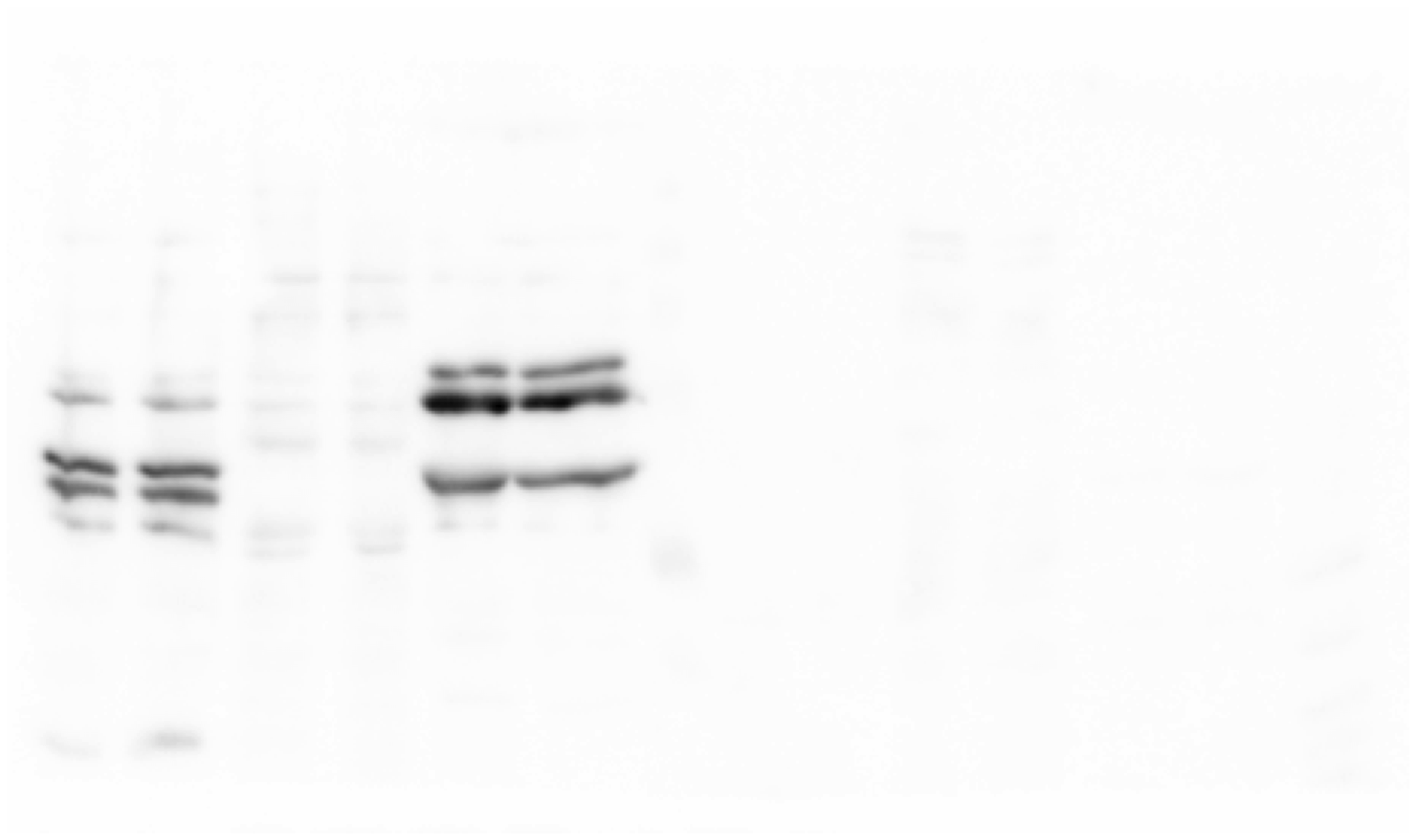

Supplement: Figure 1—figure supplement 2—source data 1. [file elife-84782-fig1-figsupp2-data1.zip › Figure S2_MC1-R.tiff]

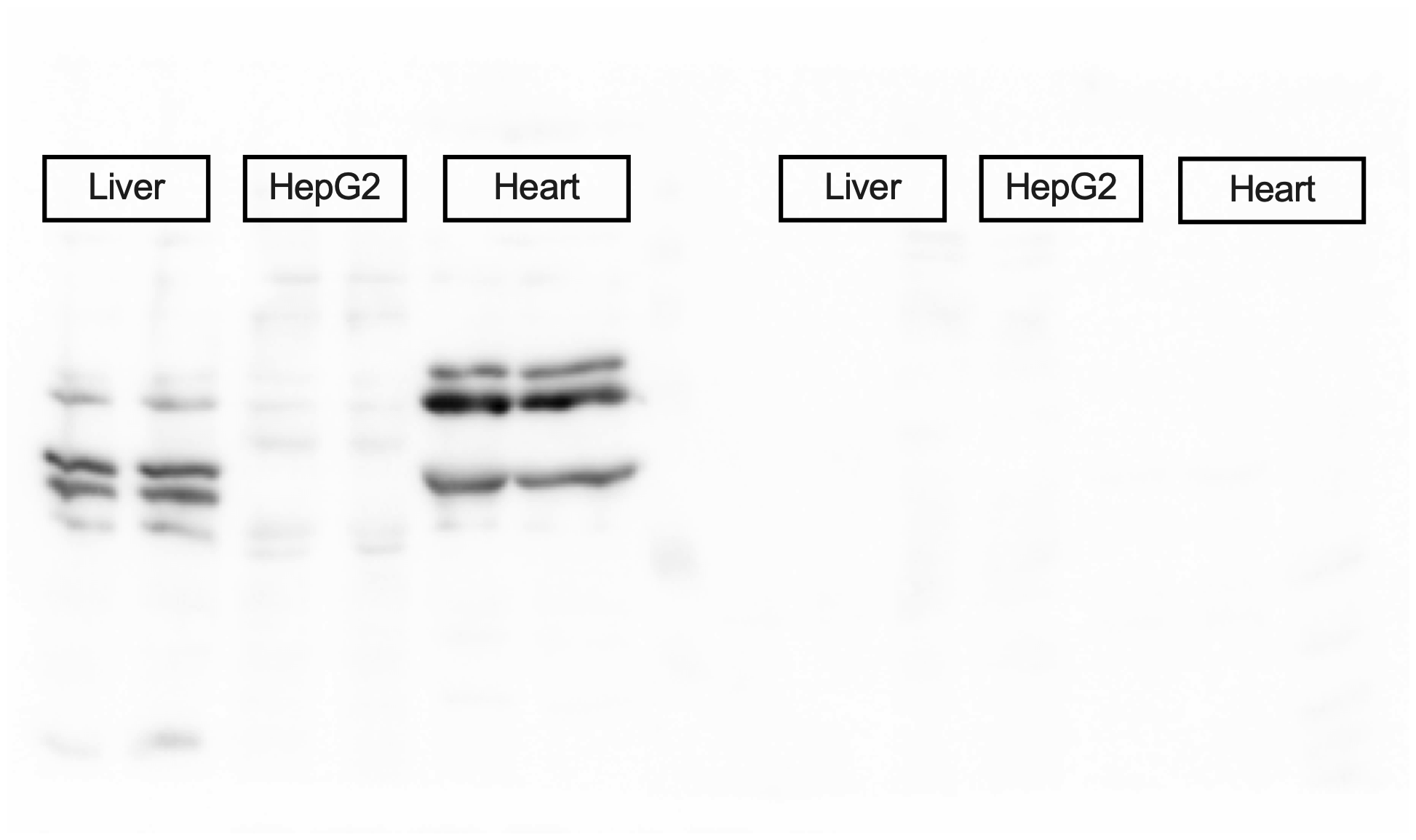

Supplement: Figure 1—figure supplement 2—source data 1. [file elife-84782-fig1-figsupp2-data1.zip › Figure S2_MC1-R_labeled.tiff]

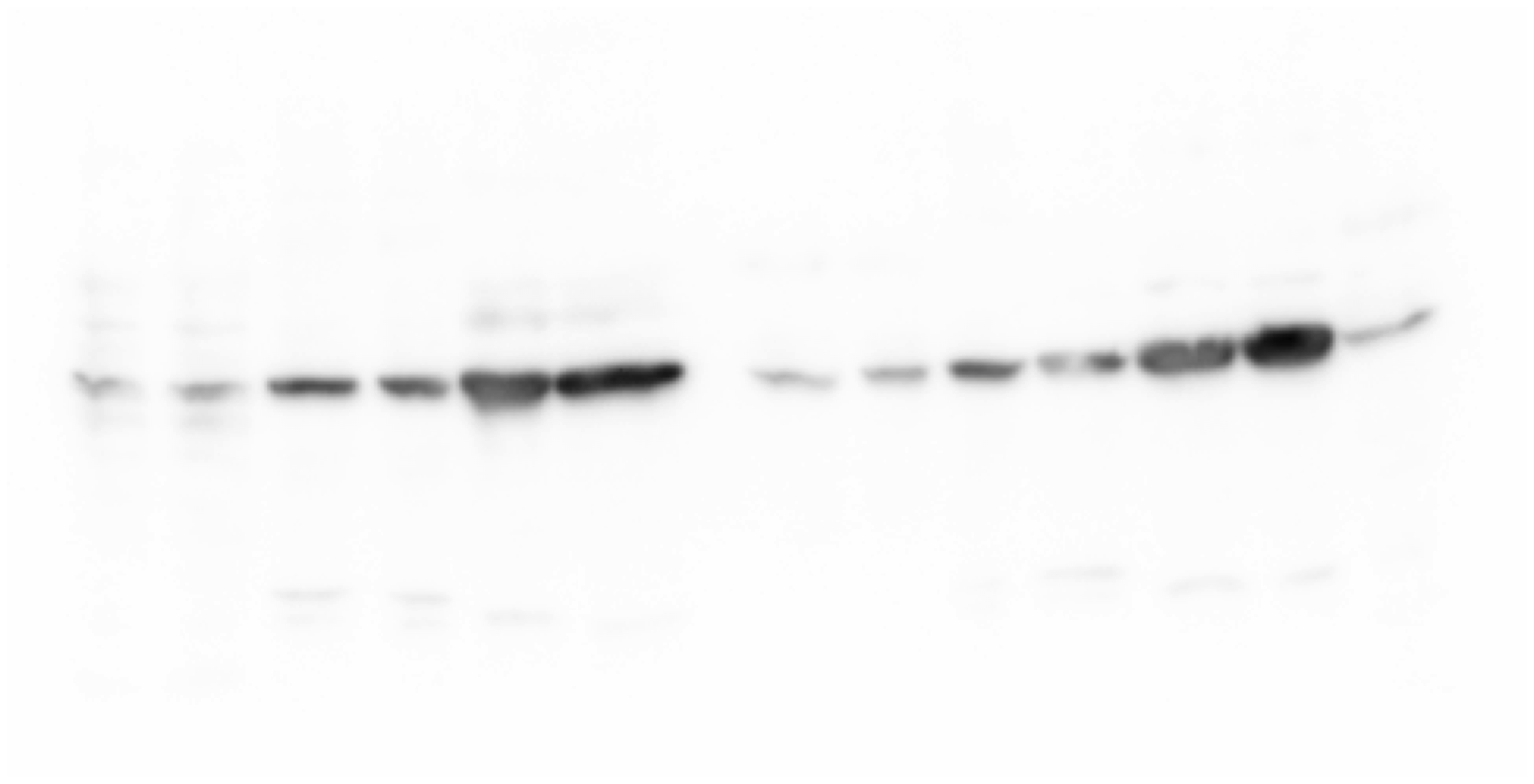

Supplement: Figure 1—figure supplement 2—source data 1. [file elife-84782-fig1-figsupp2-data1.zip › Figure S2_b-actin.tiff]

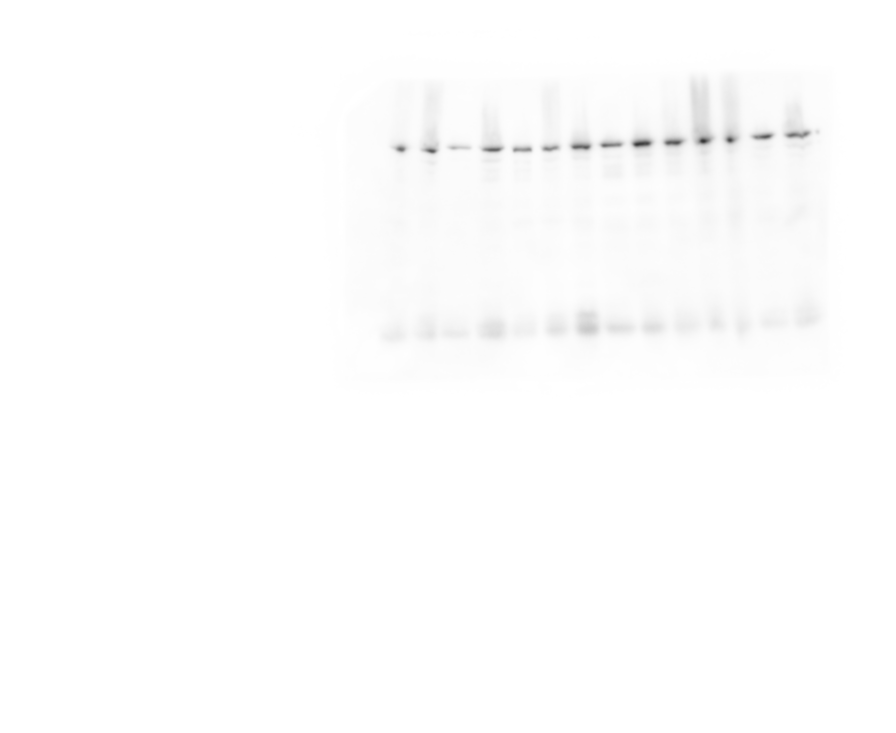

Supplement: Figure 1—figure supplement 3—source data 1. [file elife-84782-fig1-figsupp3-data1.zip › Figure S3A_vinculin.tiff]

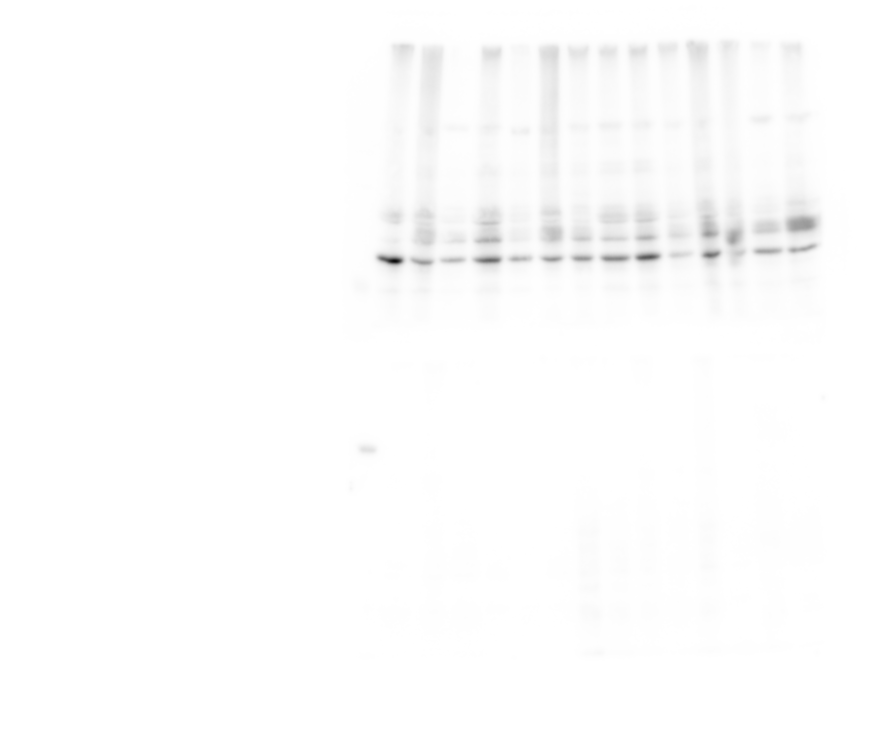

Supplement: Figure 1—figure supplement 3—source data 1. [file elife-84782-fig1-figsupp3-data1.zip › Figure S3A_MC1R.tif]

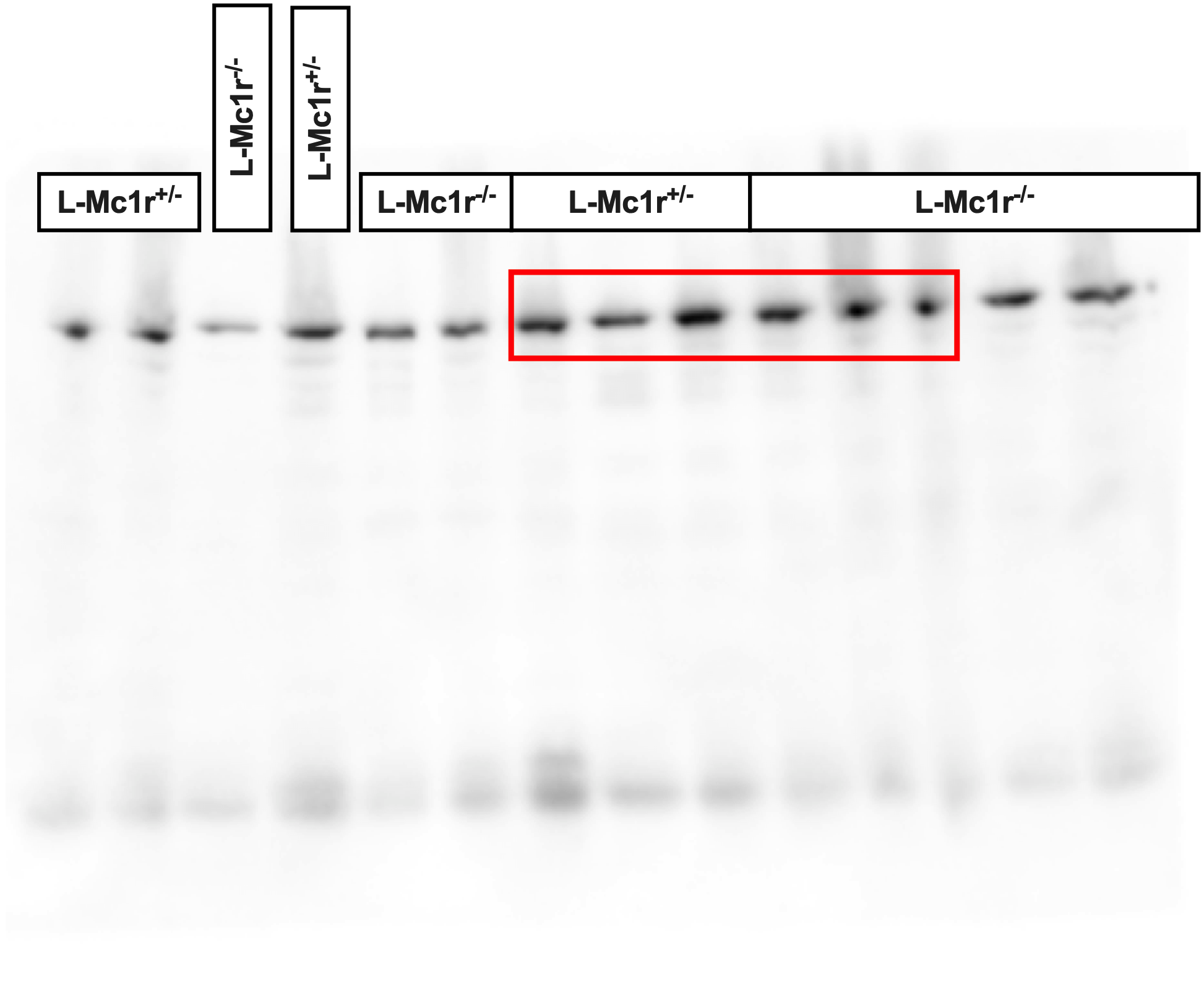

Supplement: Figure 1—figure supplement 3—source data 1. [file elife-84782-fig1-figsupp3-data1.zip › Figure S3A_vinculin_labelled.tiff]

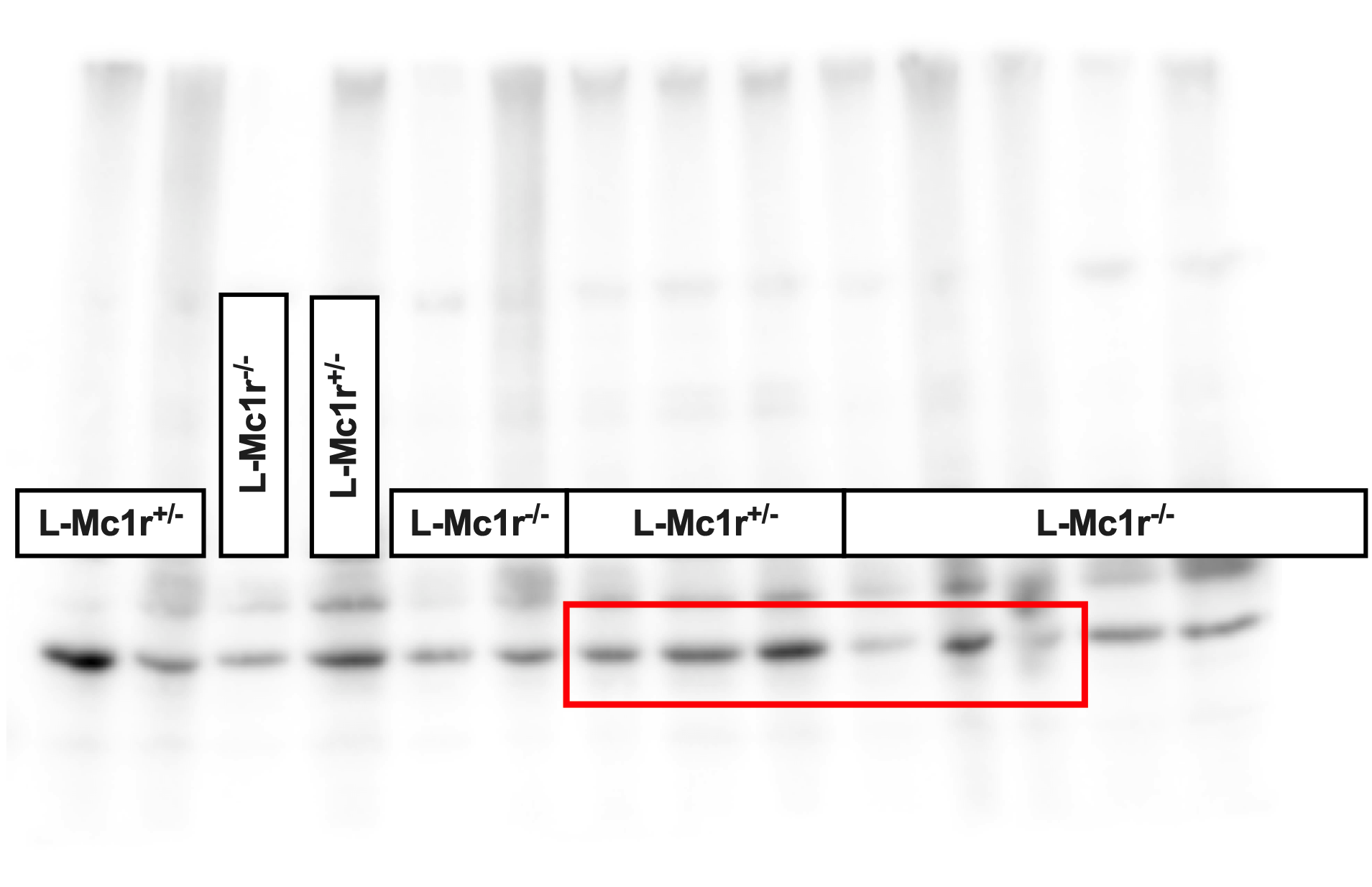

Supplement: Figure 1—figure supplement 3—source data 1. [file elife-84782-fig1-figsupp3-data1.zip › Figure S3A_MC1R_labeled.tiff]

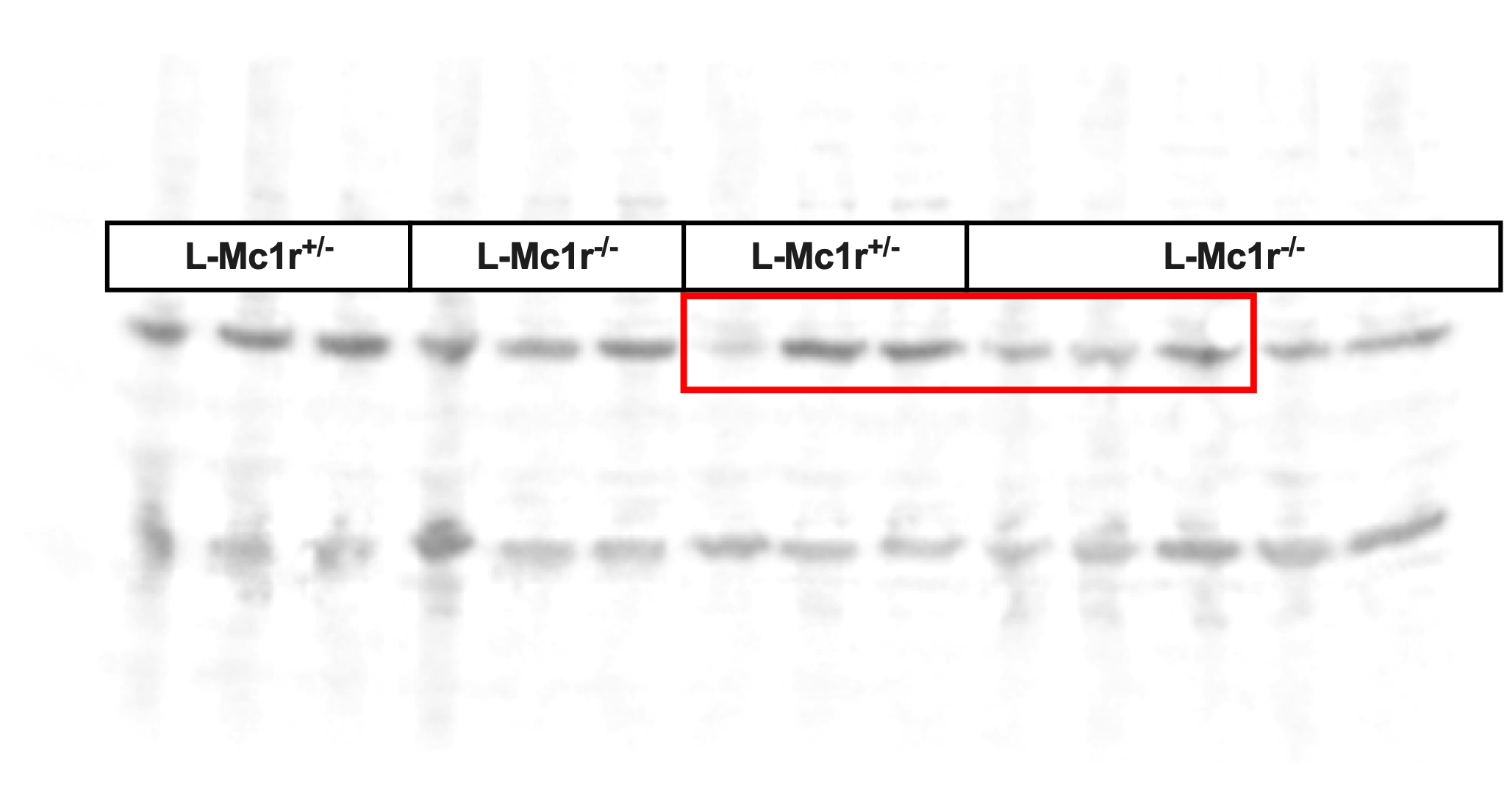

Supplement: Figure 2—source data 1. [file elife-84782-fig2-data1.zip › Figure 2F_DHCR7_labelled.tiff]

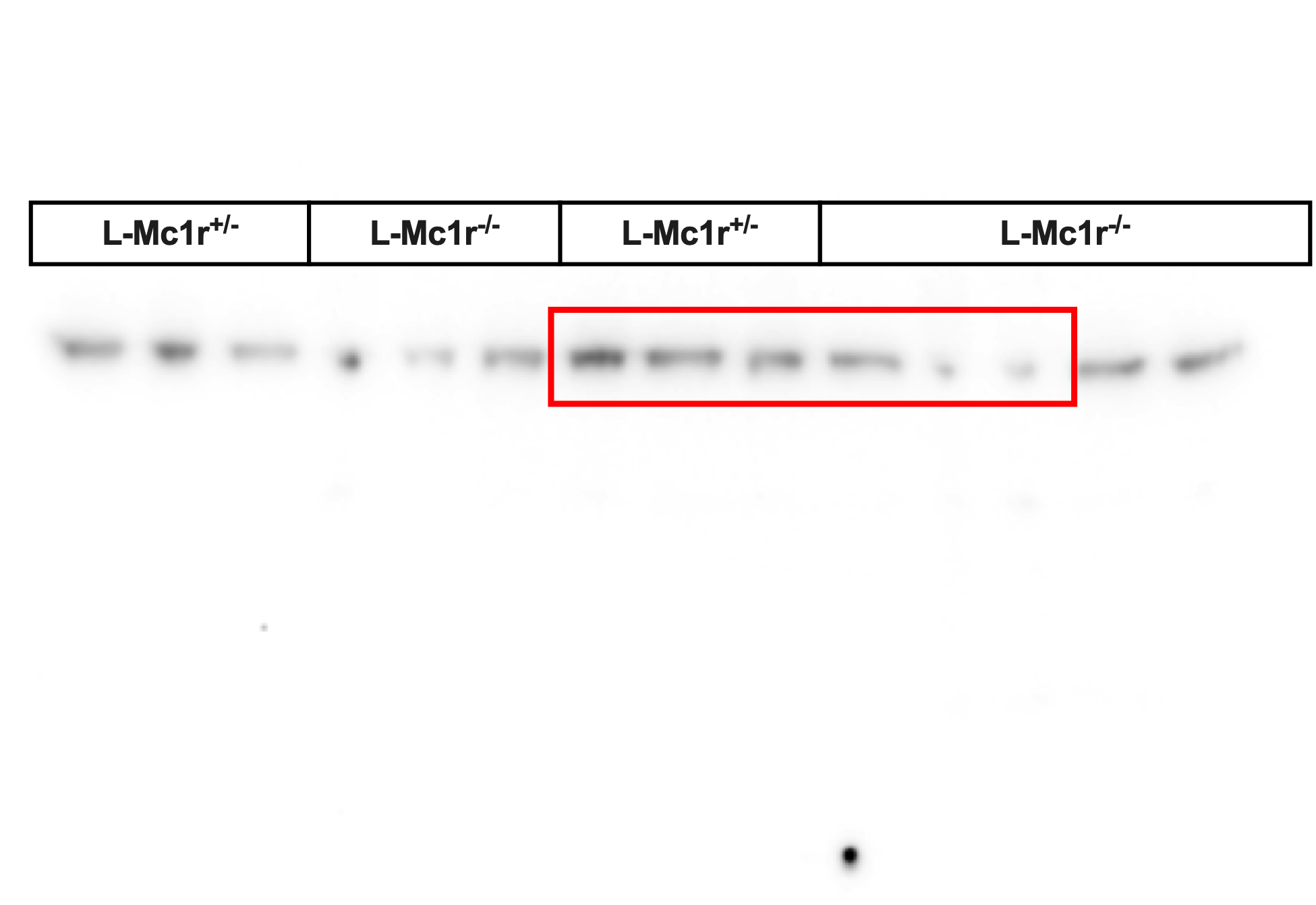

Supplement: Figure 2—source data 1. [file elife-84782-fig2-data1.zip › Figure 2F_HMGCR_labelled.tiff]

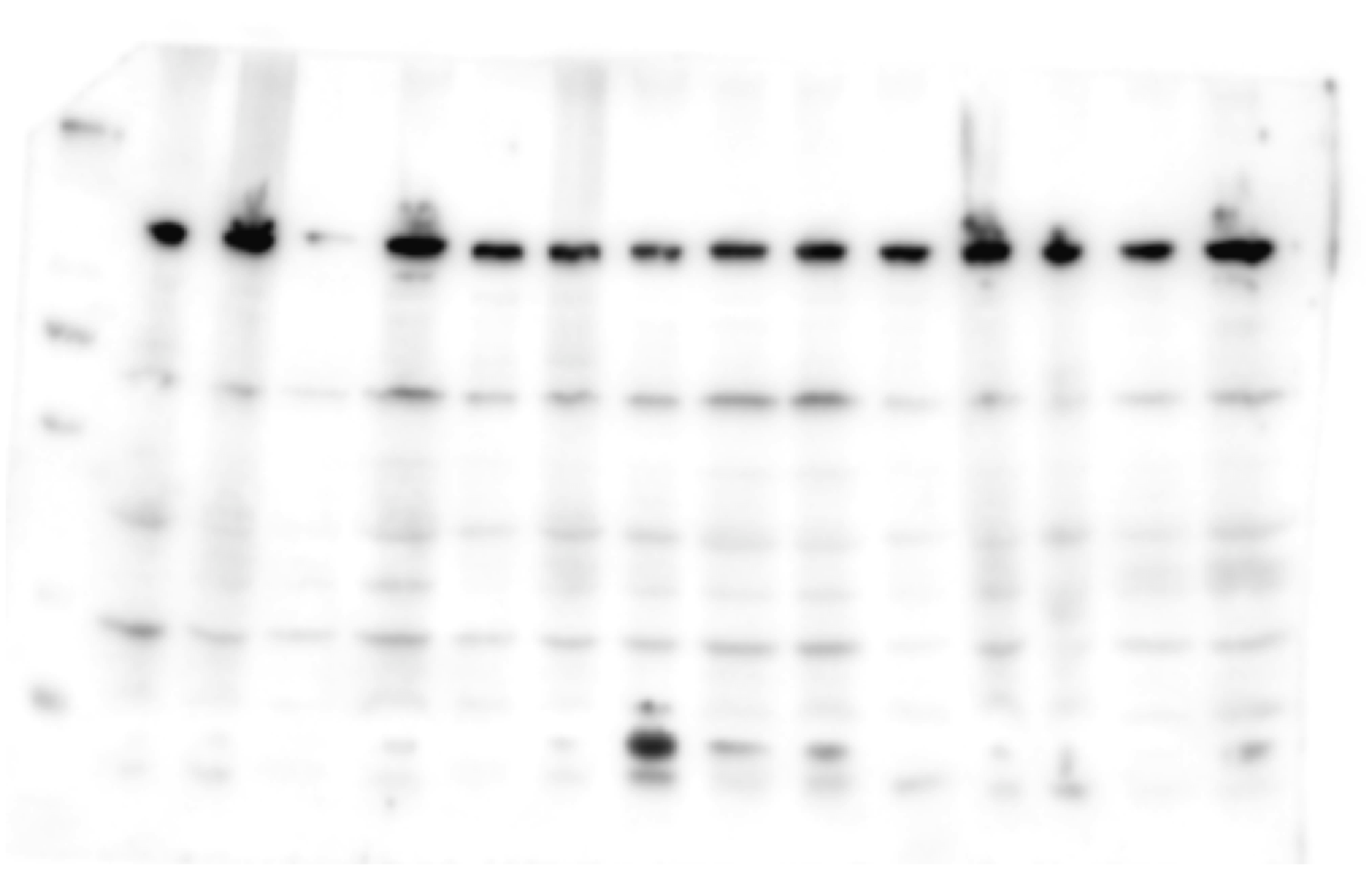

Supplement: Figure 2—source data 1. [file elife-84782-fig2-data1.zip › Figure 2F_SREBP2.tiff]

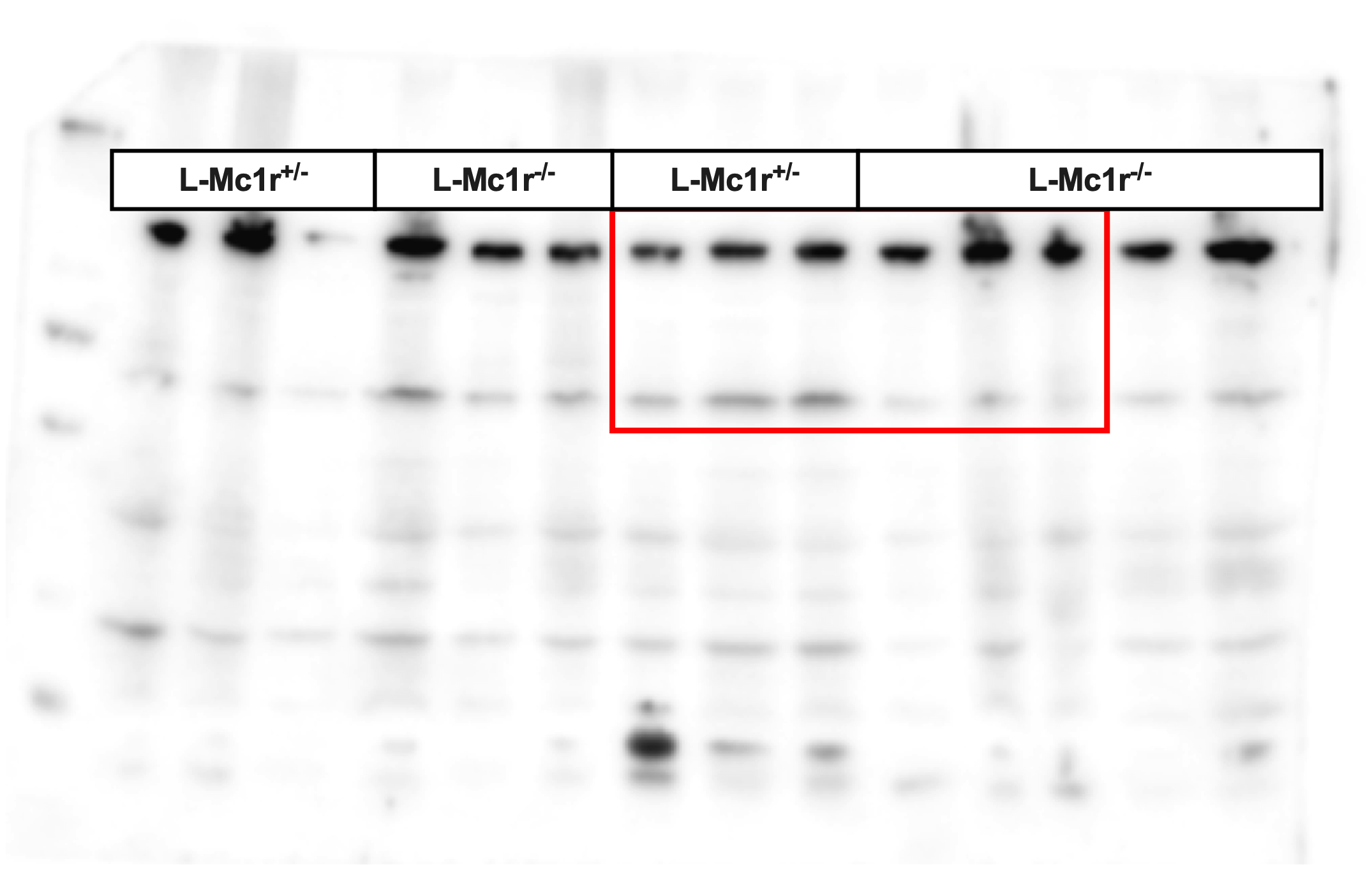

Supplement: Figure 2—source data 1. [file elife-84782-fig2-data1.zip › Figure 2F_SREBP2_labelled.tiff]

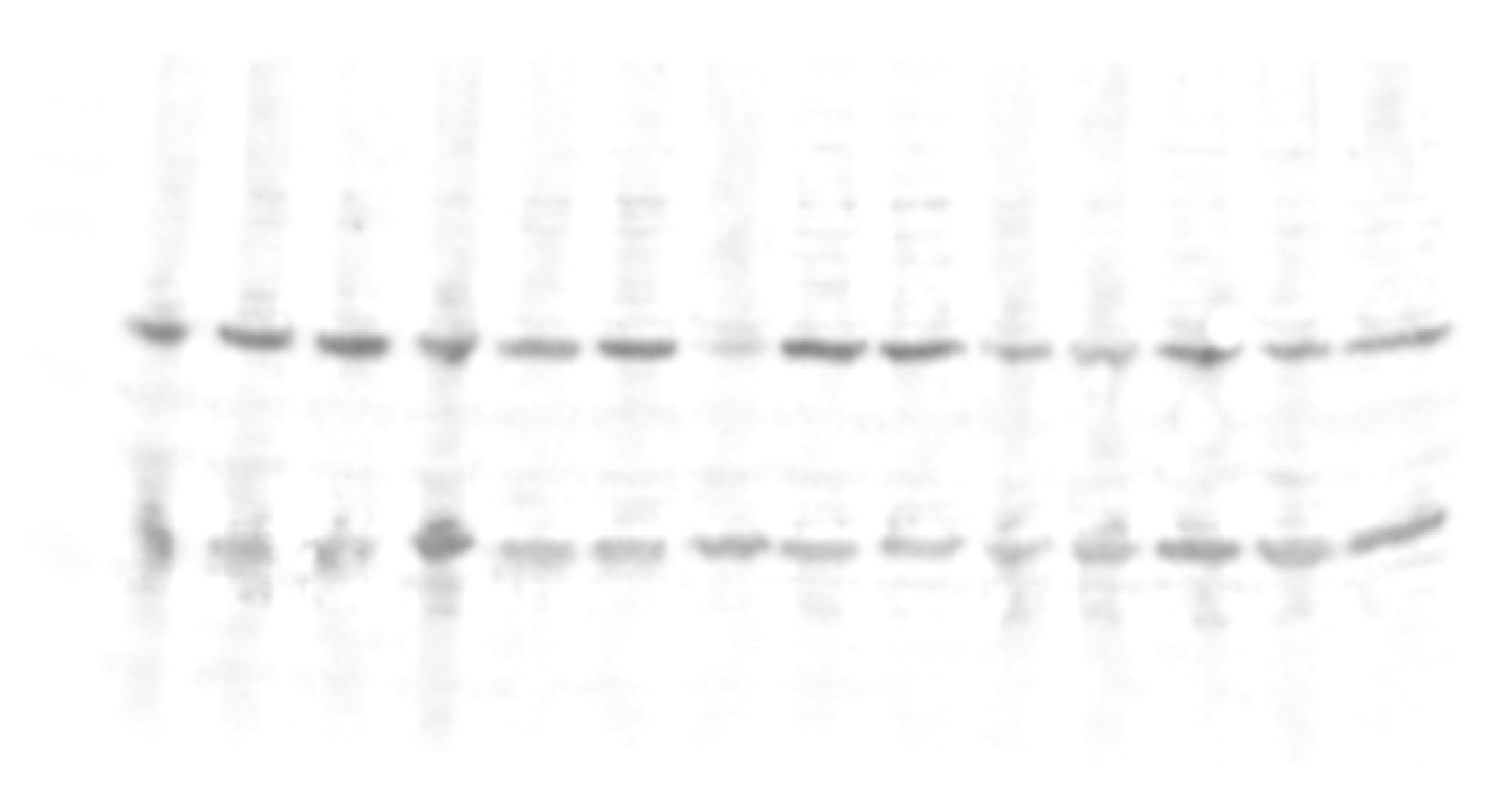

Supplement: Figure 2—source data 1. [file elife-84782-fig2-data1.zip › Figure 2F_DHCR7.tiff]

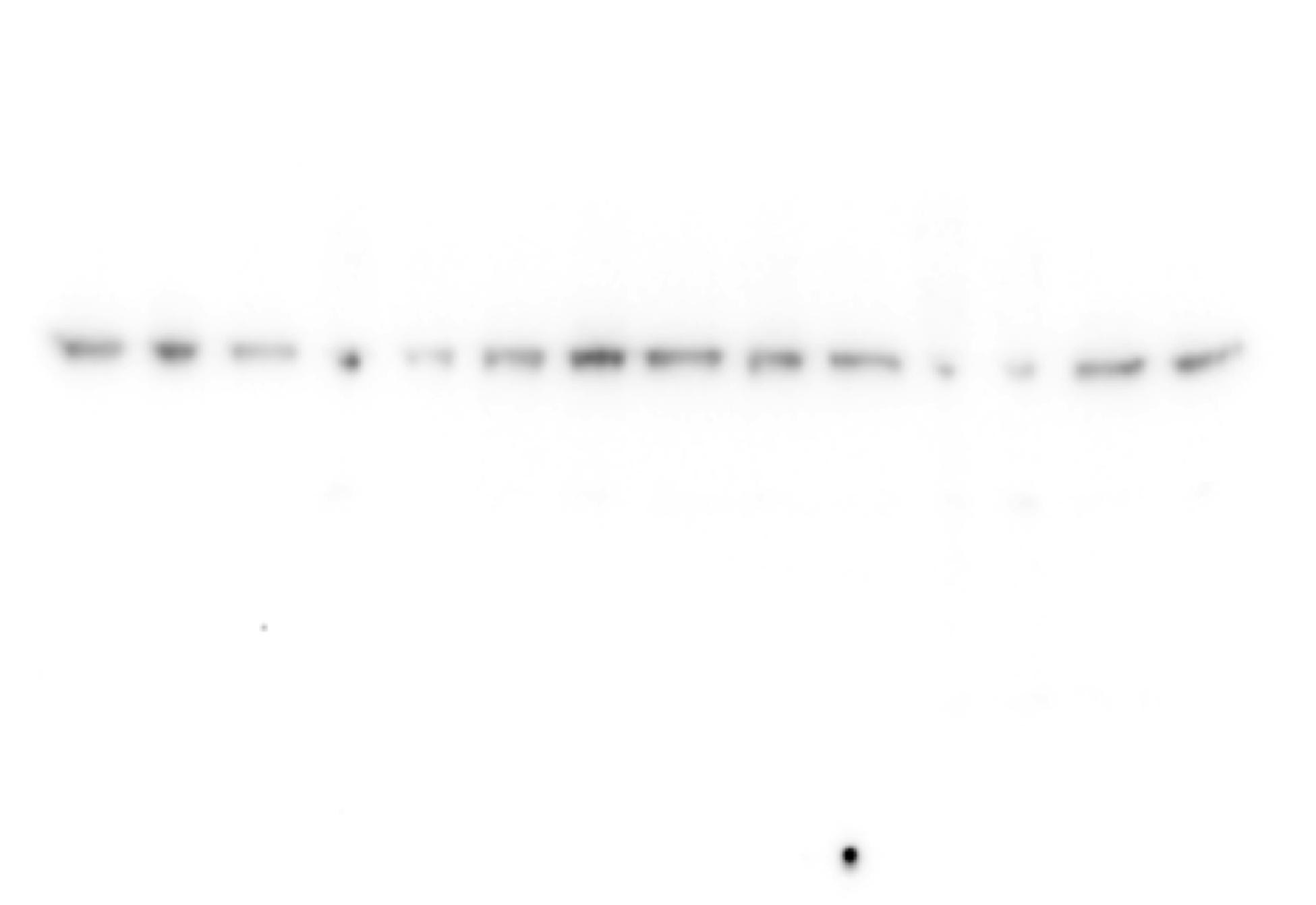

Supplement: Figure 2—source data 1. [file elife-84782-fig2-data1.zip › Figure 2F_HMGCR.tiff]

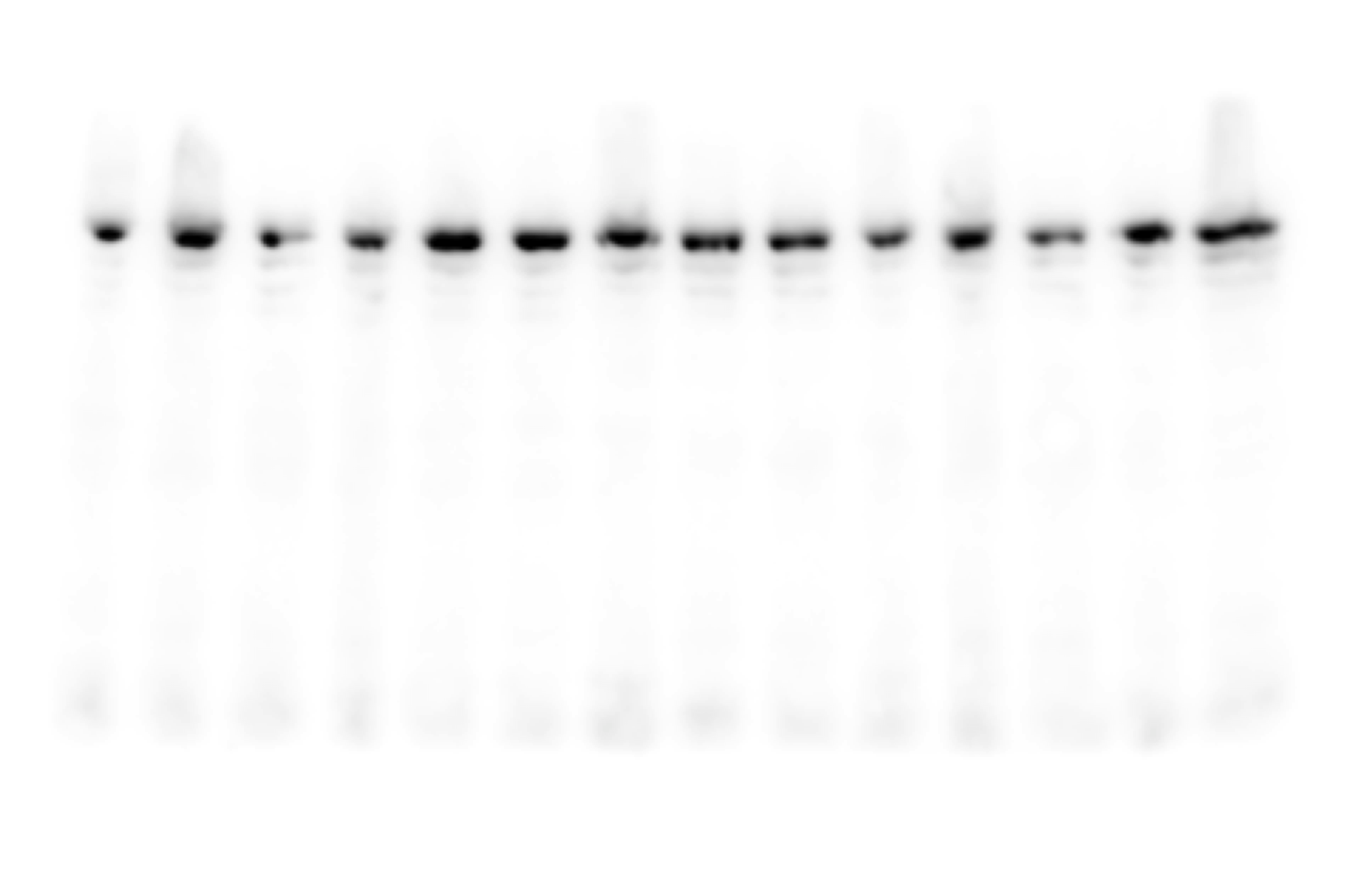

Supplement: Figure 2—source data 1. [file elife-84782-fig2-data1.zip › Figure 2F_vinculin.tiff]

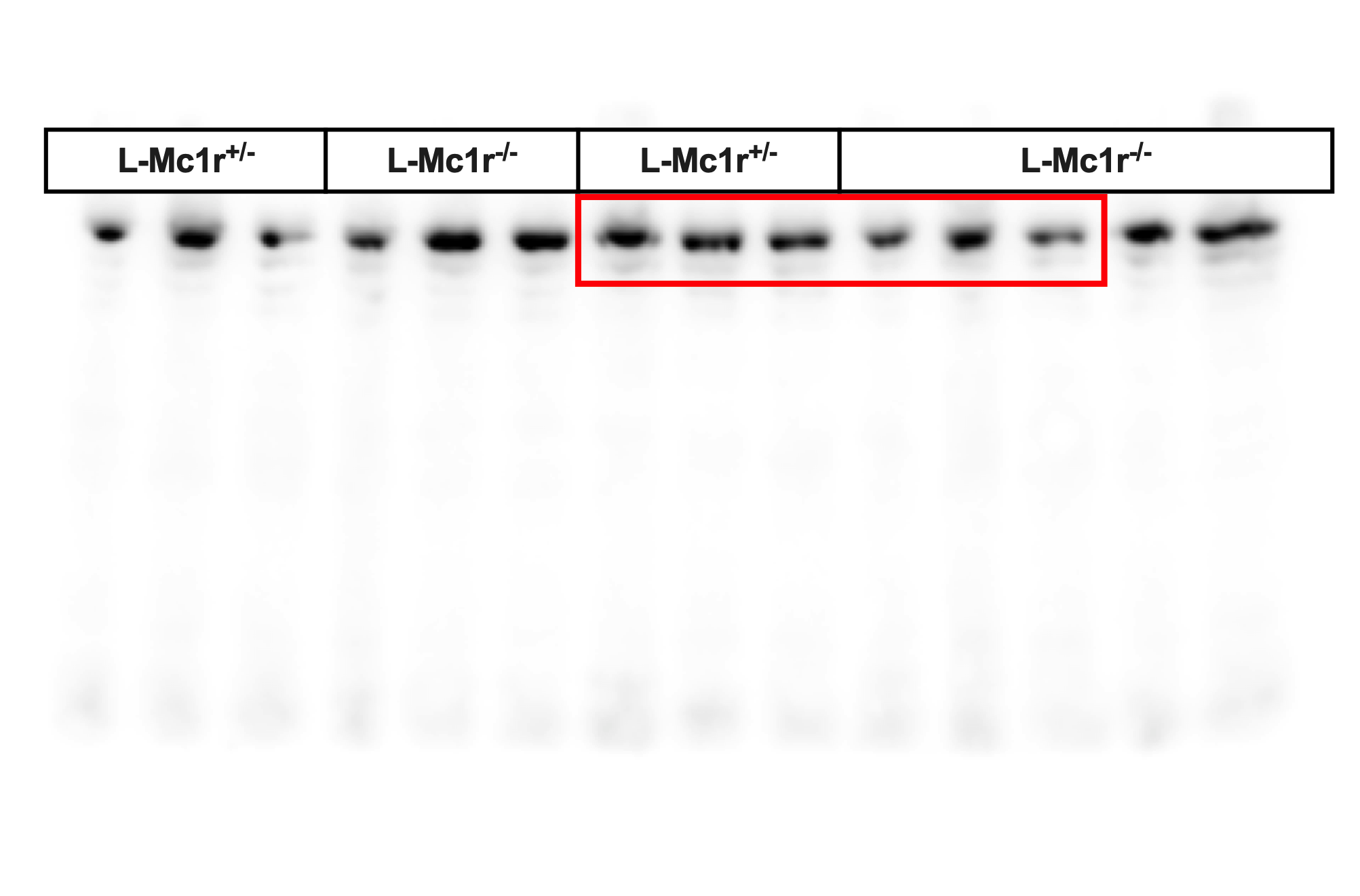

Supplement: Figure 2—source data 1. [file elife-84782-fig2-data1.zip › Figure 2F_vinculin_labelled.tiff]

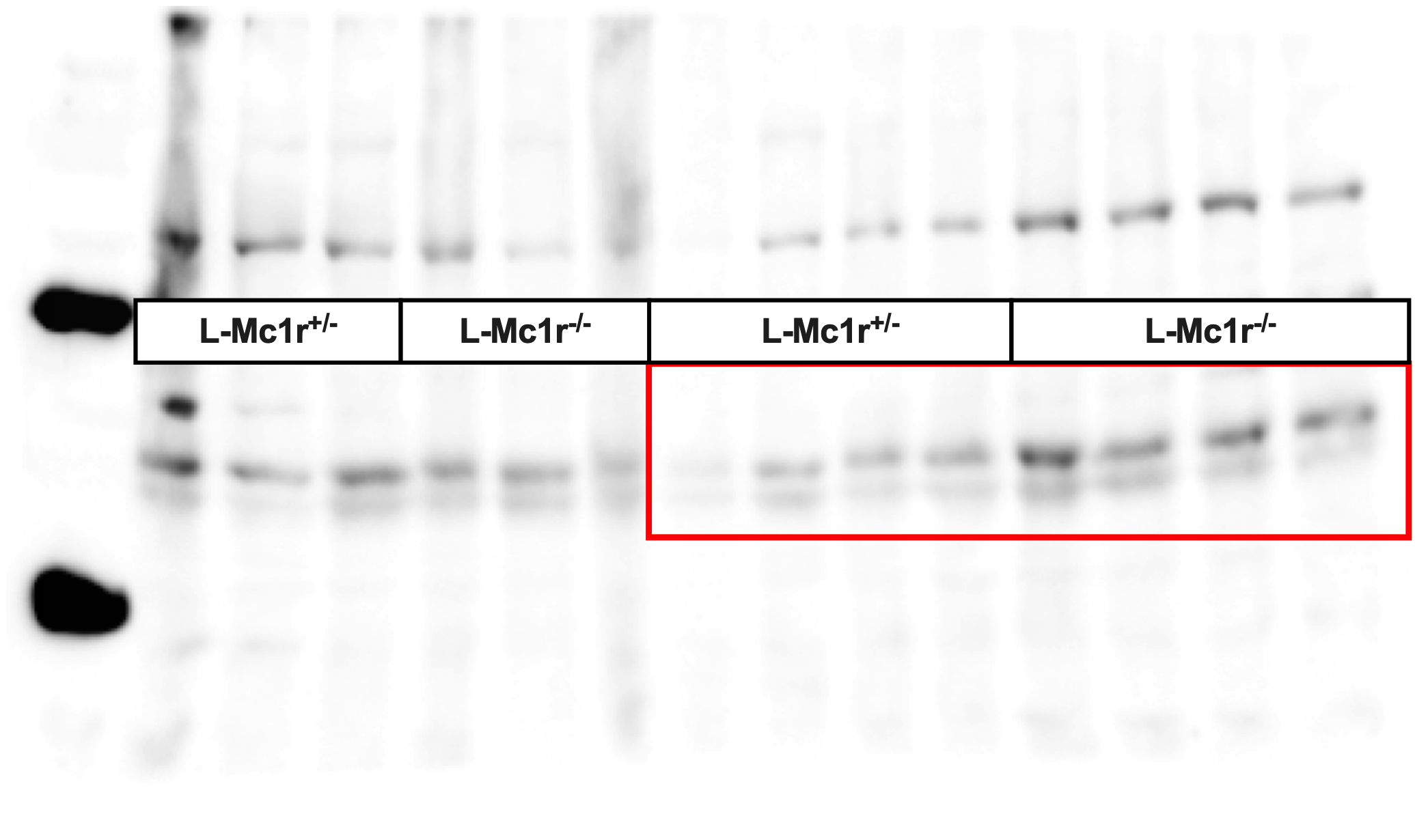

Supplement: Figure 4—source data 1. [file elife-84782-fig4-data1.zip › Fig4_CYP8B1_labeled.tiff]

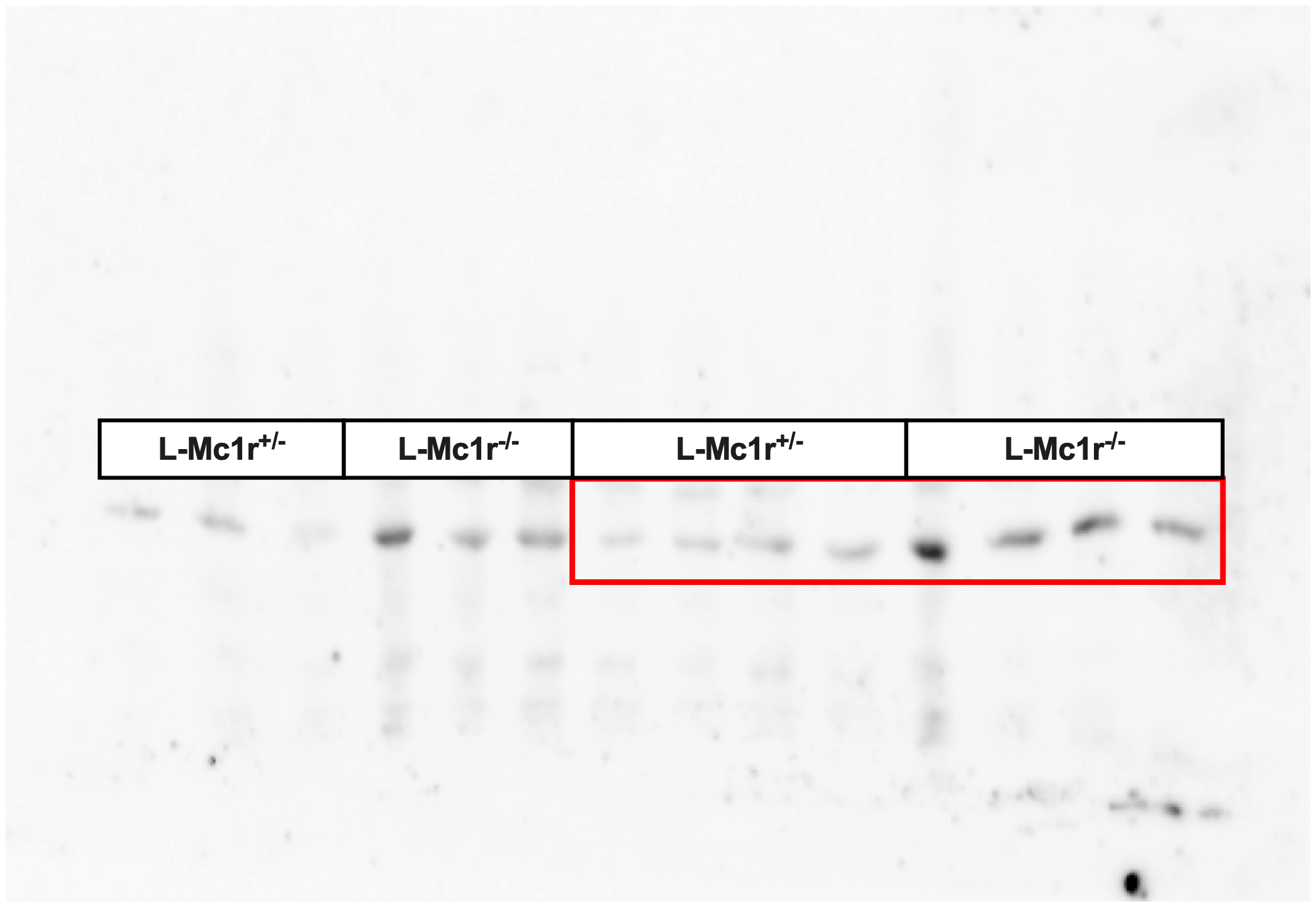

Supplement: Figure 4—source data 1. [file elife-84782-fig4-data1.zip › Fig4_StAR_labeled.tiff]

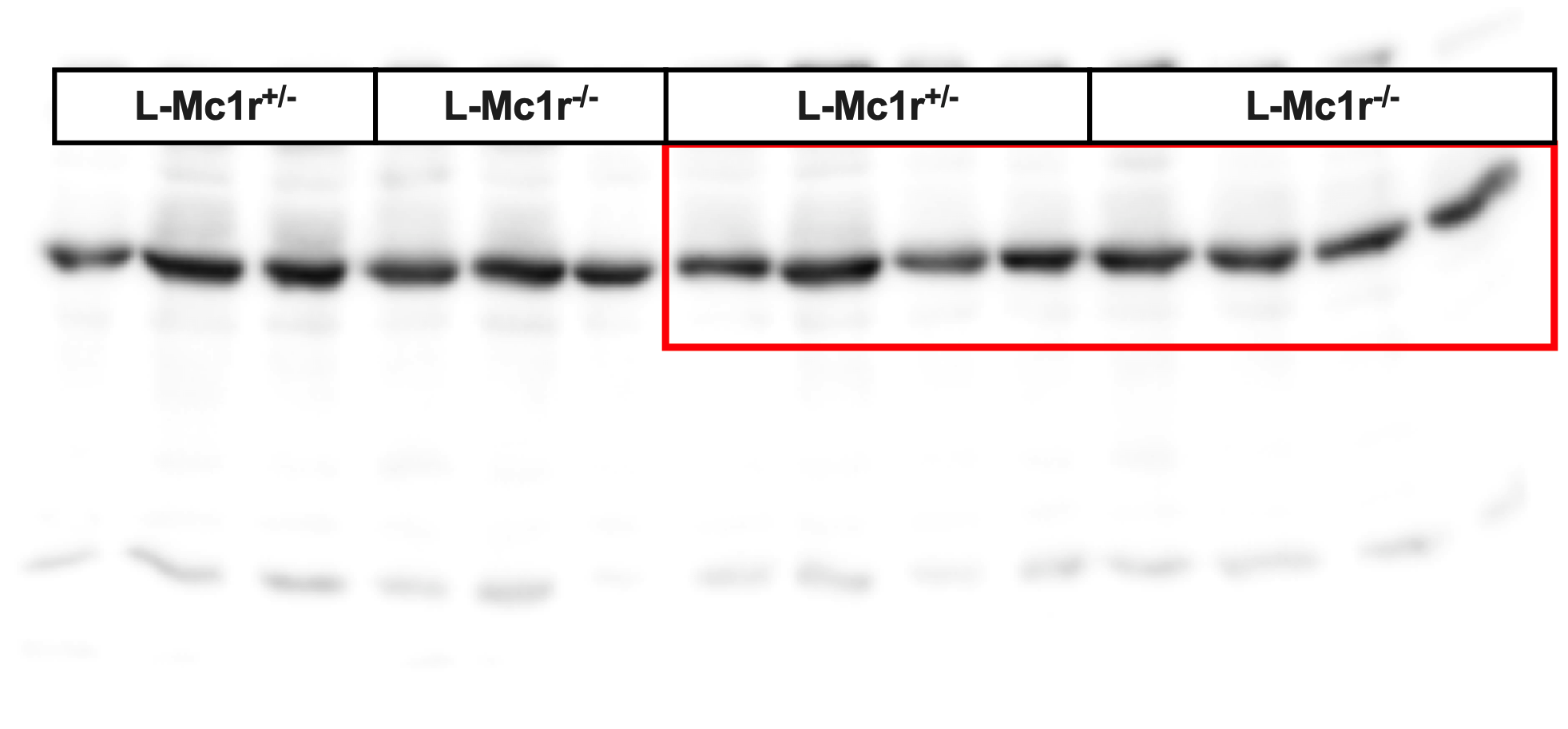

Supplement: Figure 4—source data 1. [file elife-84782-fig4-data1.zip › Fig4_b-actin_labeled.tiff]

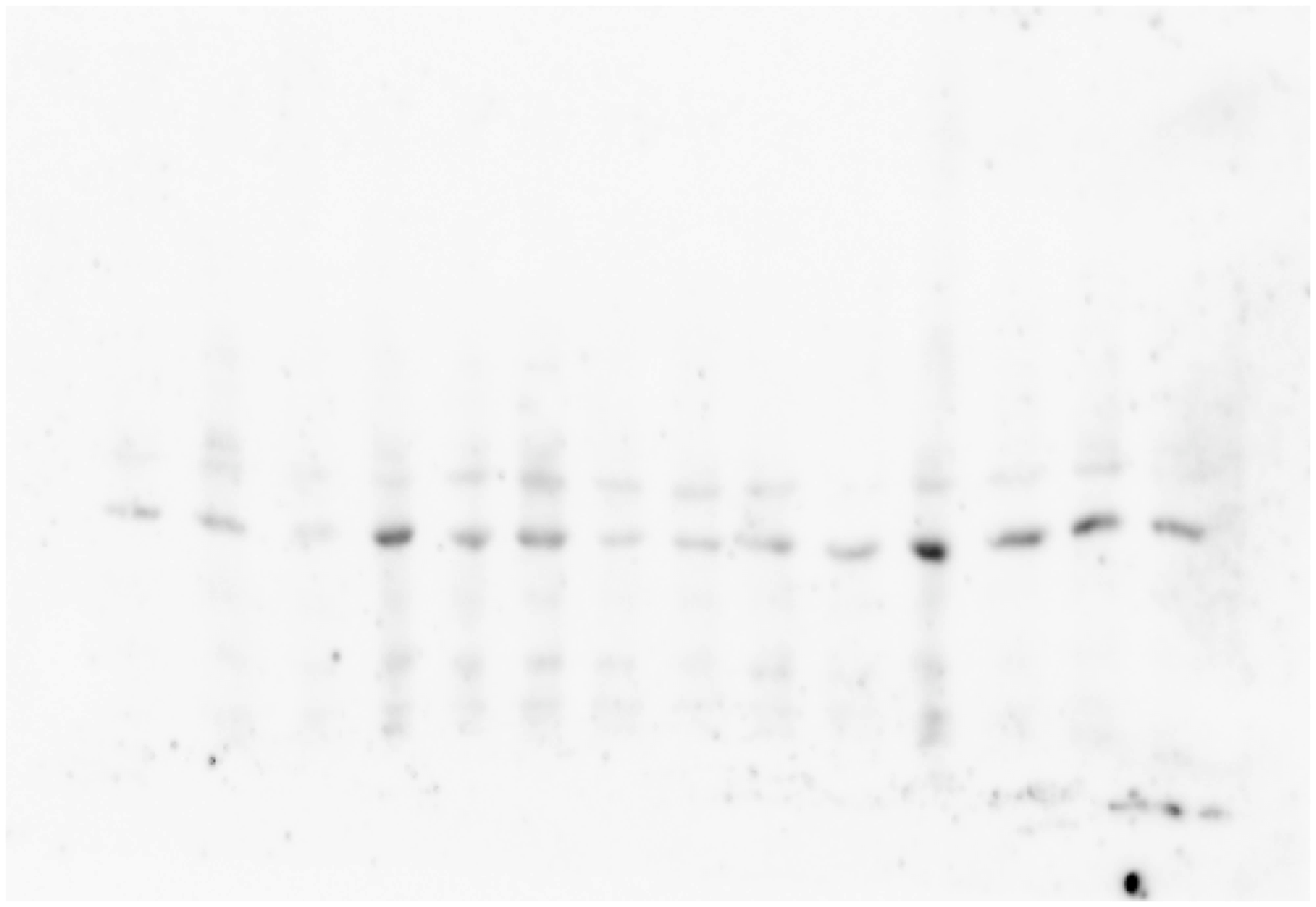

Supplement: Figure 4—source data 1. [file elife-84782-fig4-data1.zip › Fig4_StAR.tiff]

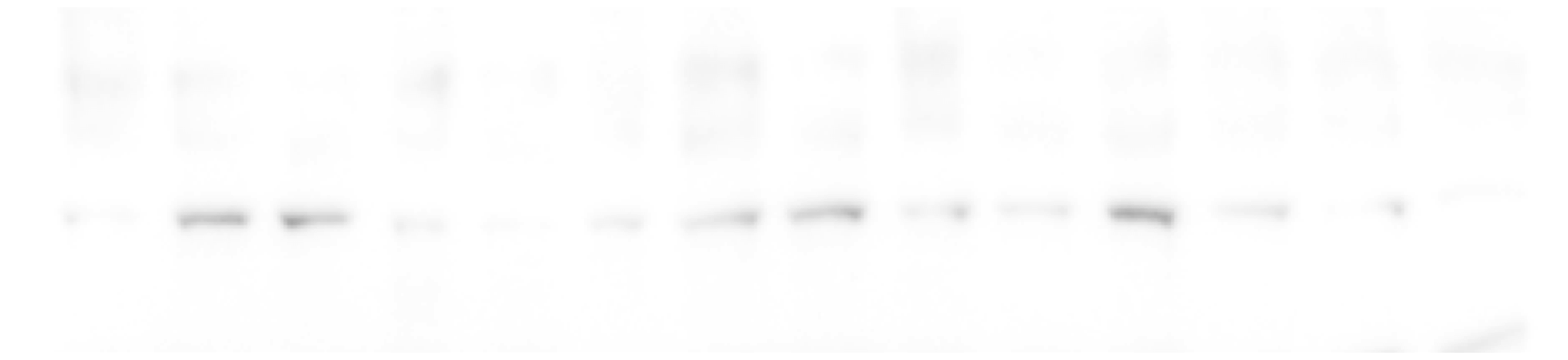

Supplement: Figure 4—source data 1. [file elife-84782-fig4-data1.zip › Fig4_MRP4.tiff]

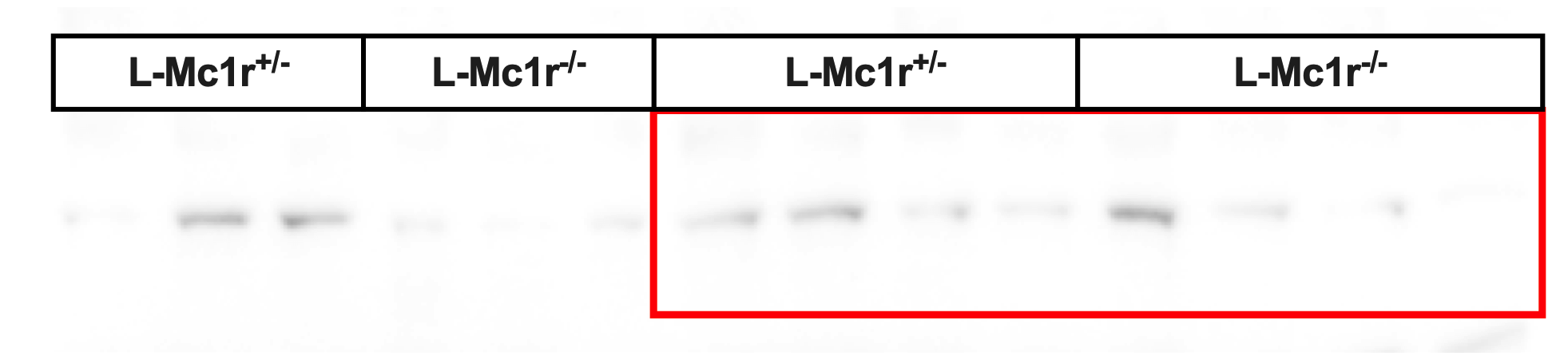

Supplement: Figure 4—source data 1. [file elife-84782-fig4-data1.zip › Fig4_MRP4_labeled.tiff]

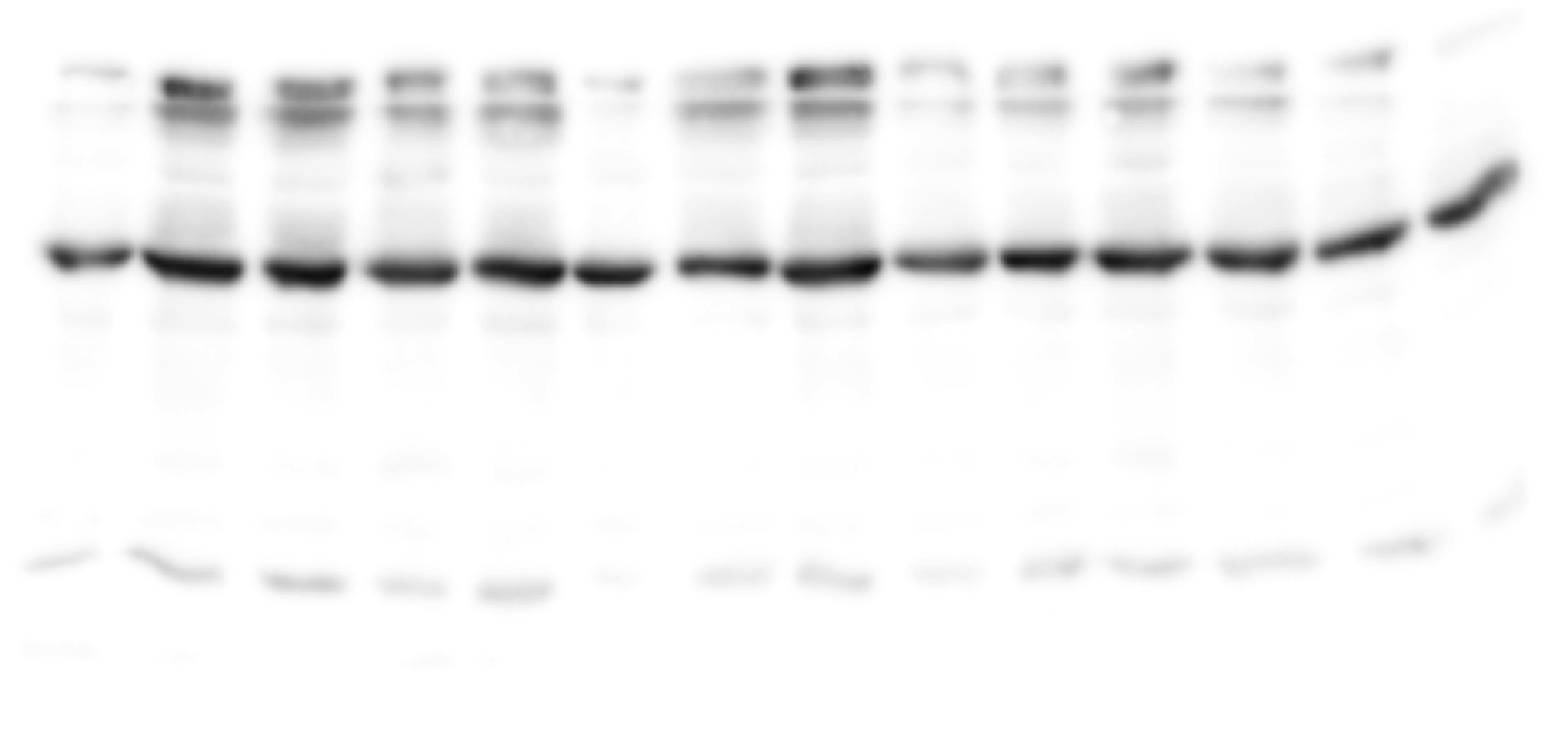

Supplement: Figure 4—source data 1. [file elife-84782-fig4-data1.zip › Fig4_b-actin.tiff]

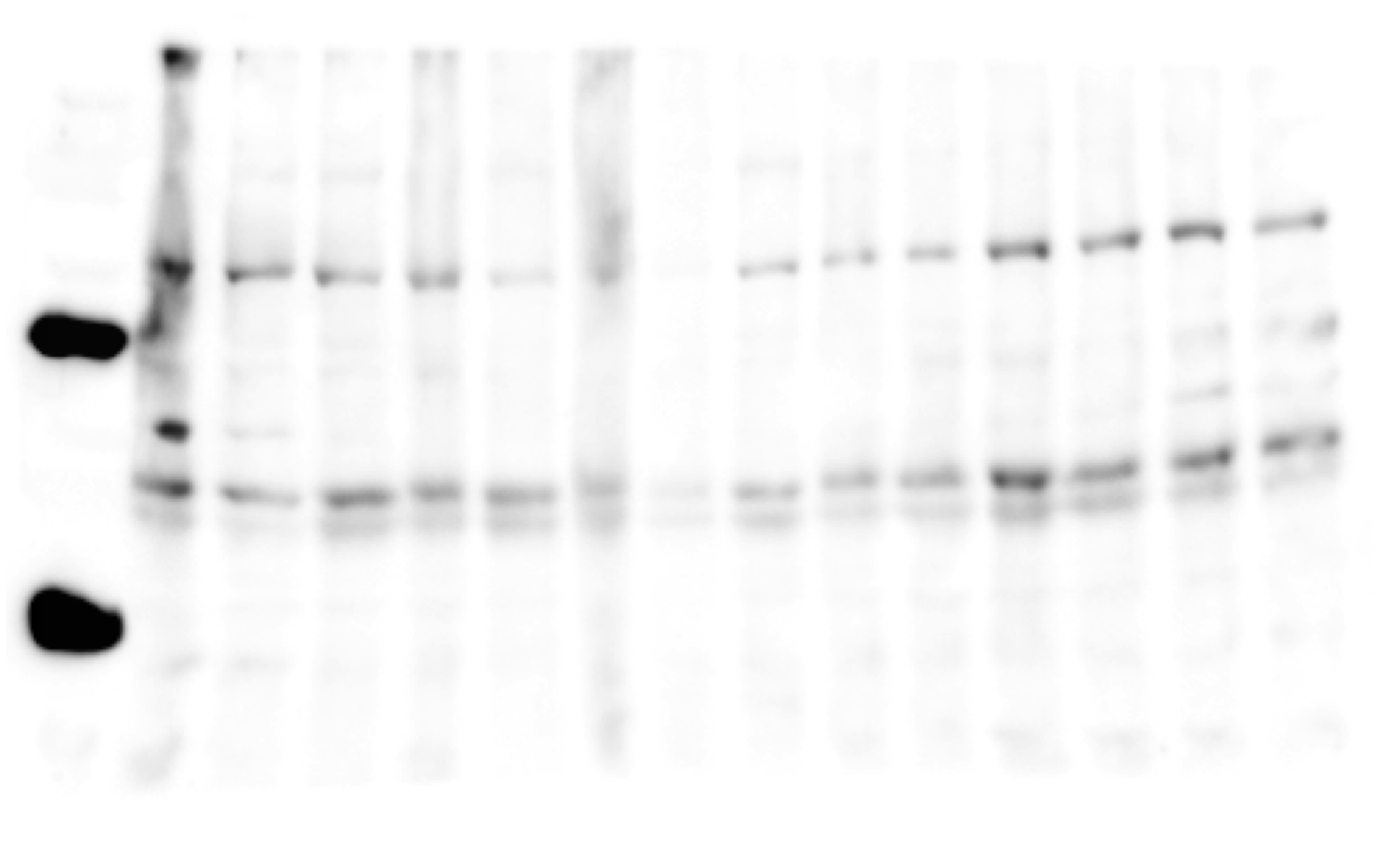

Supplement: Figure 4—source data 1. [file elife-84782-fig4-data1.zip › Fig4_CYP8B1.tiff]

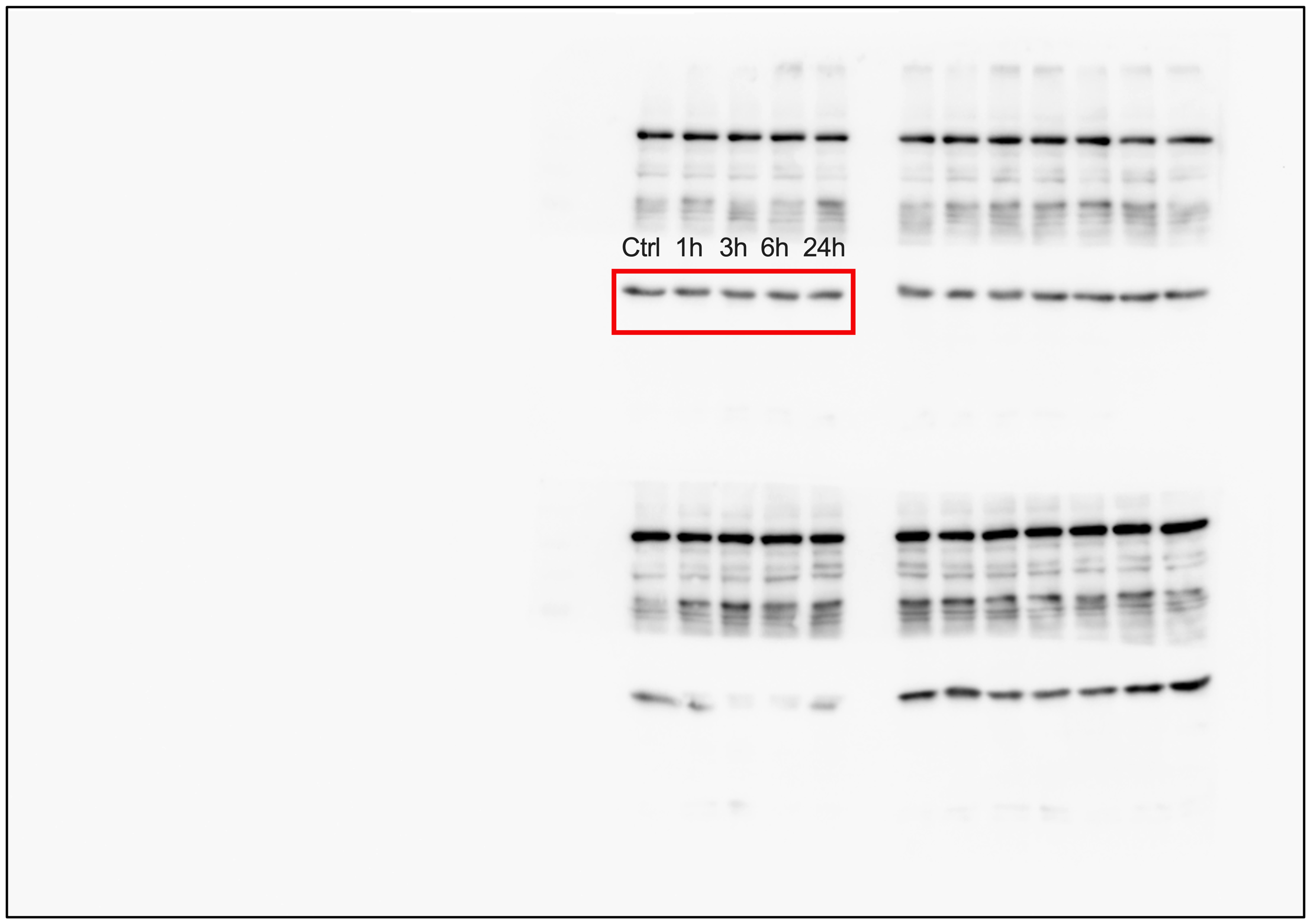

Supplement: Figure 5—source data 1. [file elife-84782-fig5-data1.zip › Figure 5J_b-actin_labelled.tif]

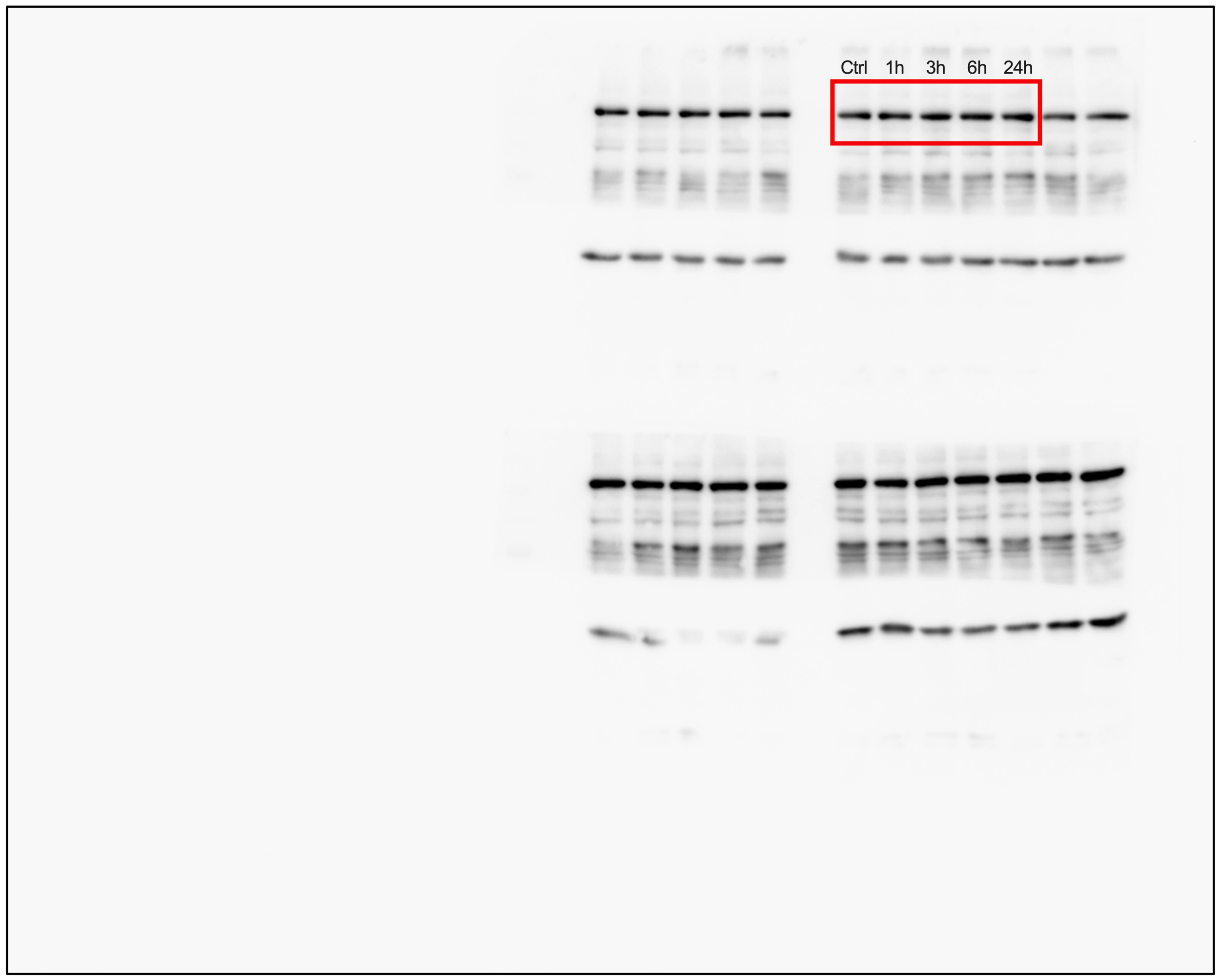

Supplement: Figure 5—source data 1. [file elife-84782-fig5-data1.zip › Figure 5J LDLR_labeled.tiff]

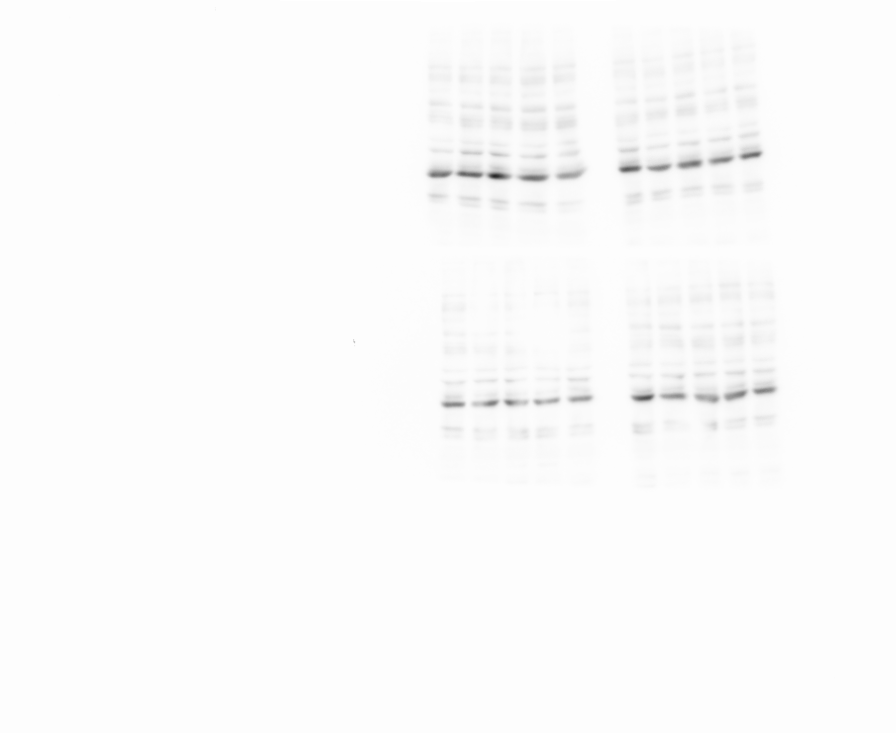

Supplement: Figure 5—source data 1. [file elife-84782-fig5-data1.zip › Figure 5C_b-actin.tif]

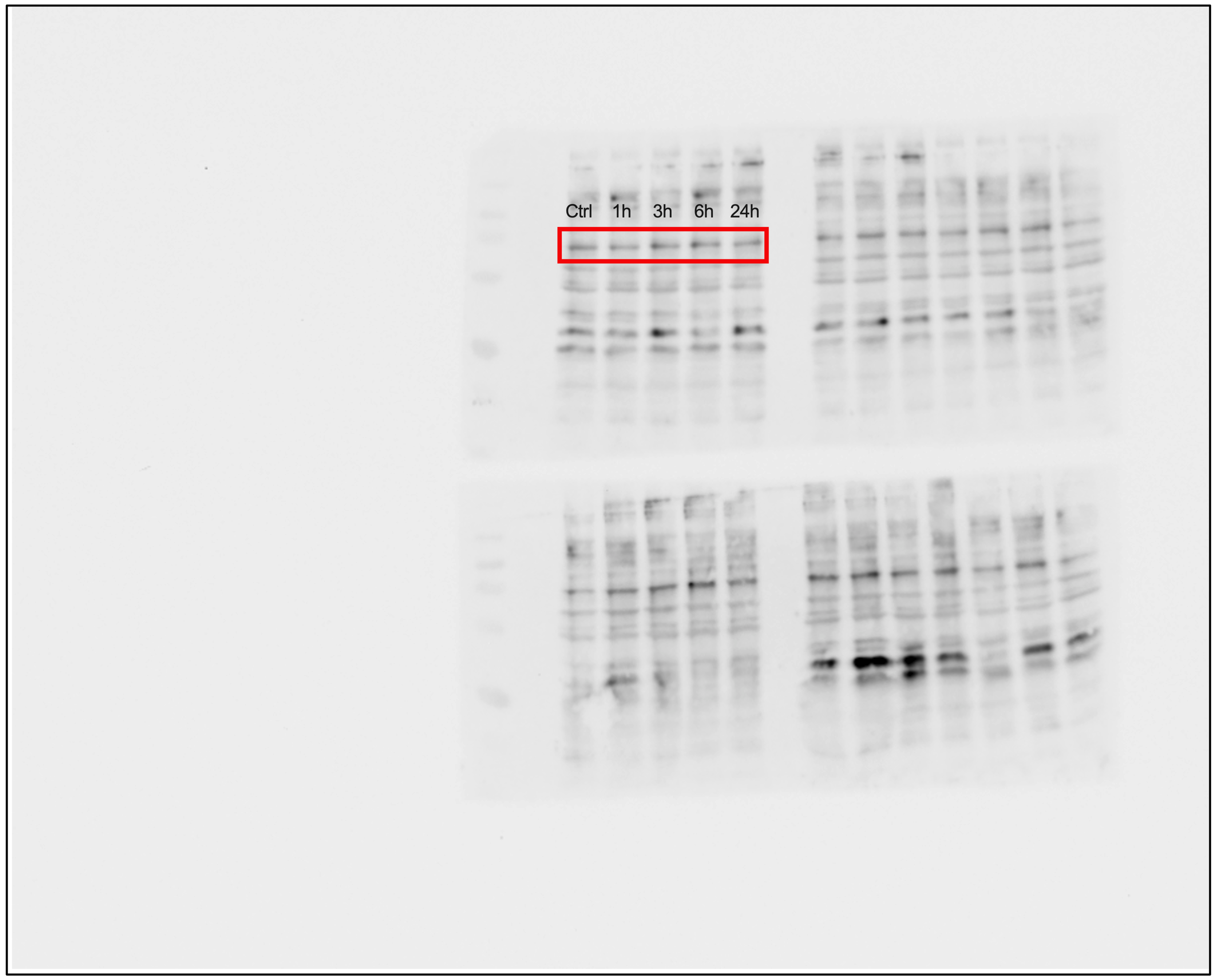

Supplement: Figure 5—source data 1. [file elife-84782-fig5-data1.zip › Figure 5J DHCR7_labeled.tiff]

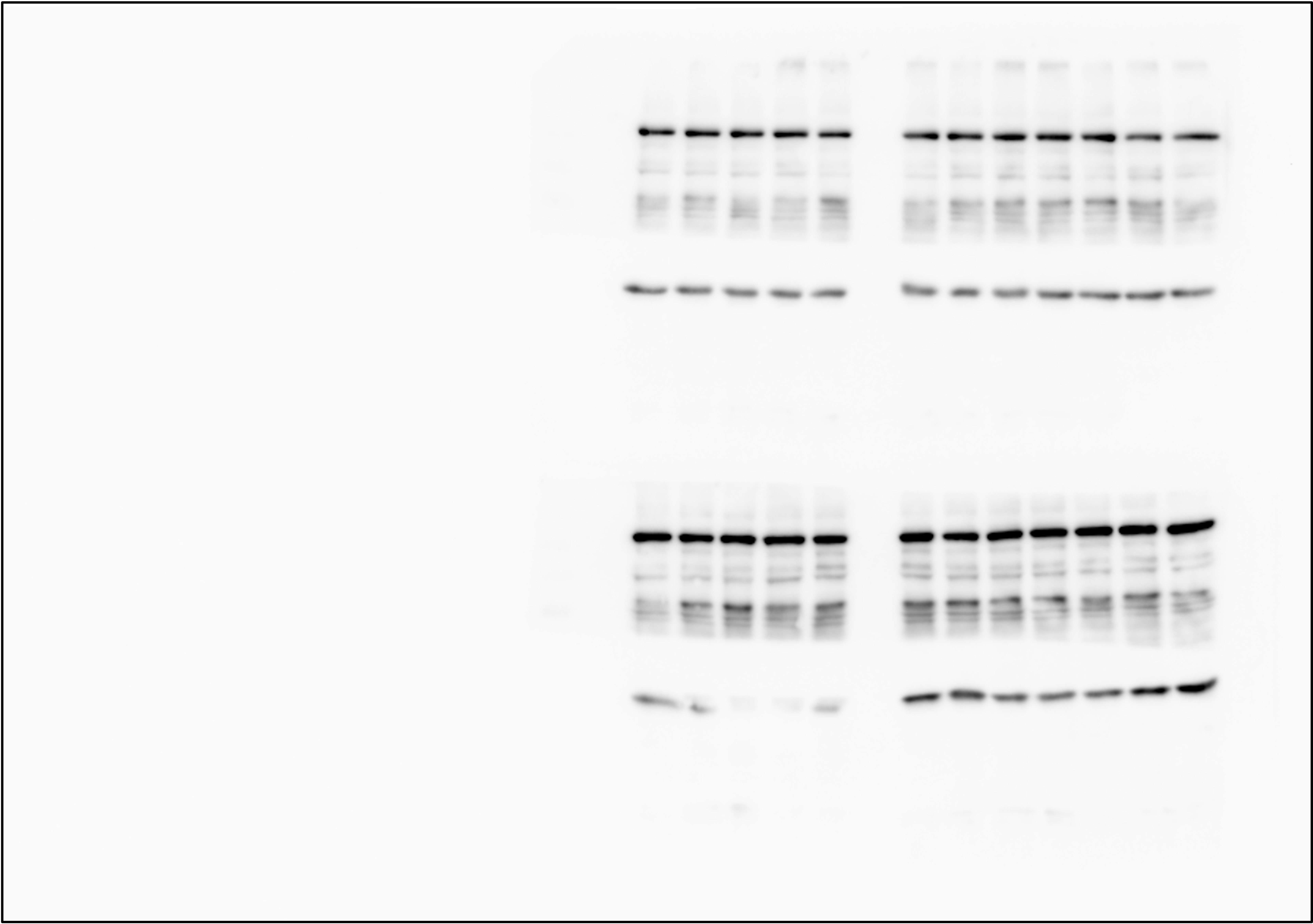

Supplement: Figure 5—source data 1. [file elife-84782-fig5-data1.zip › Figure 5J_b-actin.tif]

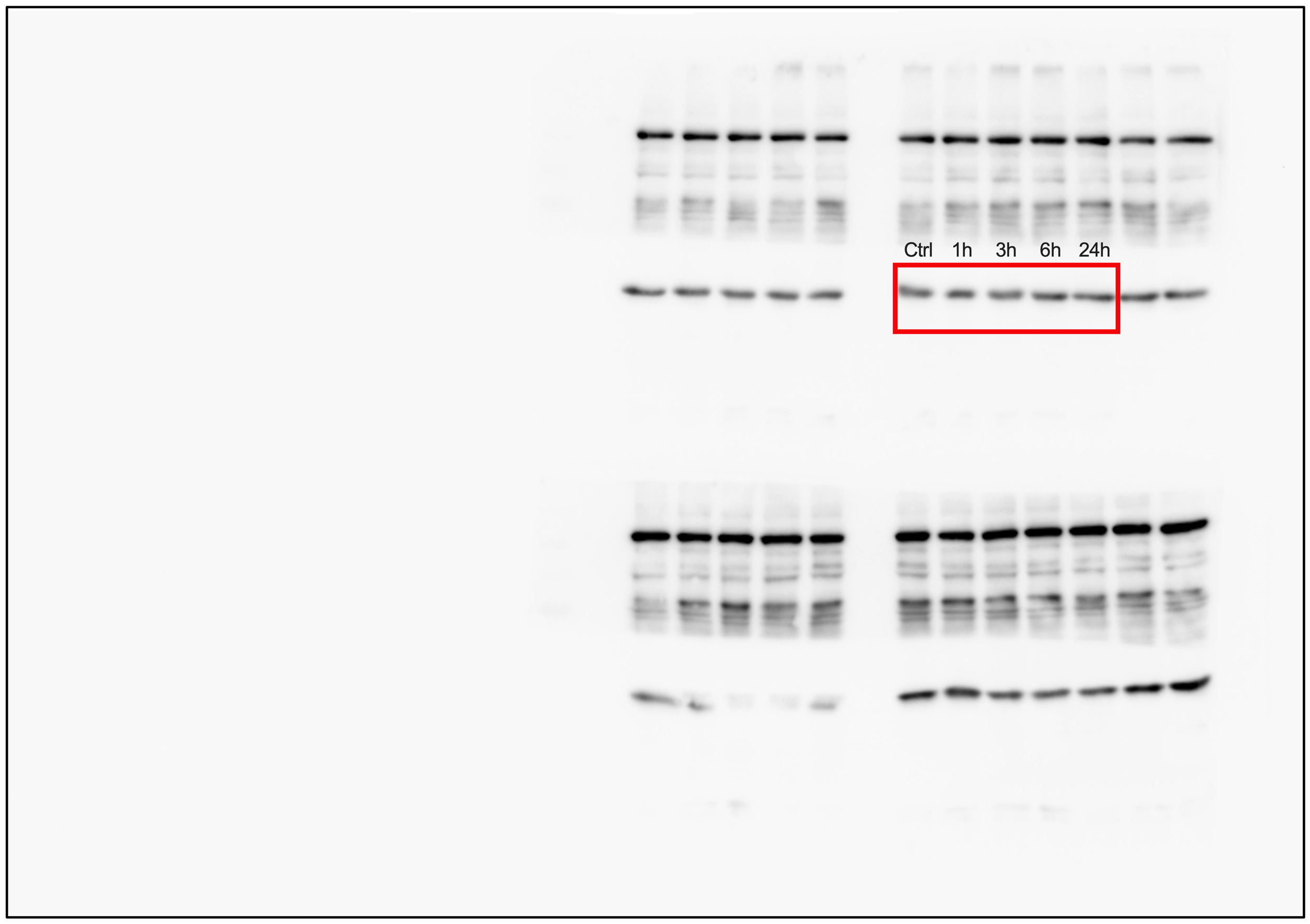

Supplement: Figure 5—source data 1. [file elife-84782-fig5-data1.zip › Figure 5J_b-actin for SR-BI_labelled.tiff]

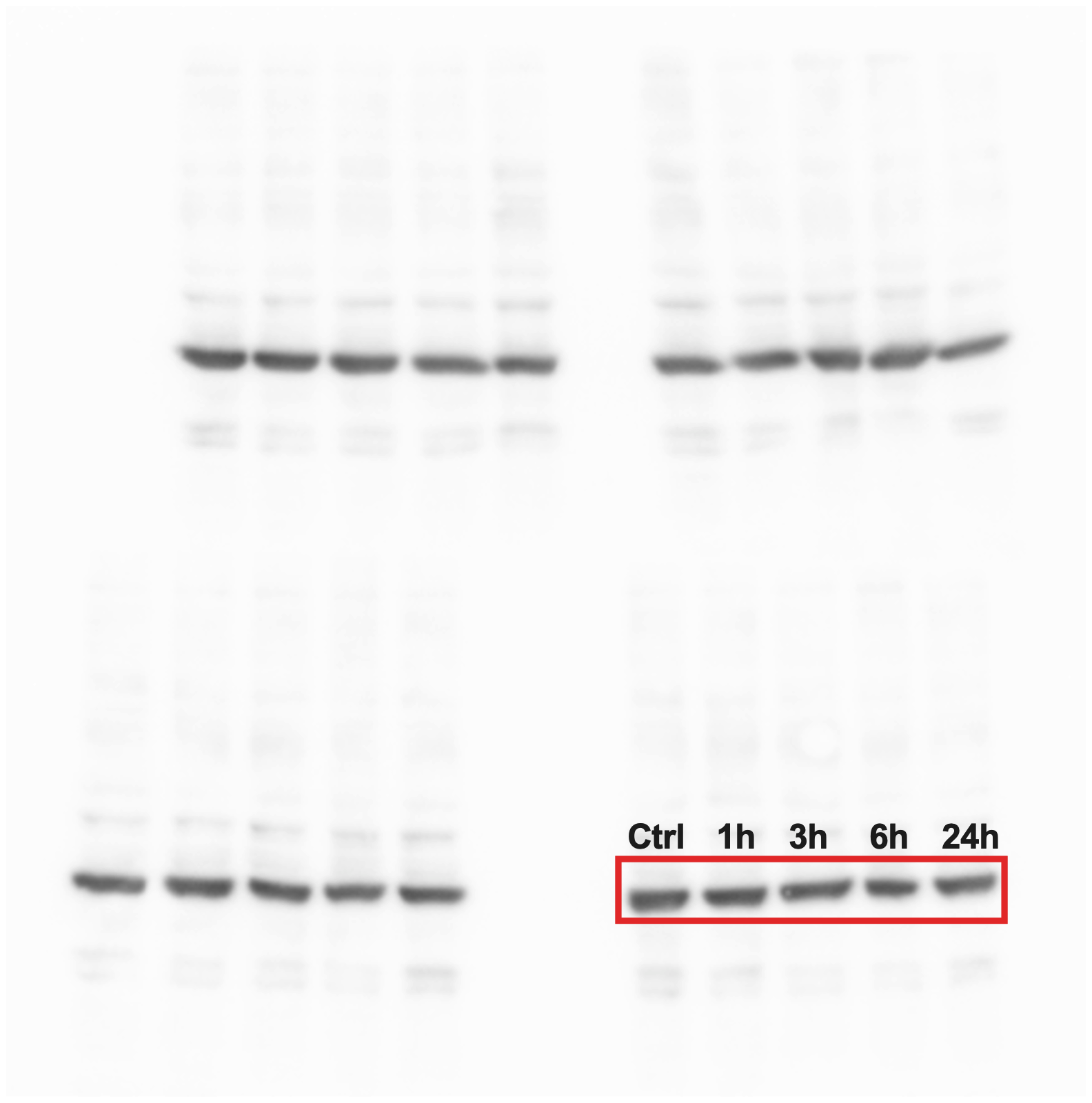

Supplement: Figure 5—source data 1. [file elife-84782-fig5-data1.zip › Figure 5A_b-actin_R1_labelled.tiff]

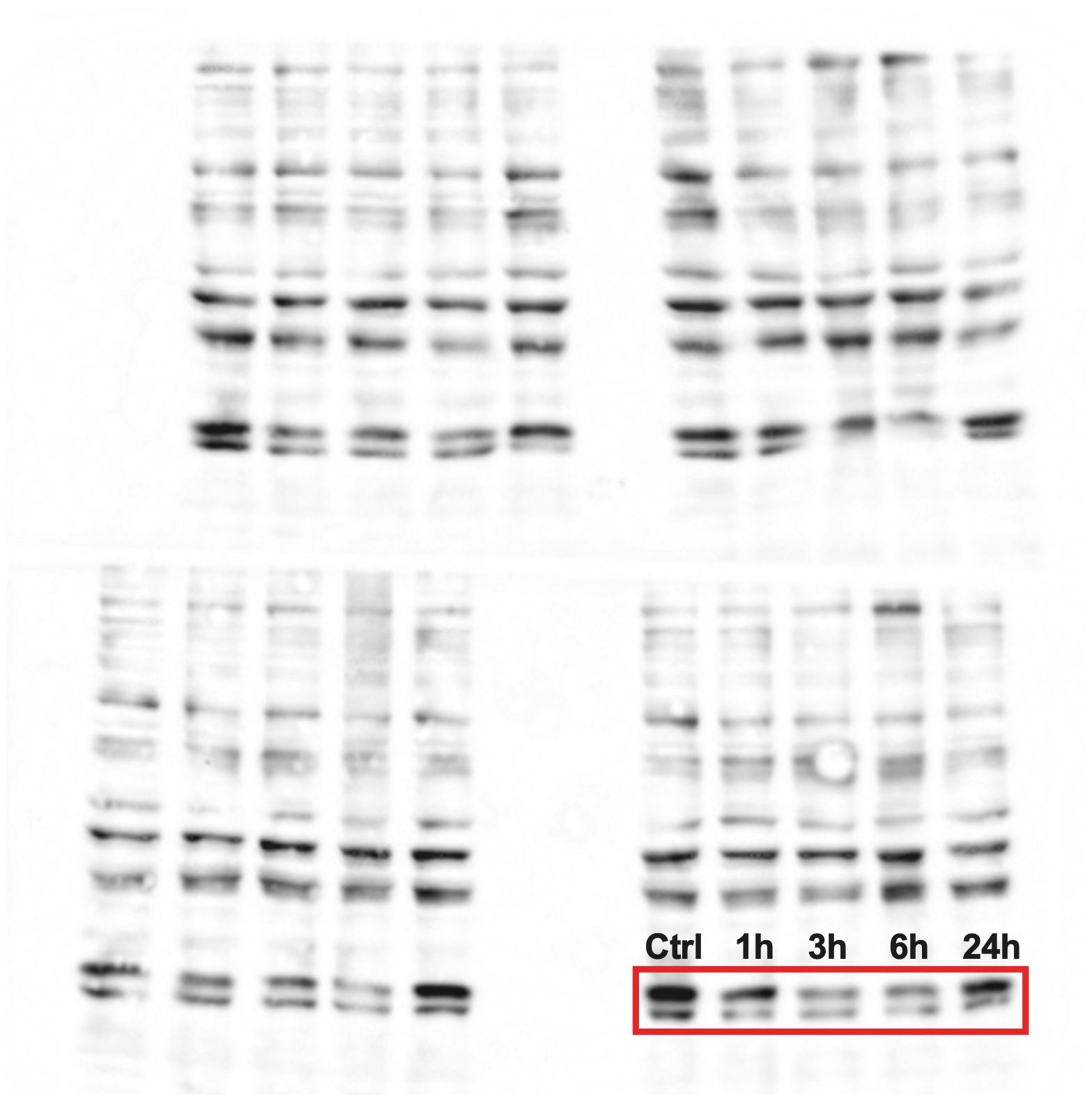

Supplement: Figure 5—source data 1. [file elife-84782-fig5-data1.zip › Figure 5A_MC1-R_labeled.tiff]

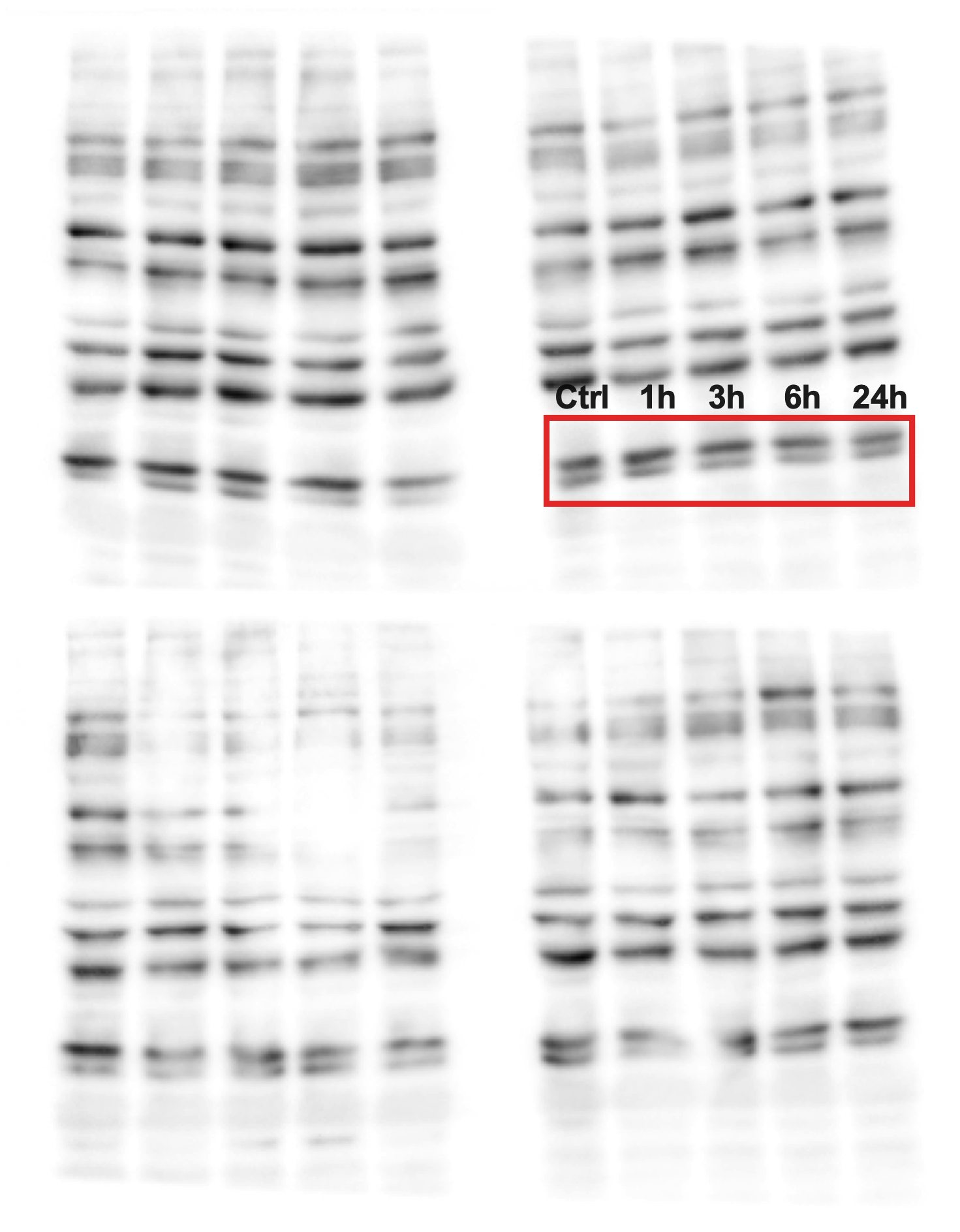

Supplement: Figure 5—source data 1. [file elife-84782-fig5-data1.zip › Figure 5C_MC1R_labelled.tiff]

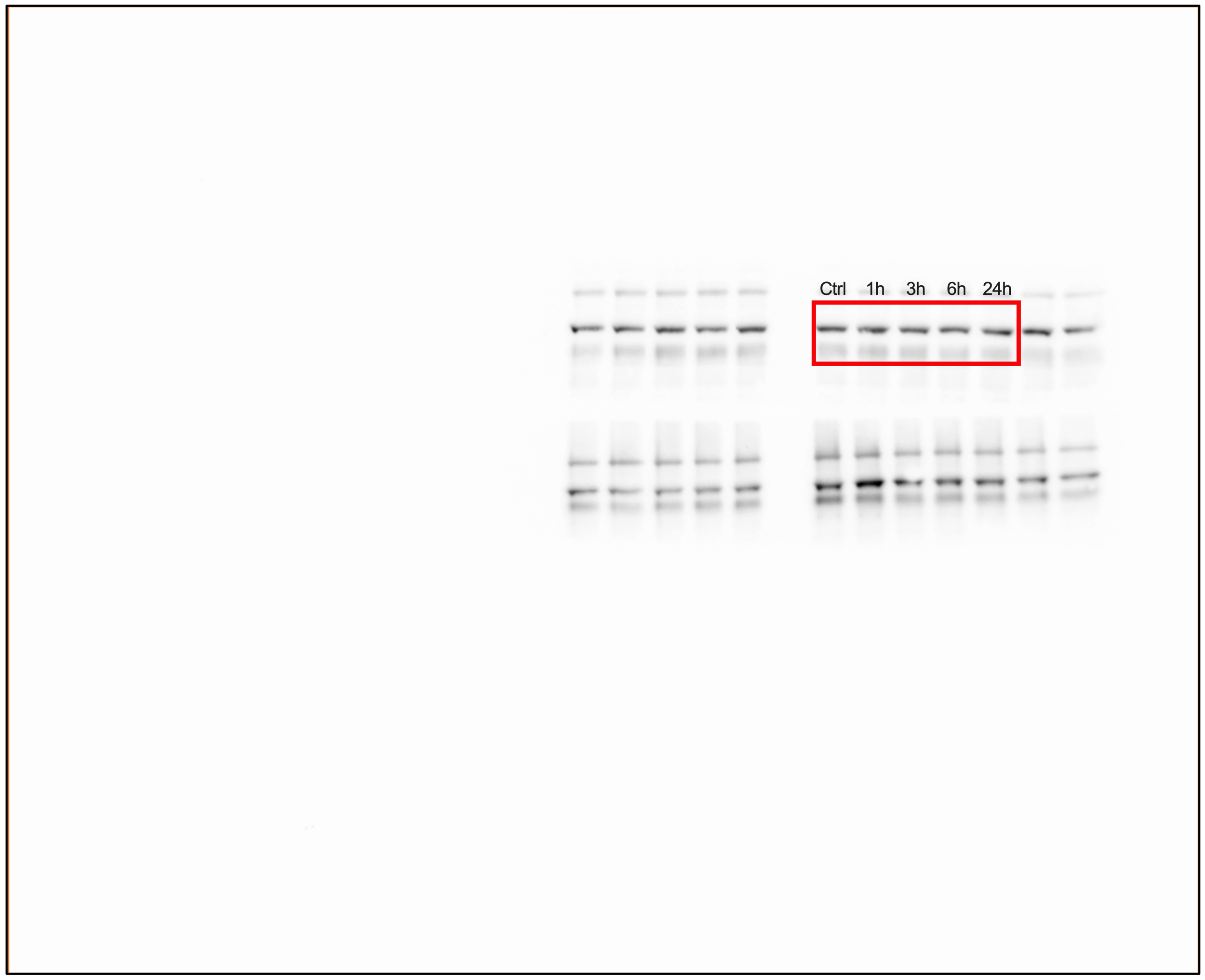

Supplement: Figure 5—source data 1. [file elife-84782-fig5-data1.zip › Figure 5J HMGCR_labeled.tiff]

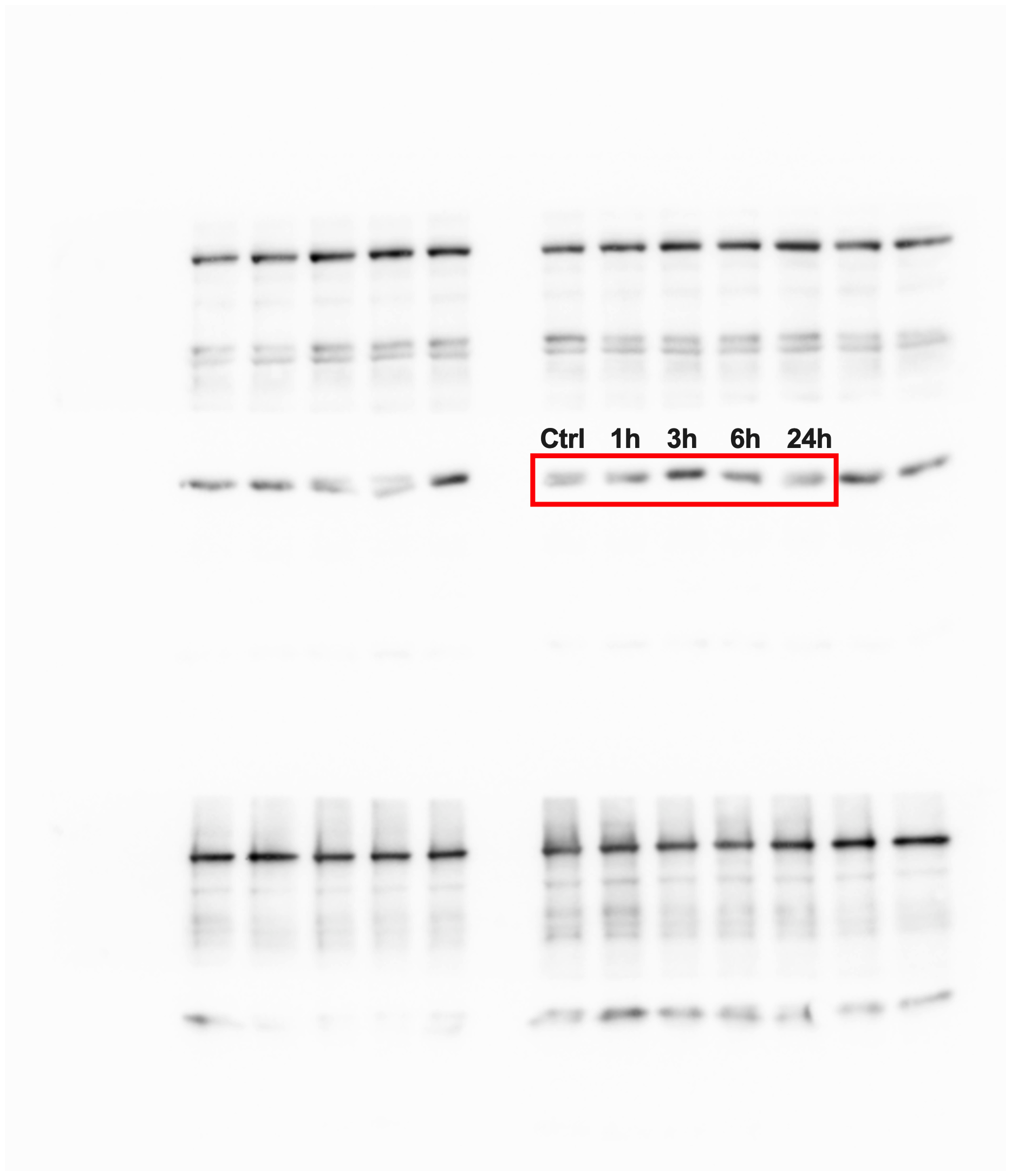

Supplement: Figure 5—source data 1. [file elife-84782-fig5-data1.zip › Figure 5J_b-actin for LDLR_labelled.tiff]

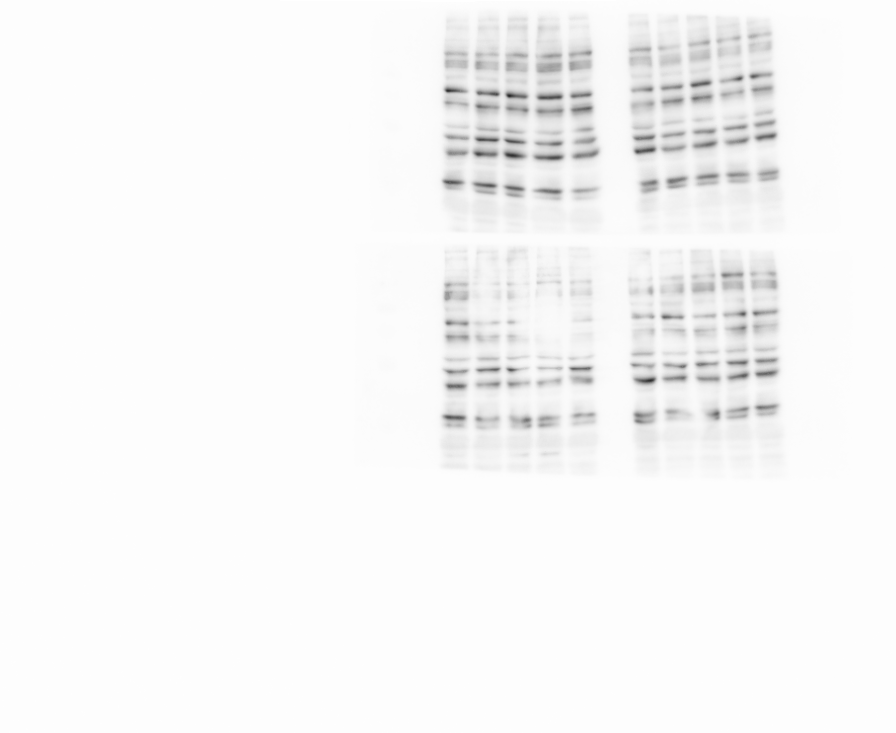

Supplement: Figure 5—source data 1. [file elife-84782-fig5-data1.zip › Figure 5C_MC1-R.tif]

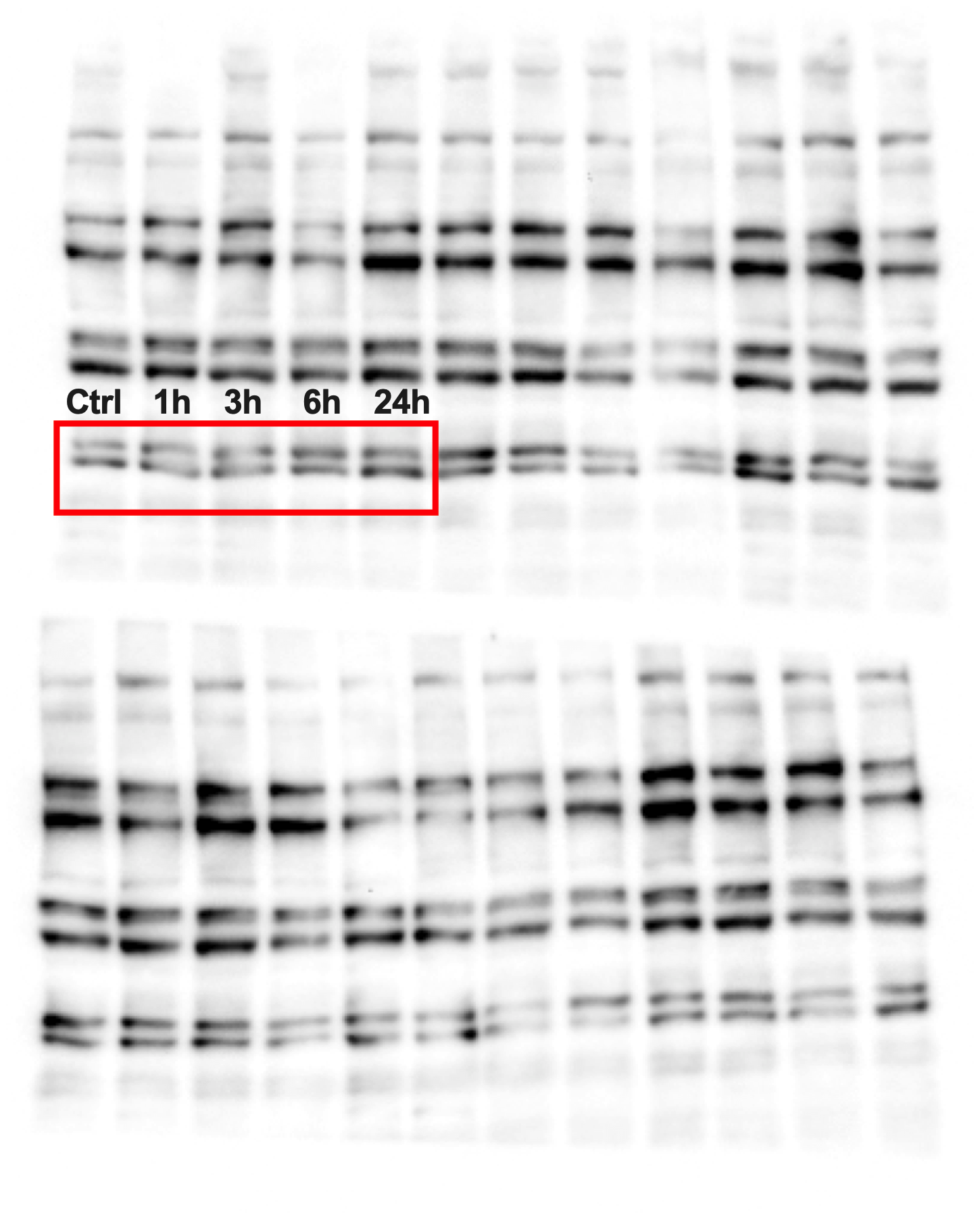

Supplement: Figure 5—source data 1. [file elife-84782-fig5-data1.zip › Figure 5B_MC1-R_R1_labelled.tiff]

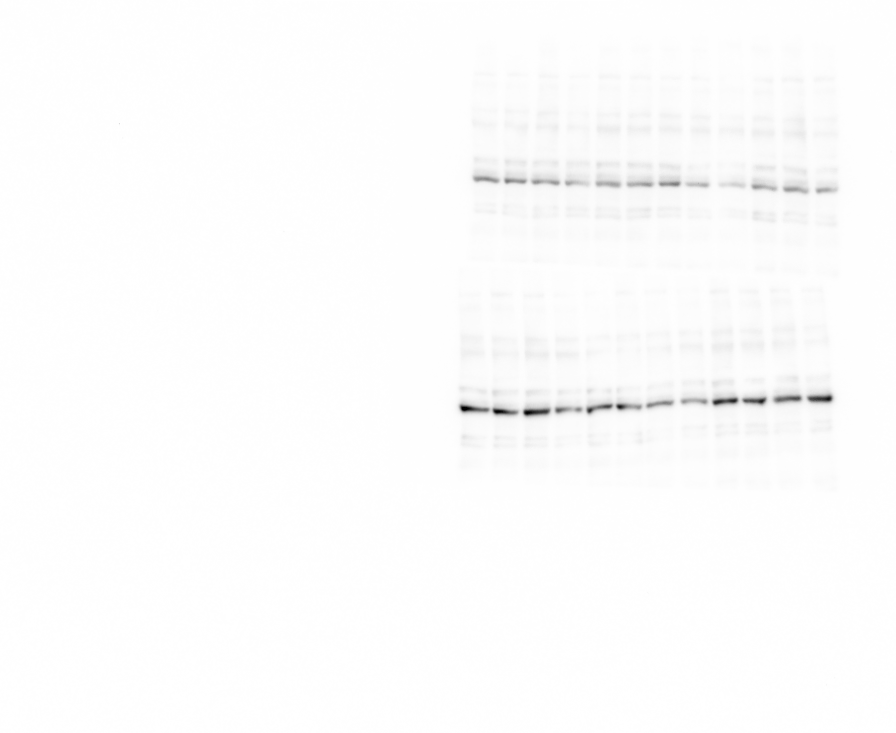

Supplement: Figure 5—source data 1. [file elife-84782-fig5-data1.zip › Figure 5B_b-actin_R1.tif]

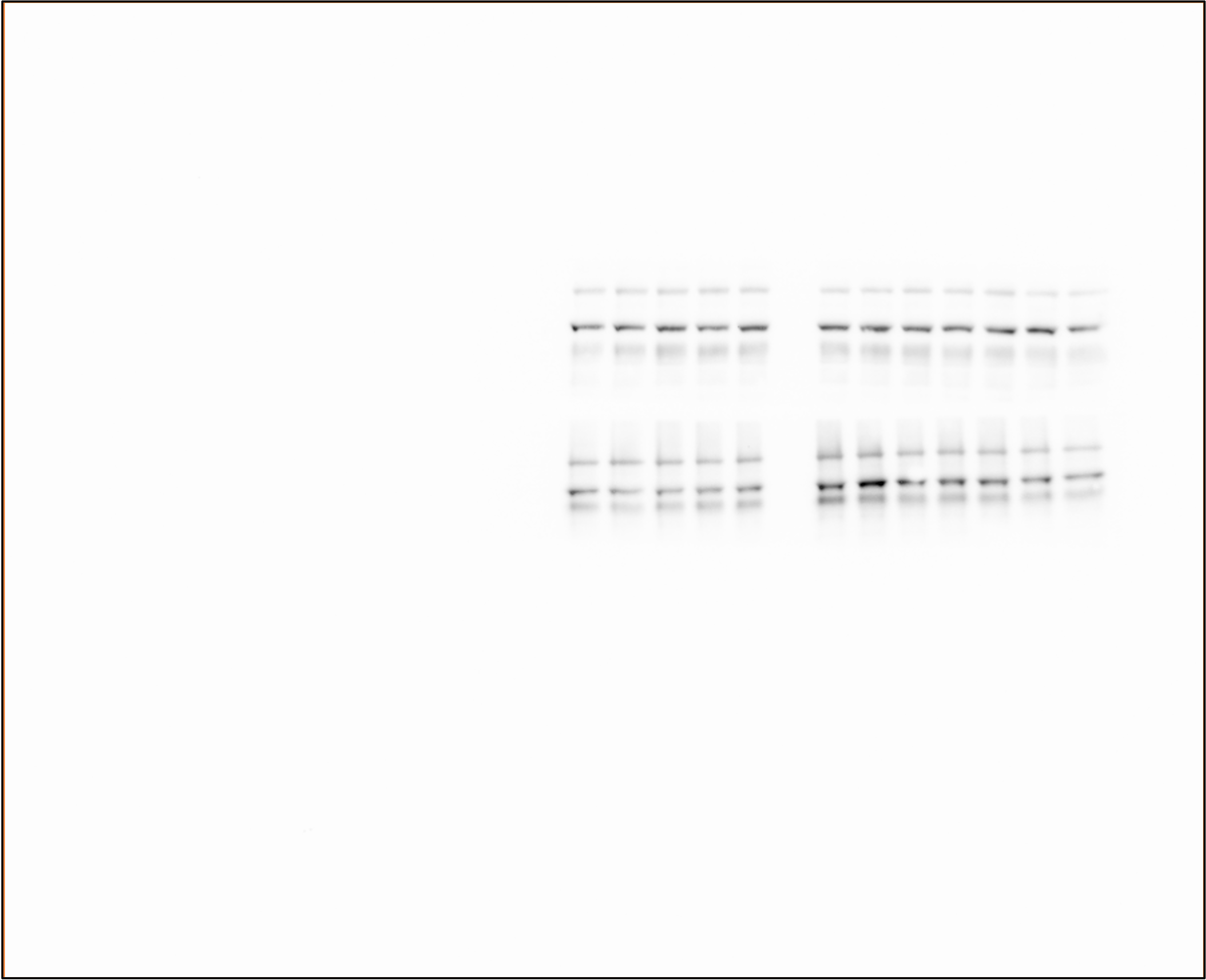

Supplement: Figure 5—source data 1. [file elife-84782-fig5-data1.zip › Figure 5J HMGCR.tiff]

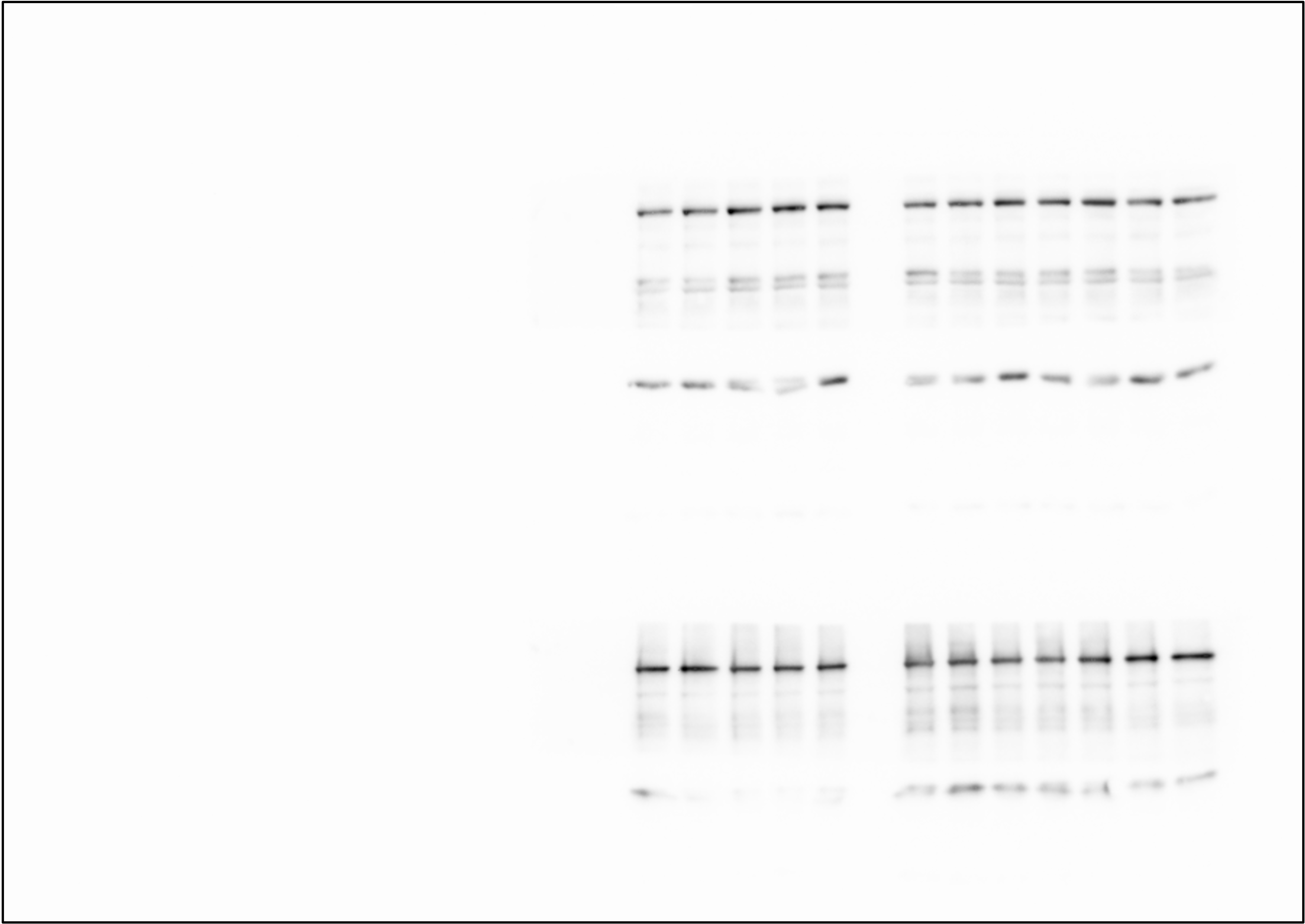

Supplement: Figure 5—source data 1. [file elife-84782-fig5-data1.zip › Figure 5J_b-actin for LDLR.tiff]

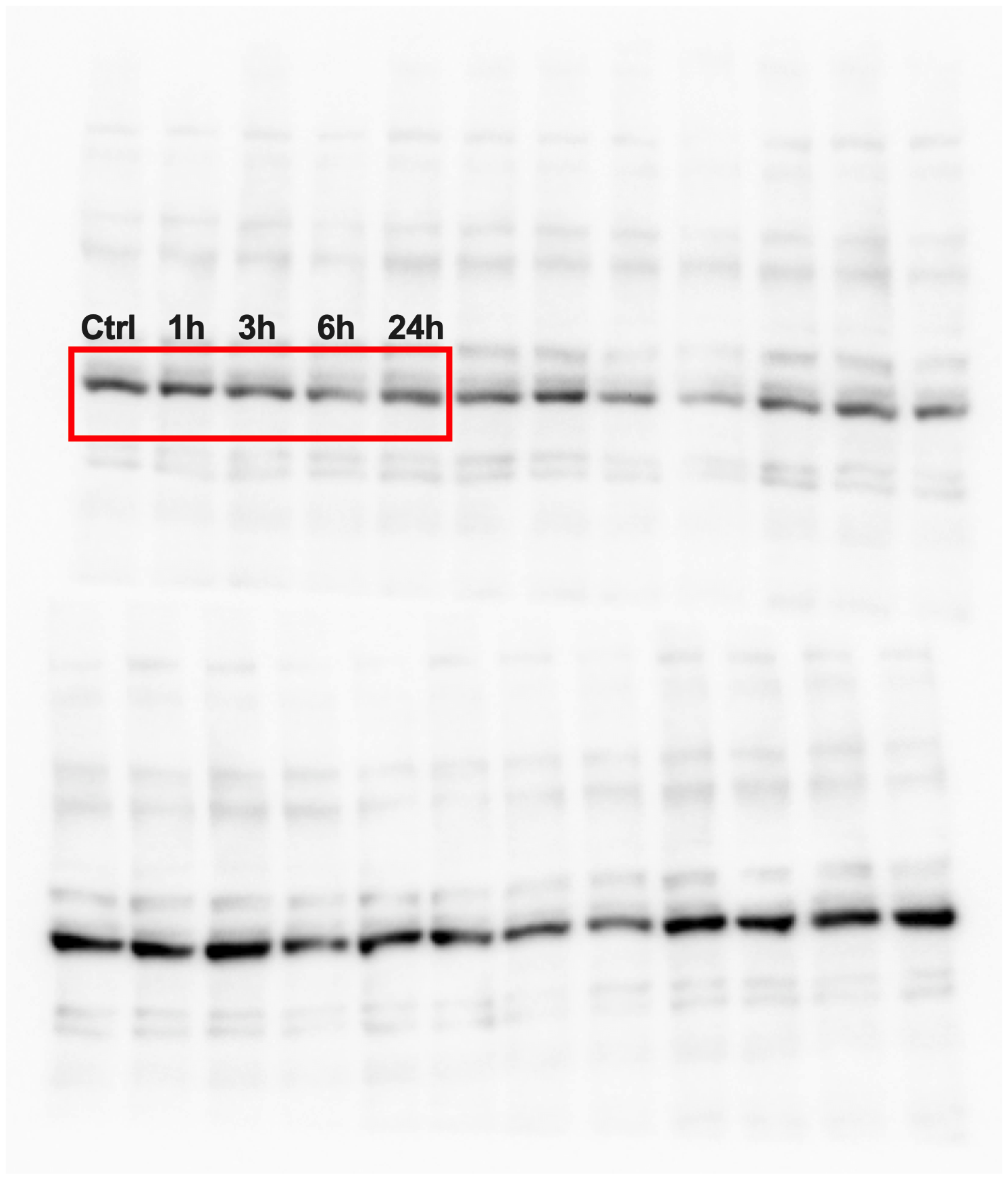

Supplement: Figure 5—source data 1. [file elife-84782-fig5-data1.zip › Figure 5B_b-actin_labelled_R1.tiff]

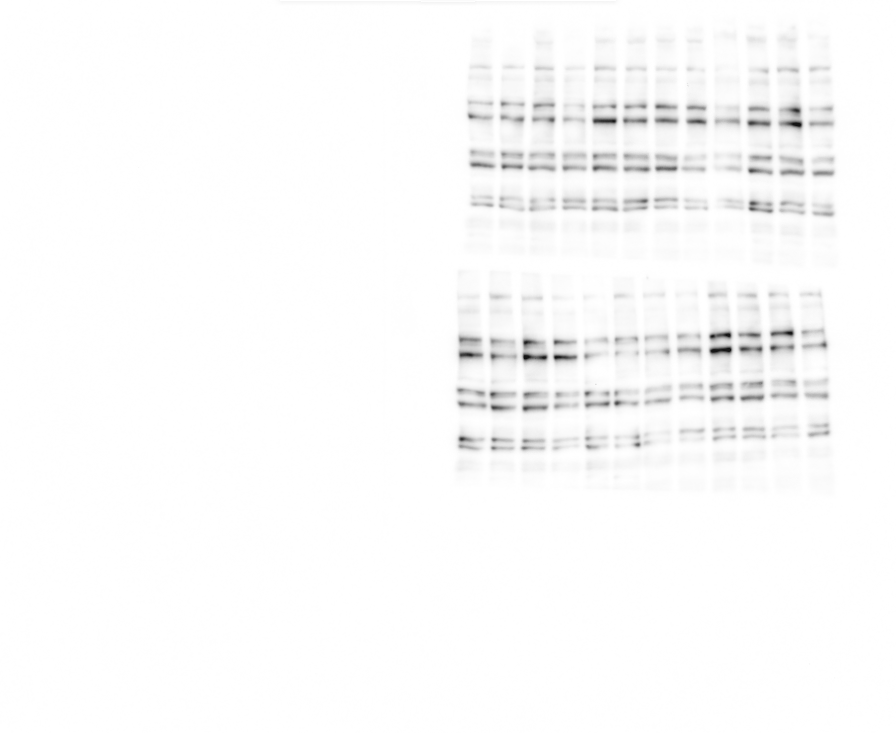

Supplement: Figure 5—source data 1. [file elife-84782-fig5-data1.zip › Figure 5B_MC1-R_R1.tif]

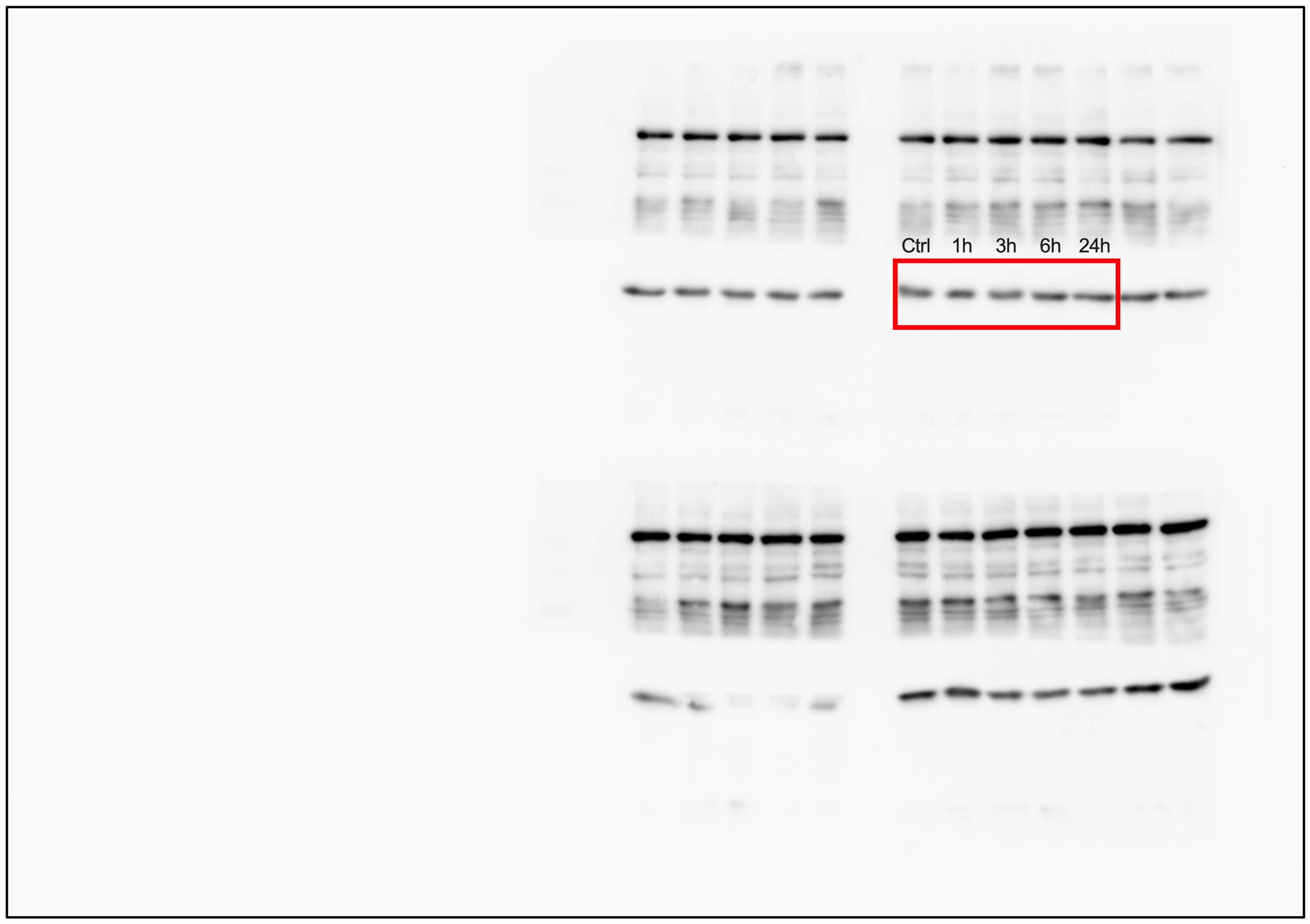

Supplement: Figure 5—source data 1. [file elife-84782-fig5-data1.zip › Figure 5J_b-actin for HMGCR_labelled.tiff]

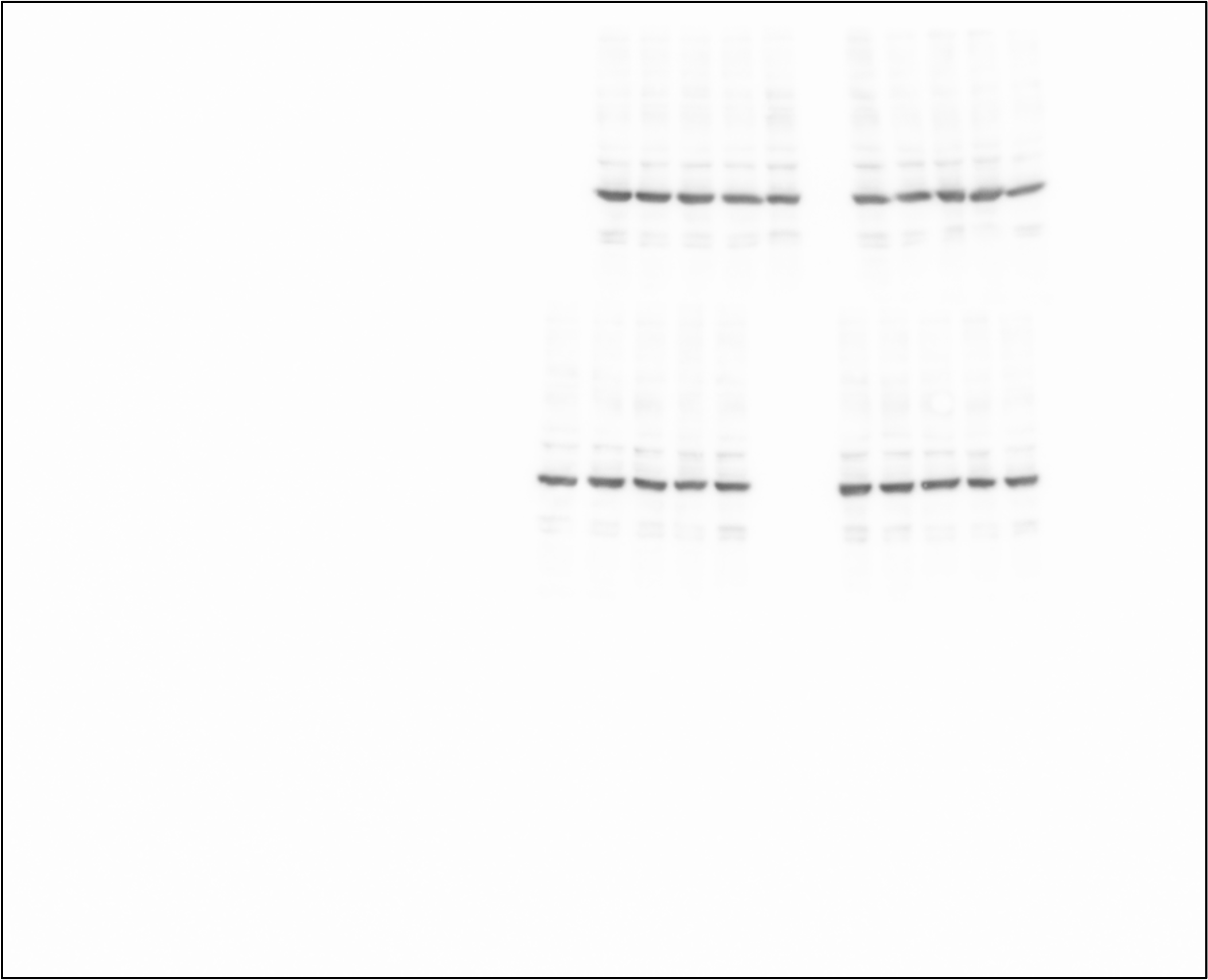

Supplement: Figure 5—source data 1. [file elife-84782-fig5-data1.zip › Figure 5A_b-actin.tiff]

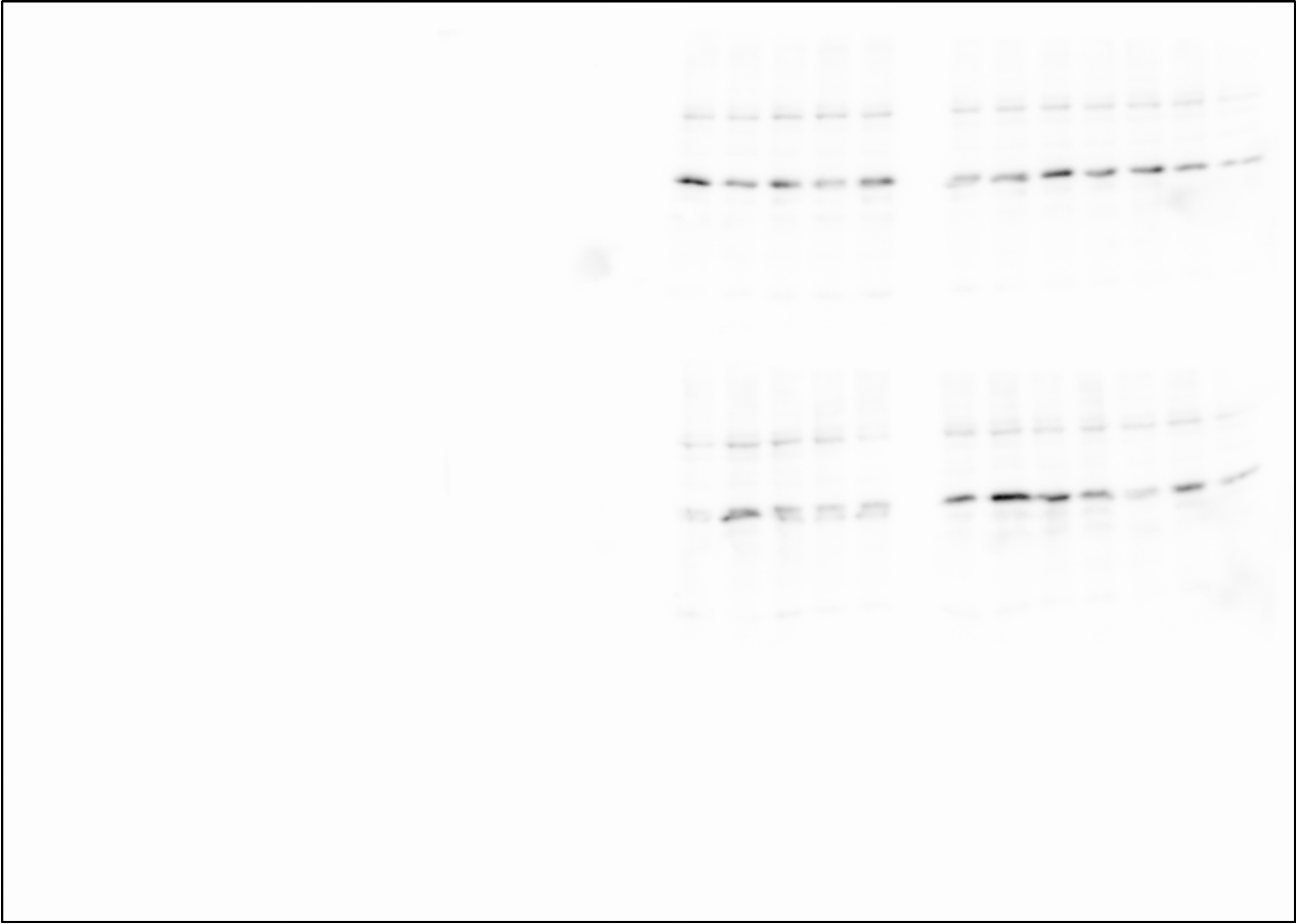

Supplement: Figure 5—source data 1. [file elife-84782-fig5-data1.zip › Figure 5J_b-actin for DHCR7.tiff]

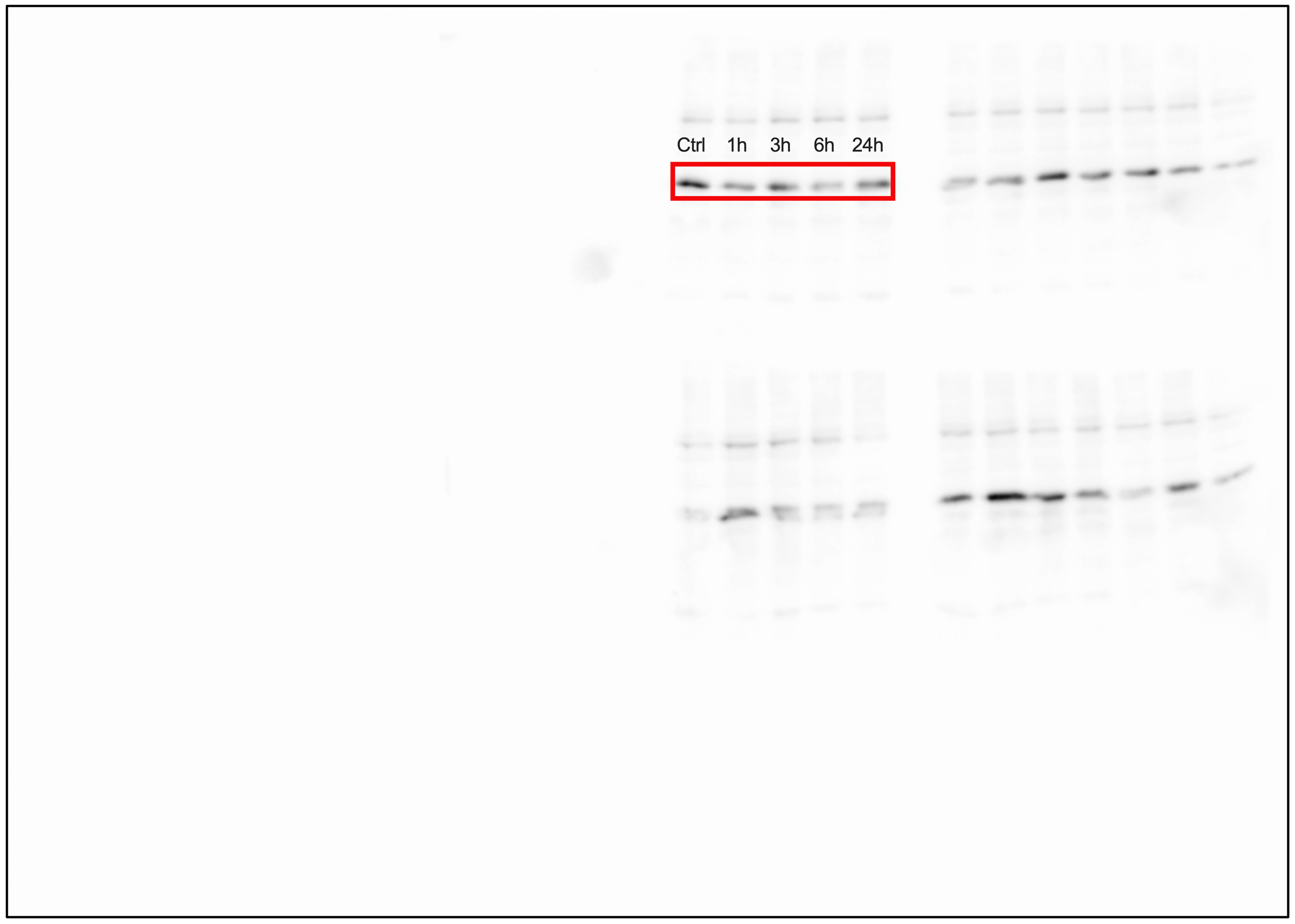

Supplement: Figure 5—source data 1. [file elife-84782-fig5-data1.zip › Figure 5J_b-actin for DHCR7_labelled.tiff]

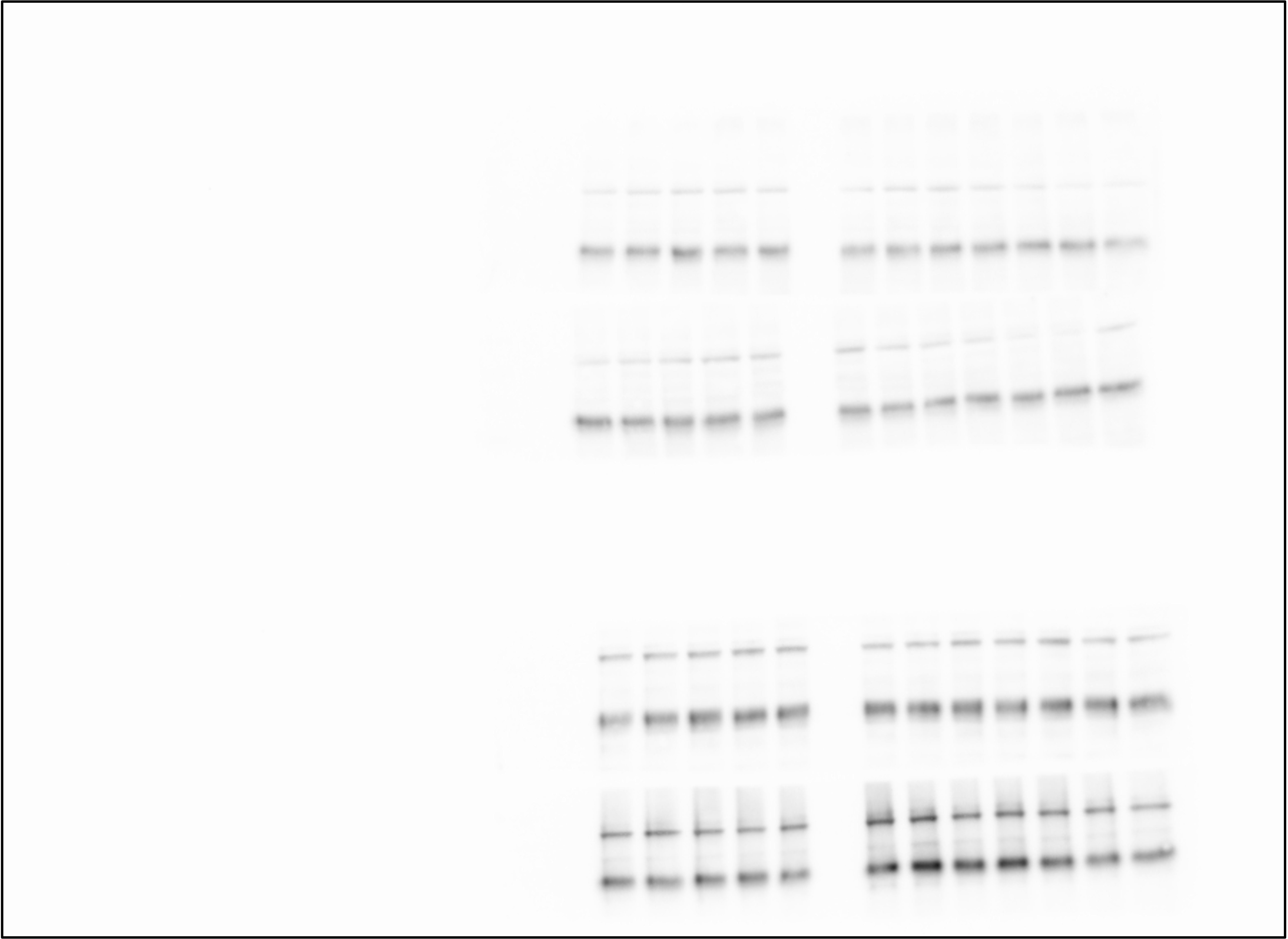

Supplement: Figure 5—source data 1. [file elife-84782-fig5-data1.zip › Figure 5J SR-BI.tiff]

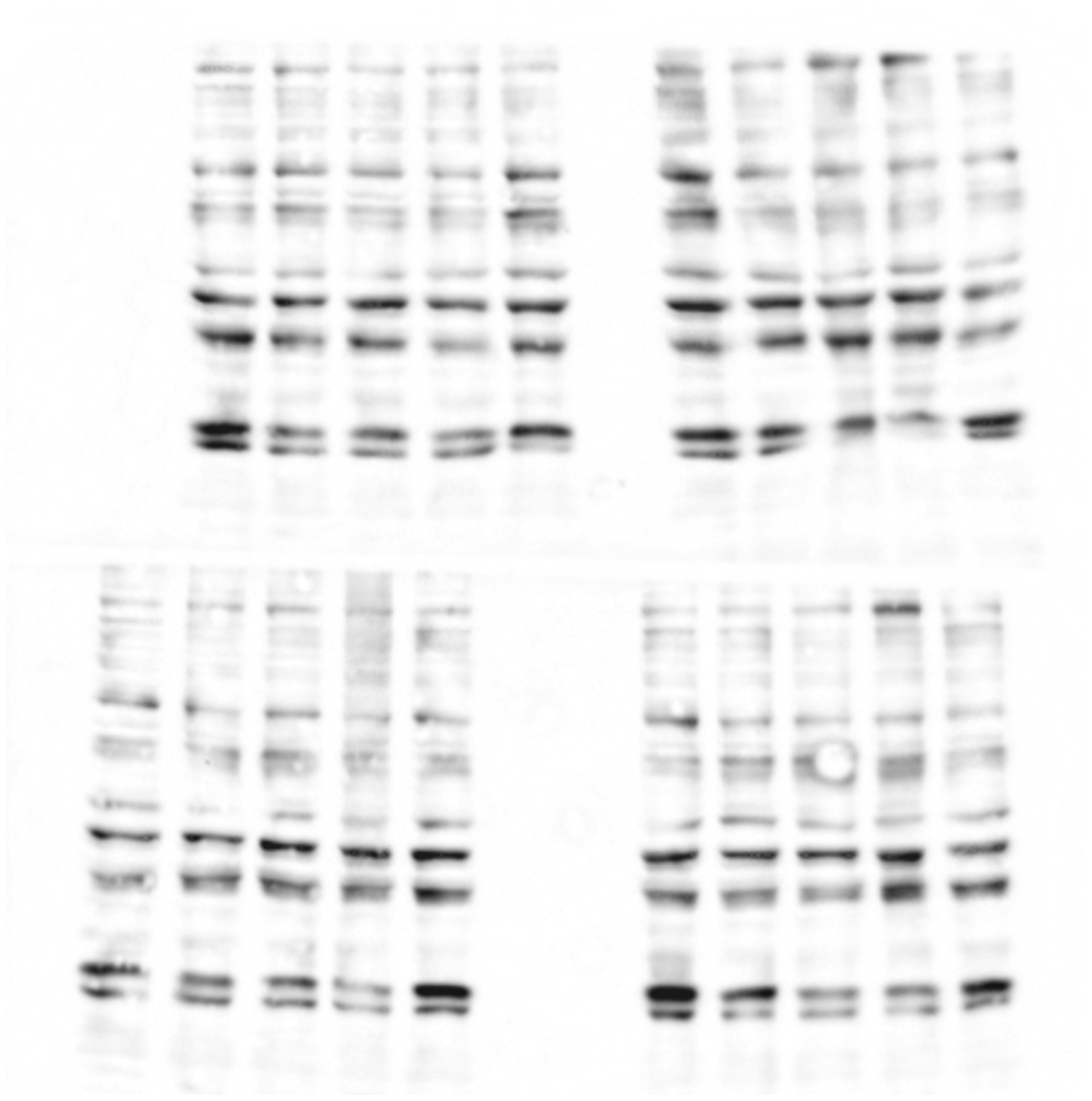

Supplement: Figure 5—source data 1. [file elife-84782-fig5-data1.zip › Figure 5A_MC1-R_R1.tiff]

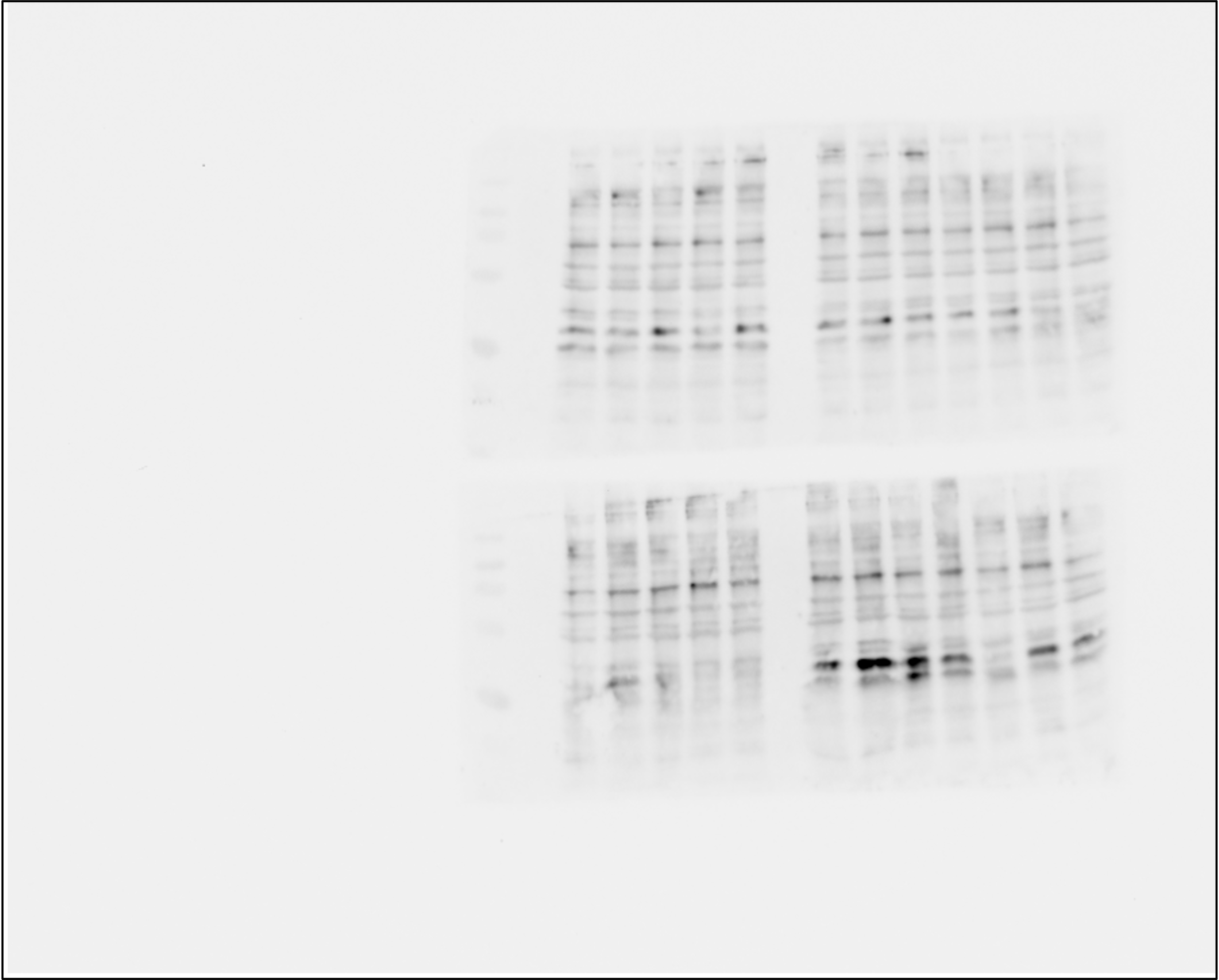

Supplement: Figure 5—source data 1. [file elife-84782-fig5-data1.zip › Figure 5J DHCR7.tiff]

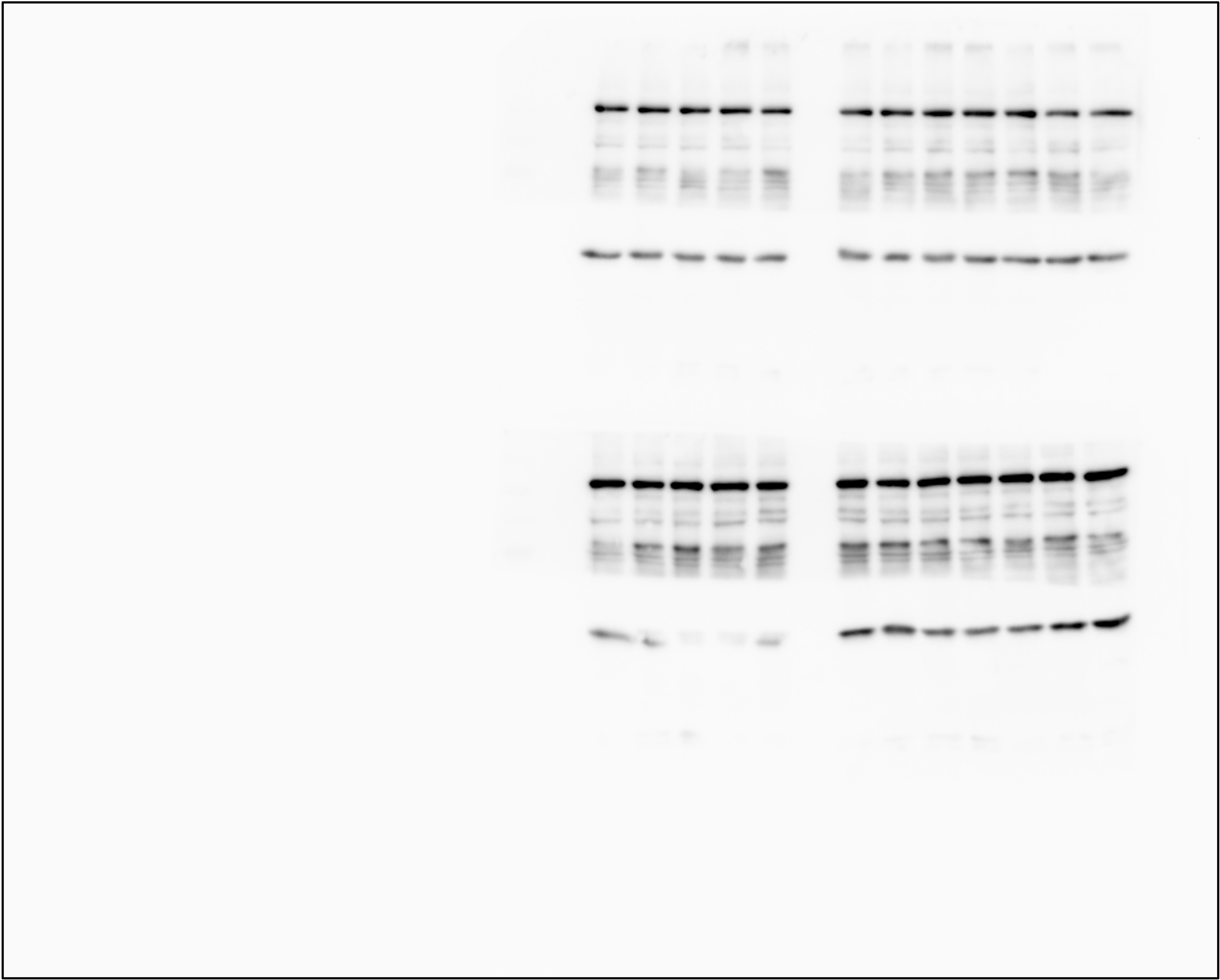

Supplement: Figure 5—source data 1. [file elife-84782-fig5-data1.zip › Figure 5J LDLR.tiff]

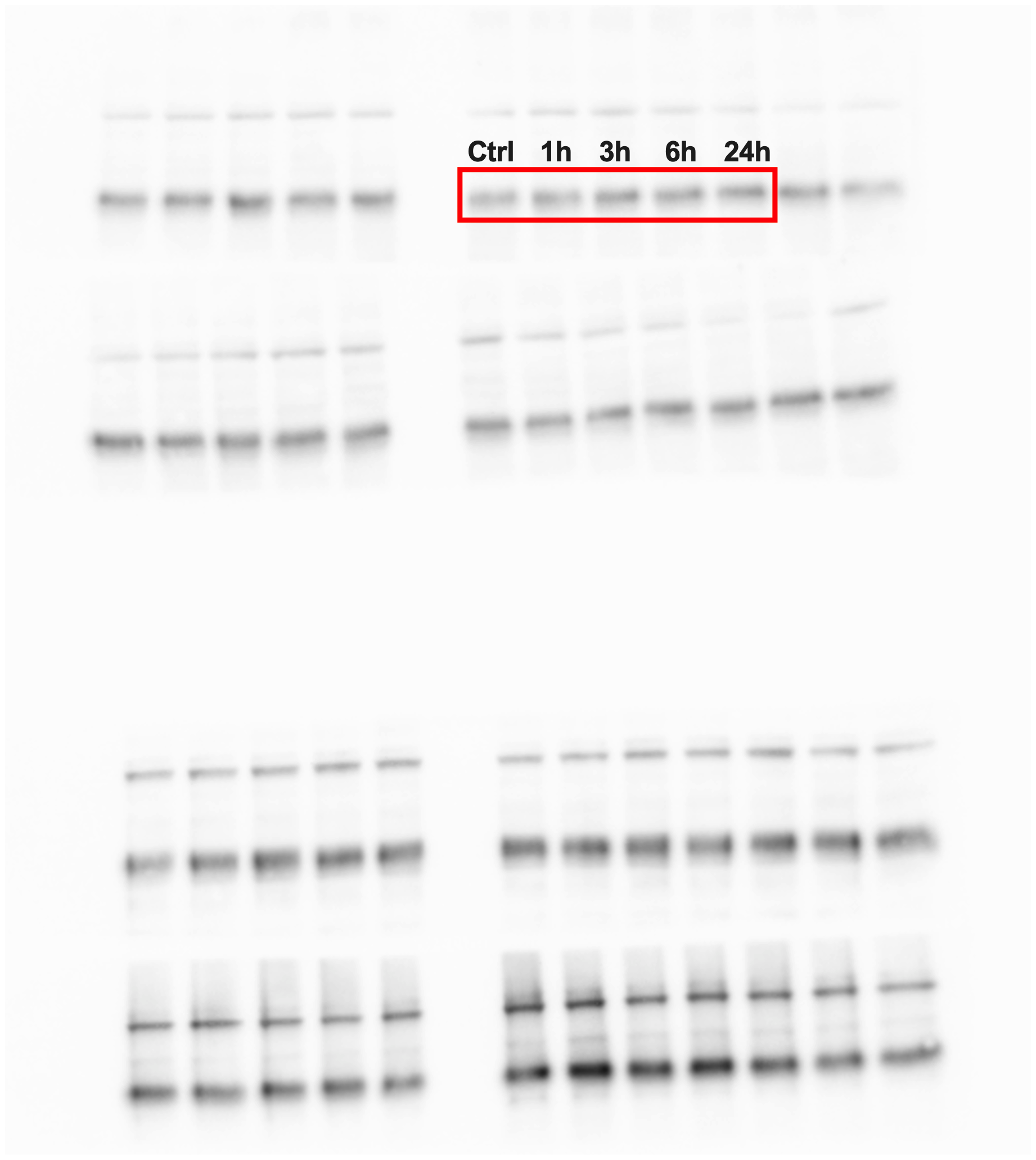

Supplement: Figure 5—source data 1. [file elife-84782-fig5-data1.zip › Figure 5J SR-BI_labeled.tiff]

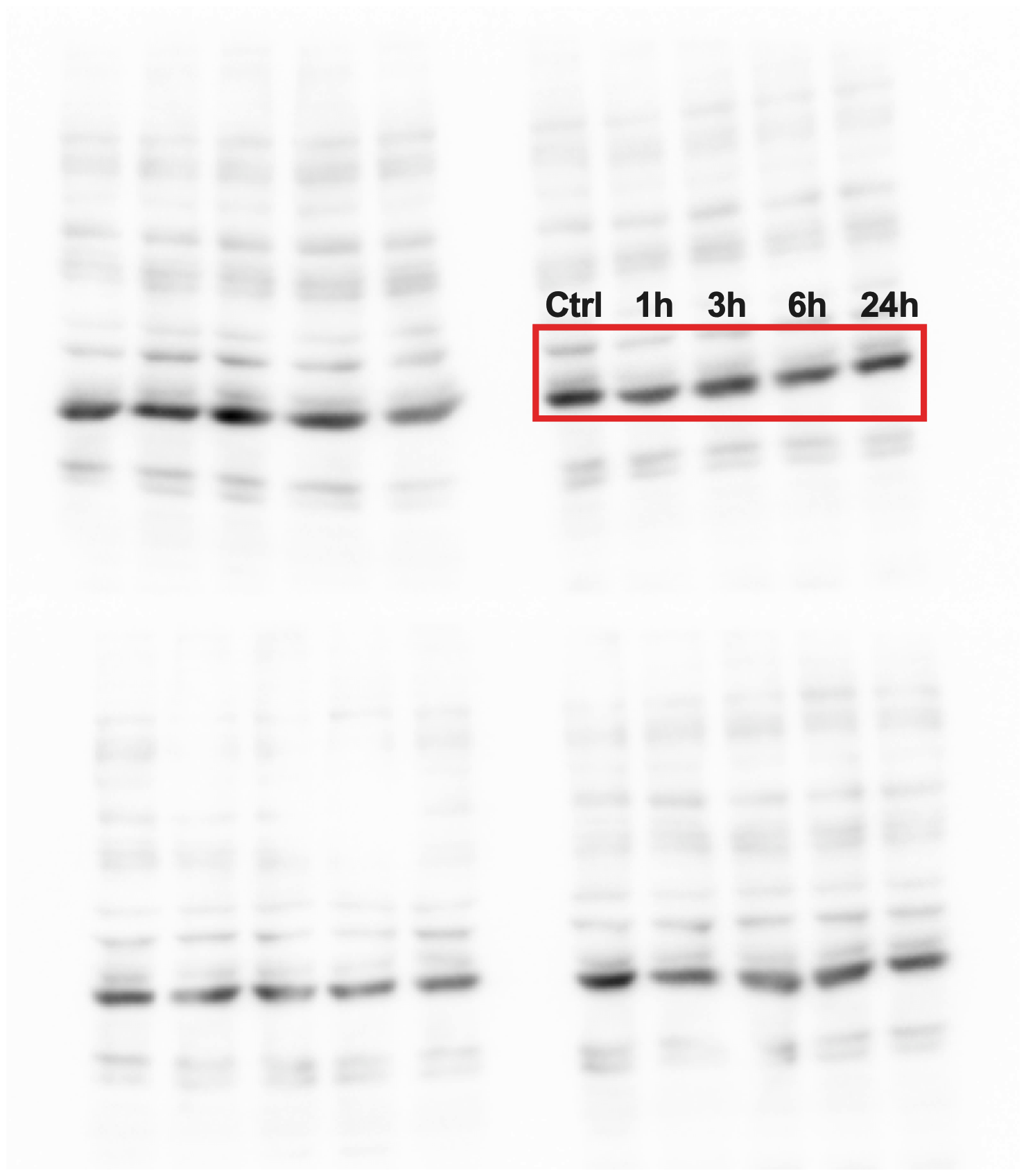

Supplement: Figure 5—source data 1. [file elife-84782-fig5-data1.zip › Figure 5C_b-actin_labelled.tiff]

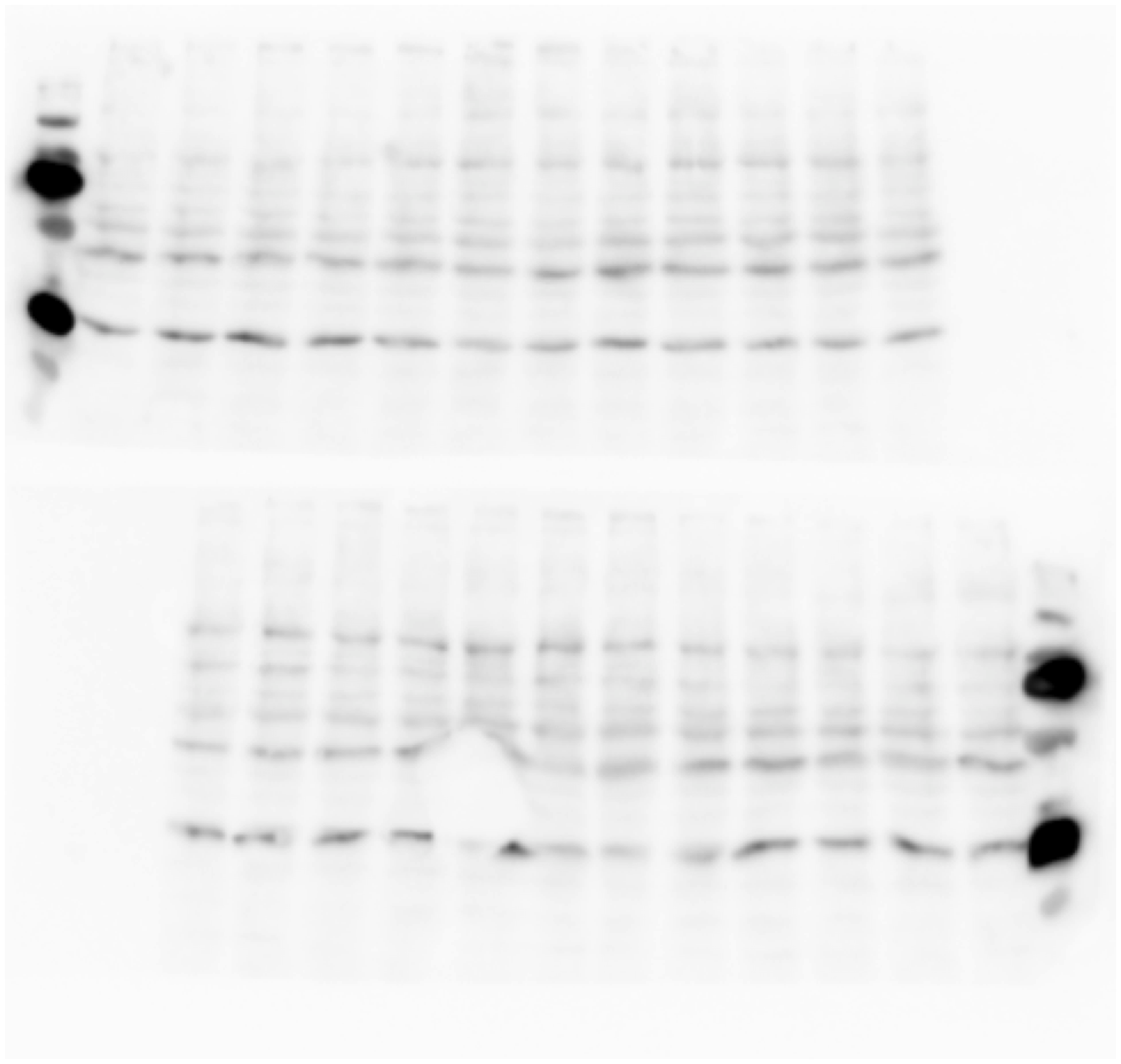

Supplement: Figure 5—figure supplement 1—source data 2. [file elife-84782-fig5-figsupp1-data2.zip › Figure S7_CYP8B1.tiff]

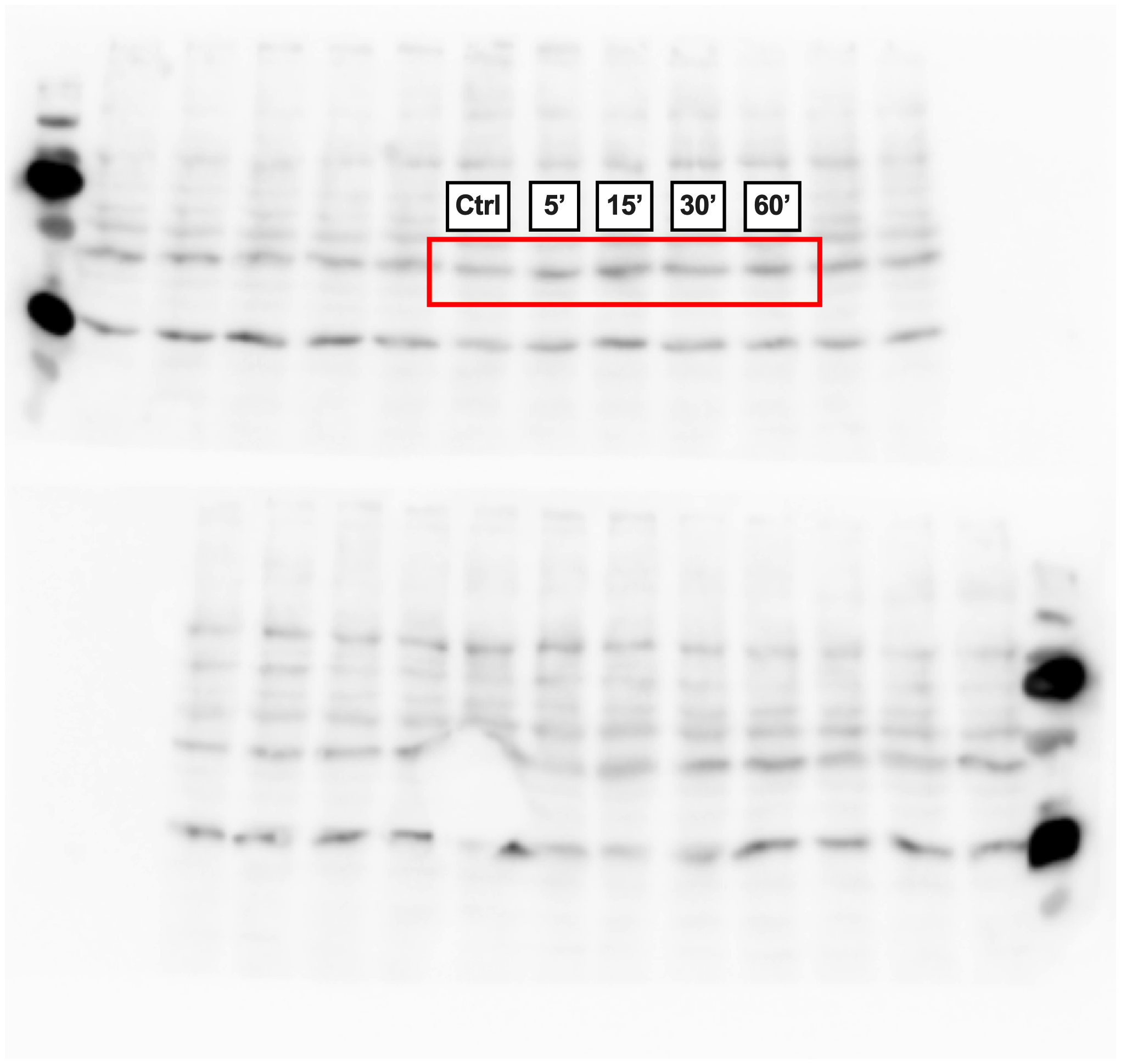

Supplement: Figure 5—figure supplement 1—source data 2. [file elife-84782-fig5-figsupp1-data2.zip › Figure S7_CYP8B1_labeled.tiff]

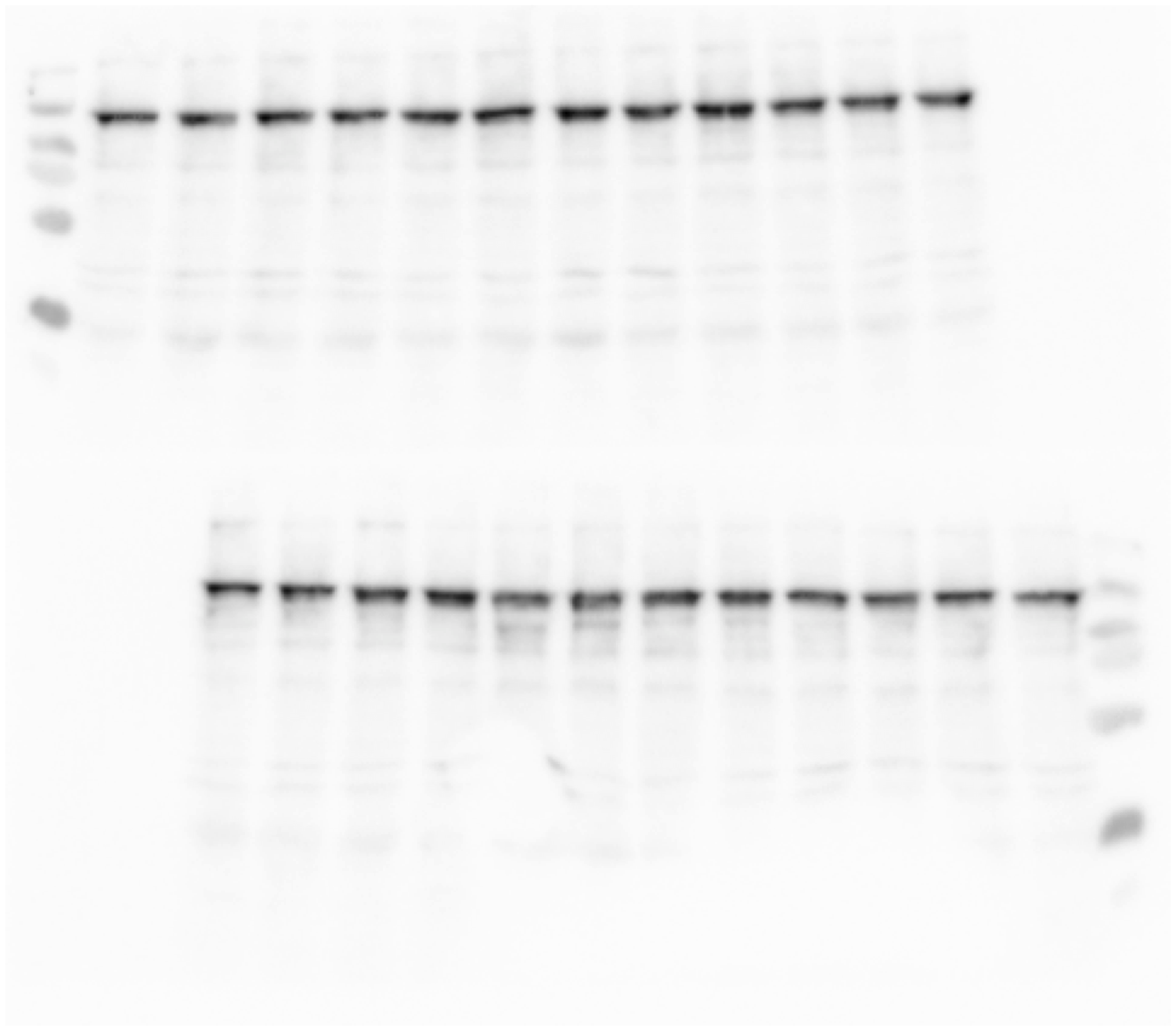

Supplement: Figure 5—figure supplement 1—source data 2. [file elife-84782-fig5-figsupp1-data2.zip › Figure S7_vinculin.tiff]

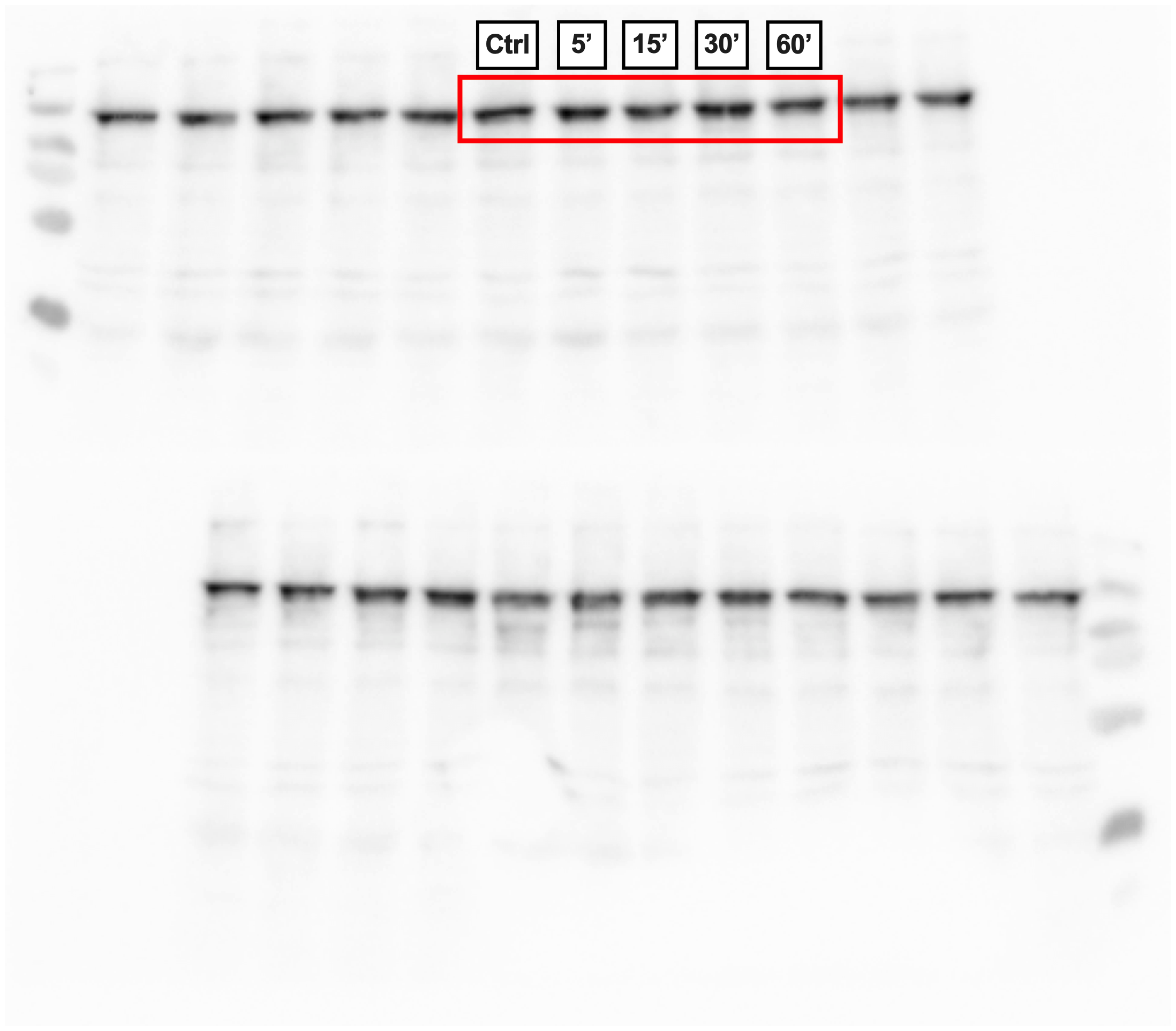

Supplement: Figure 5—figure supplement 1—source data 2. [file elife-84782-fig5-figsupp1-data2.zip › Figure S7_vinculin_labeled.tiff]

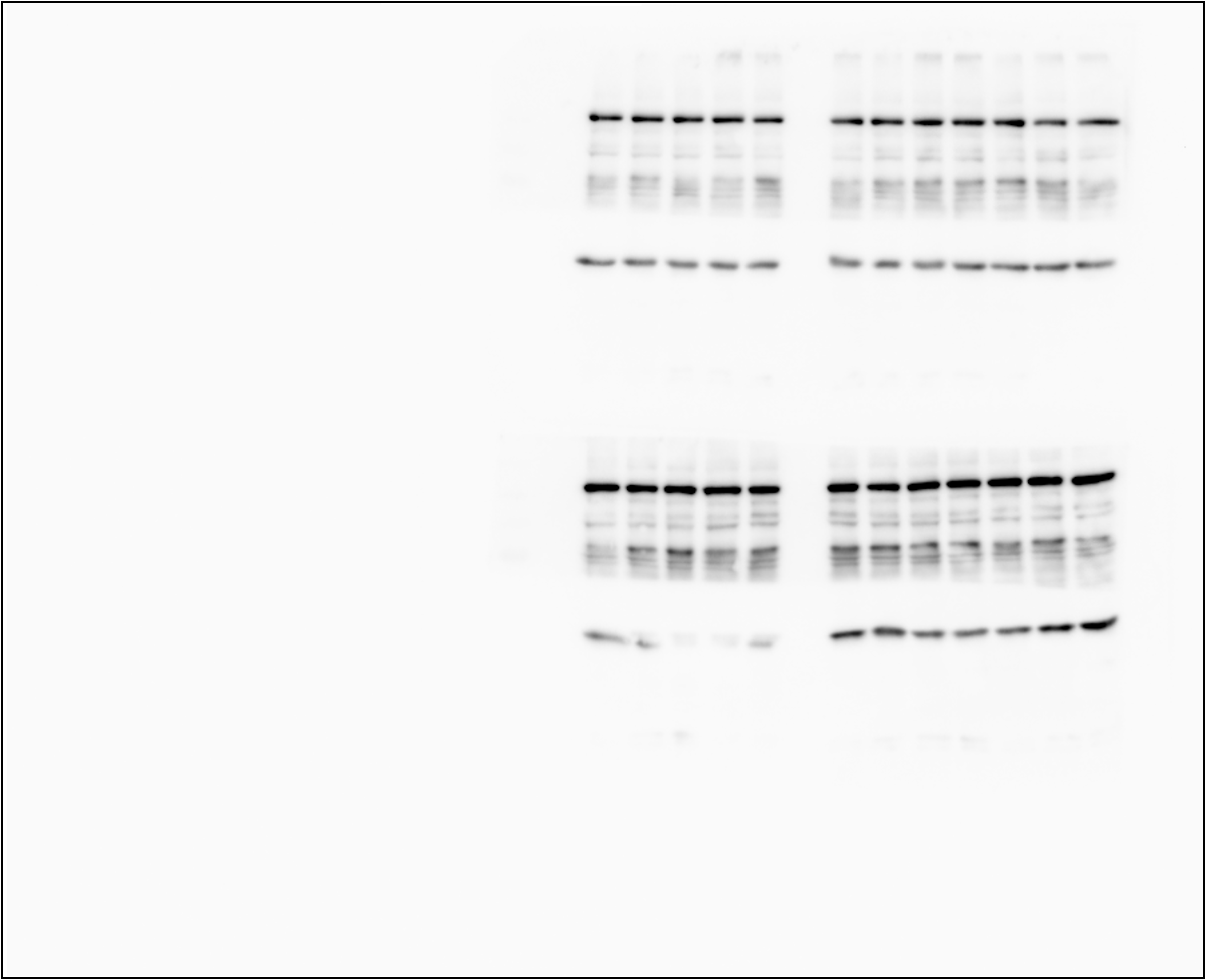

Supplement: Figure 6—source data 1. [file elife-84782-fig6-data1.zip › Figure 6E LDLR_B-actin.tiff]

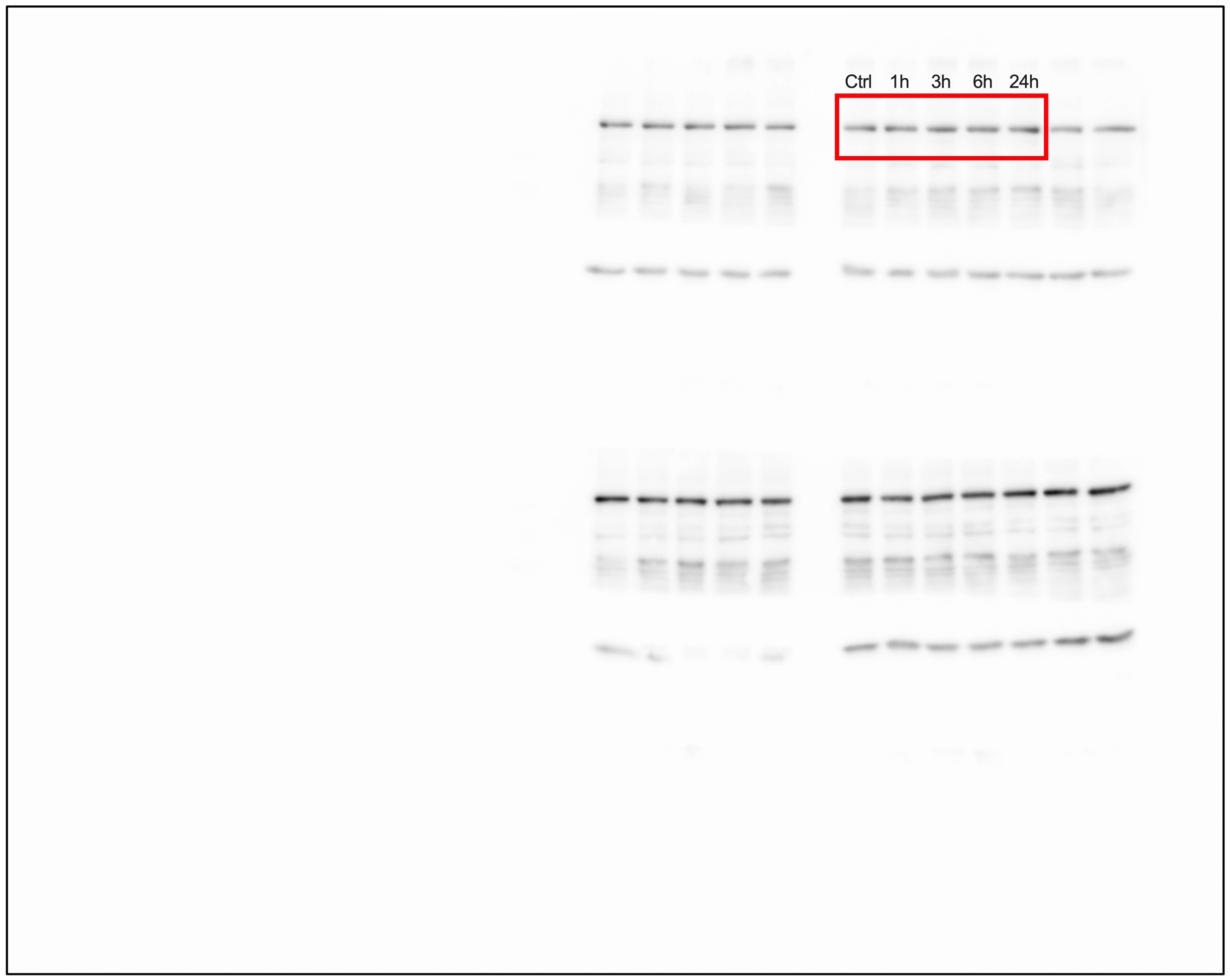

Supplement: Figure 6—source data 1. [file elife-84782-fig6-data1.zip › Figure 6 E LDLR labelled.tiff]

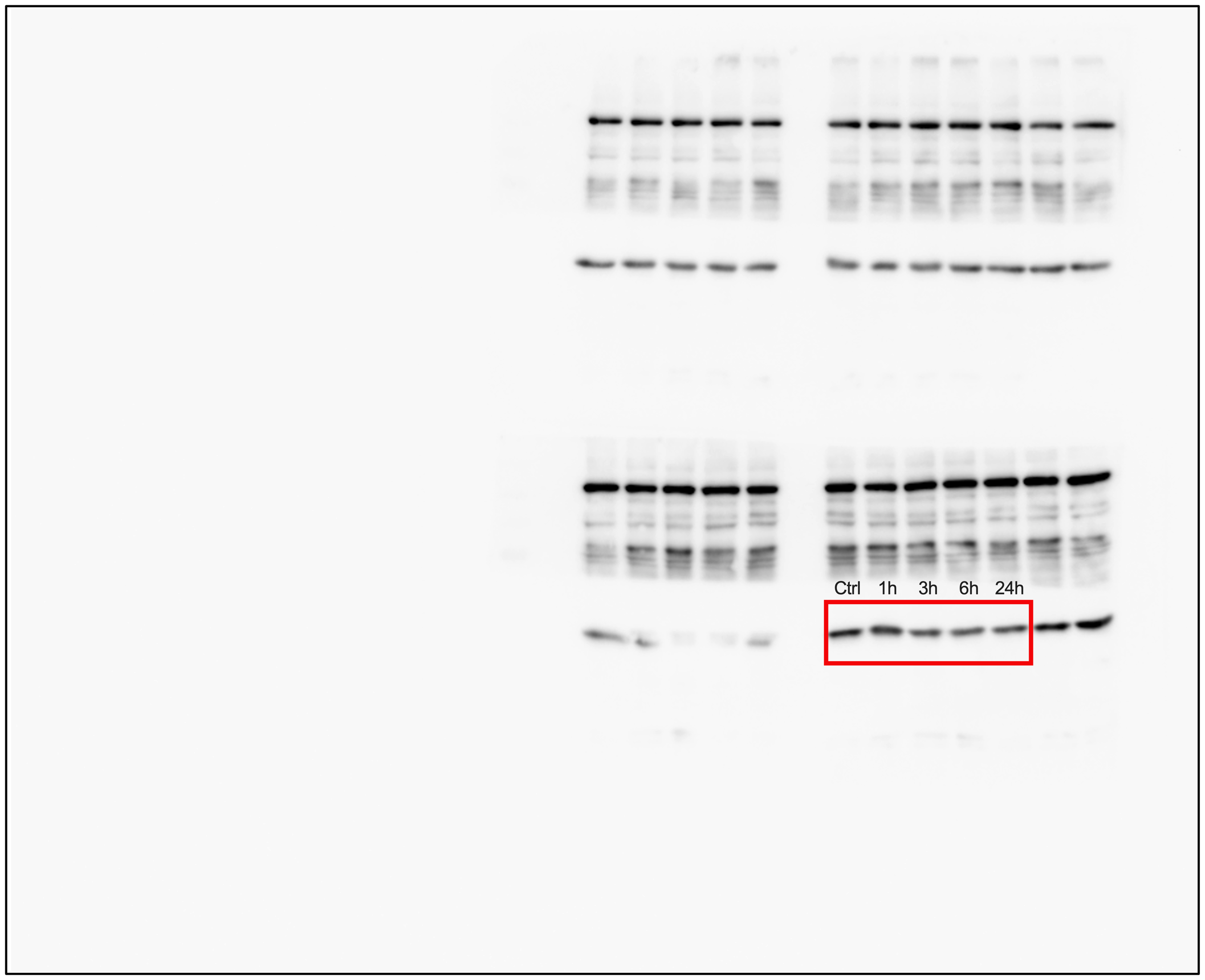

Supplement: Figure 6—source data 1. [file elife-84782-fig6-data1.zip › Figure 6 E SRBI_B-actin labelled.tiff]

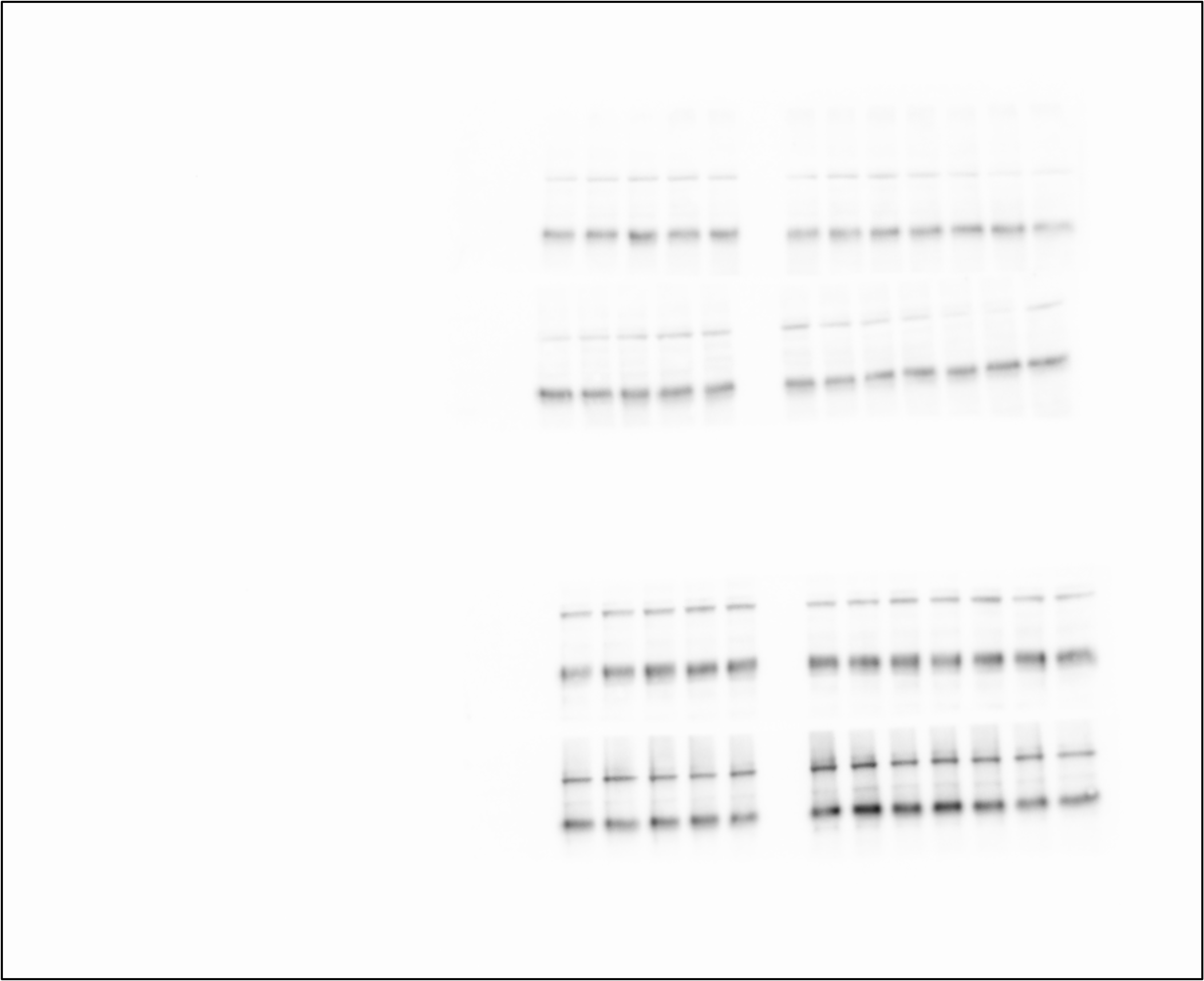

Supplement: Figure 6—source data 1. [file elife-84782-fig6-data1.zip › Figure 6E SR-BI.tiff]

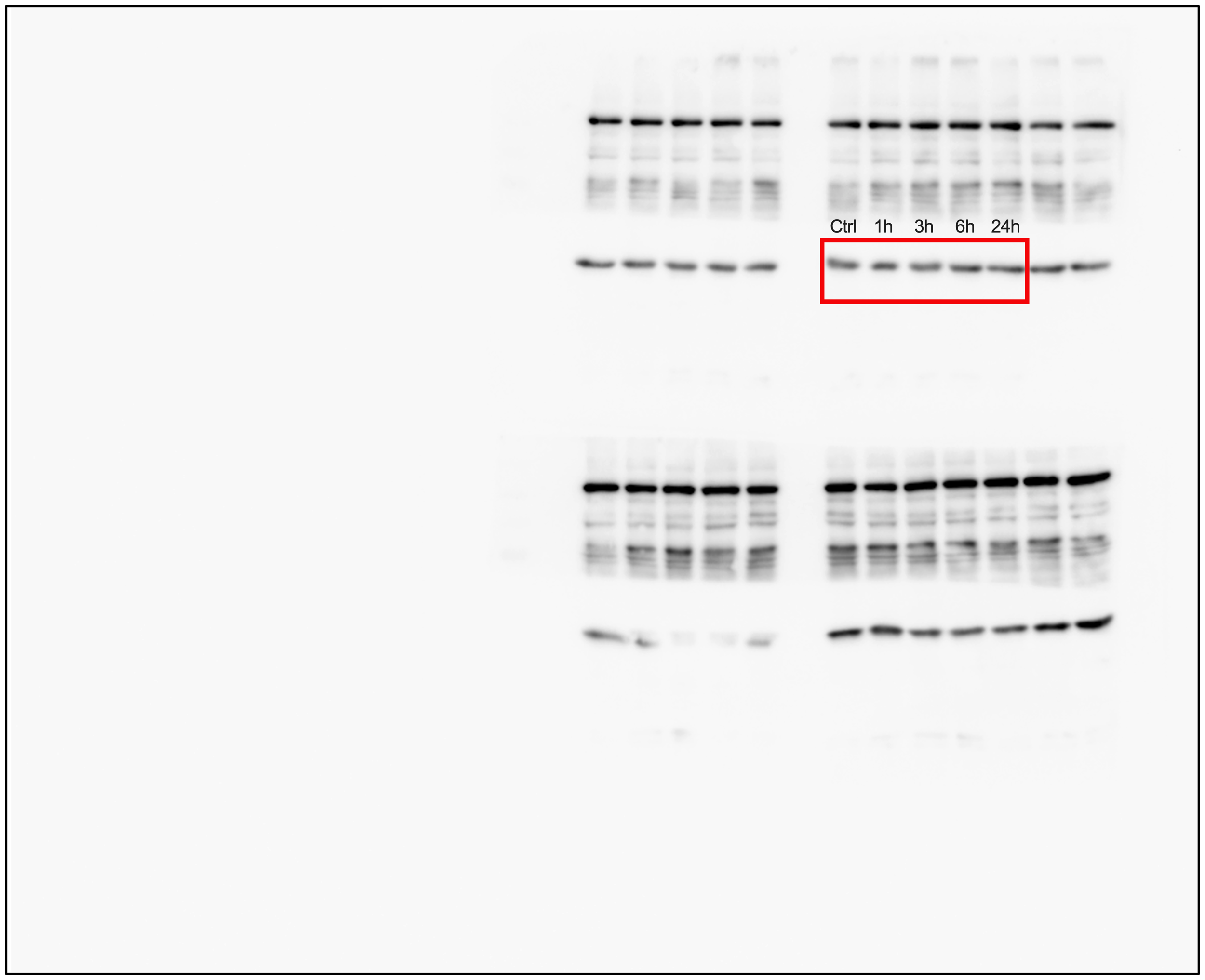

Supplement: Figure 6—source data 1. [file elife-84782-fig6-data1.zip › Figure 6 E LDLR_B-actin labelled.tiff]

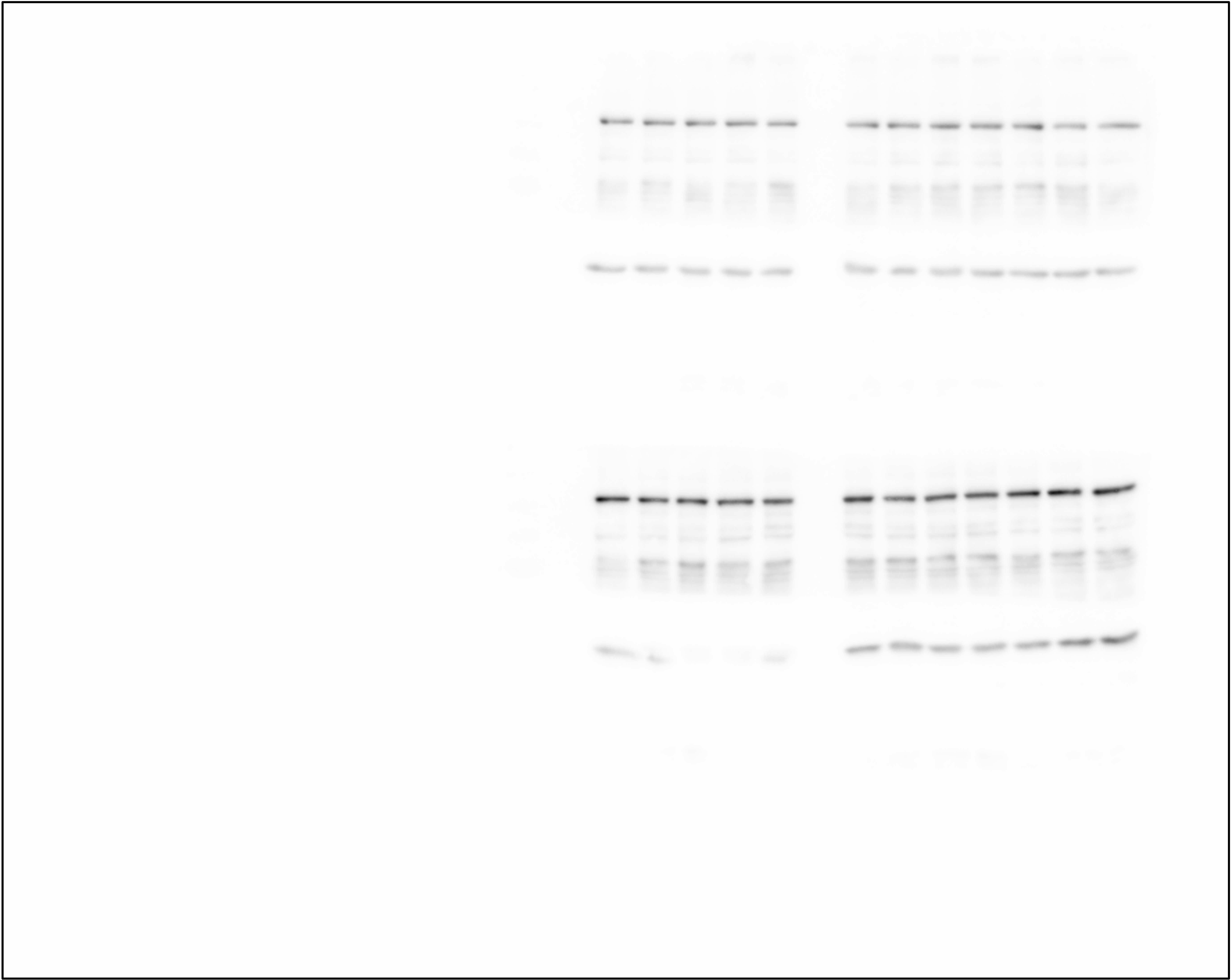

Supplement: Figure 6—source data 1. [file elife-84782-fig6-data1.zip › Figure 6E LDLR.tiff]

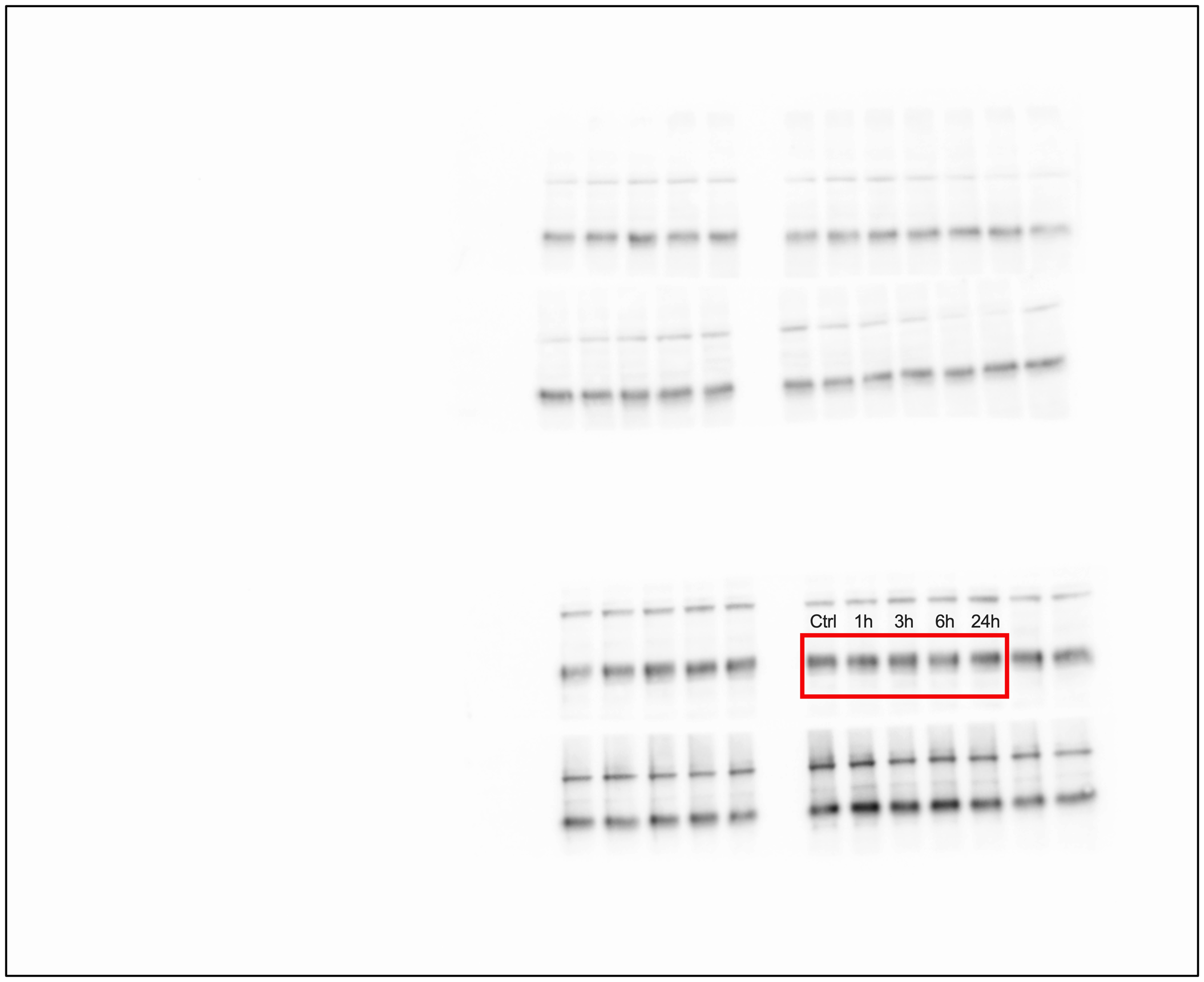

Supplement: Figure 6—source data 1. [file elife-84782-fig6-data1.zip › Figure 6 E SRBI labelled.tiff]

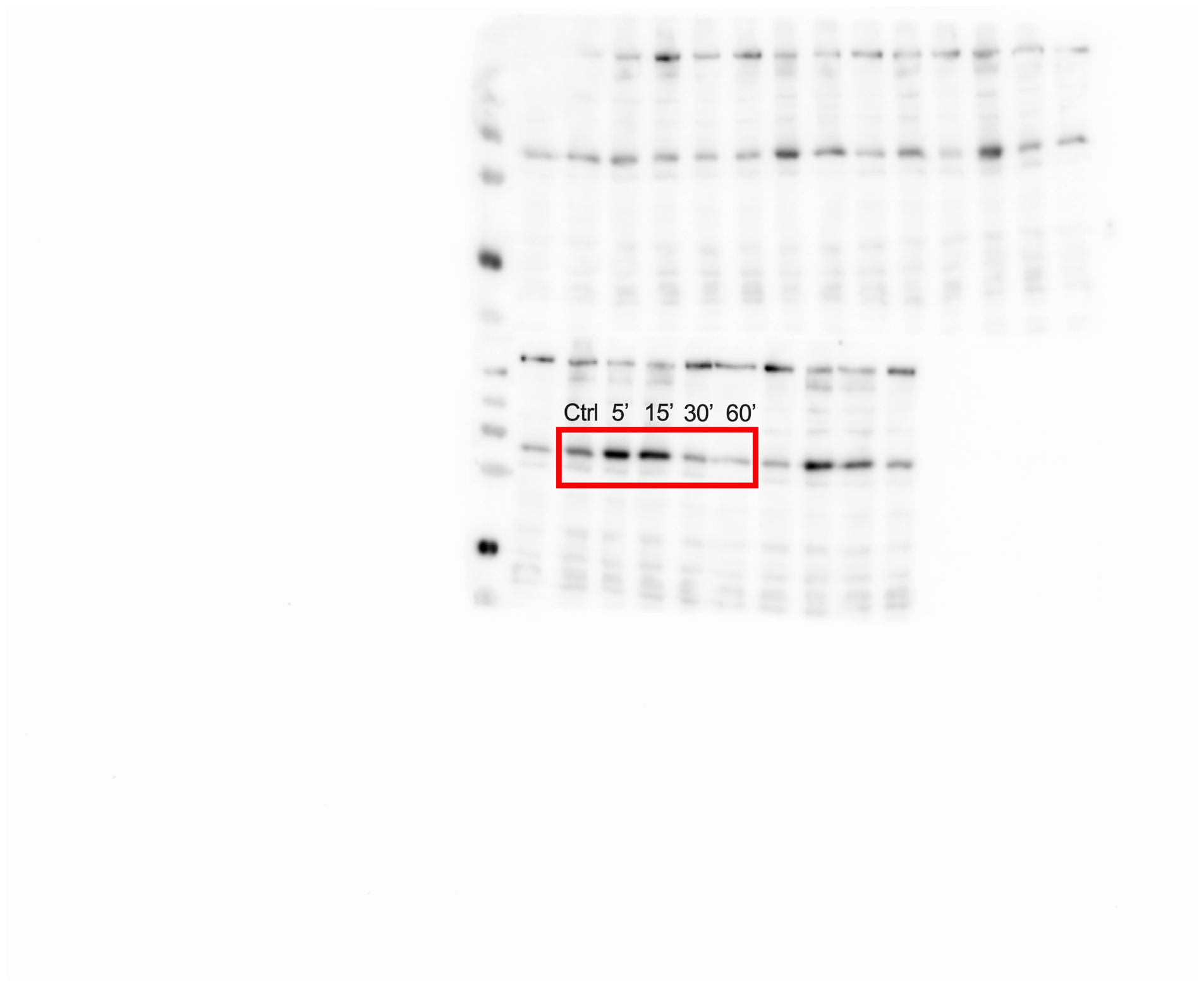

Supplement: Figure 7—source data 1. [file elife-84782-fig7-data1.zip › Figure 7D_p-AMPK_labelled.tiff]

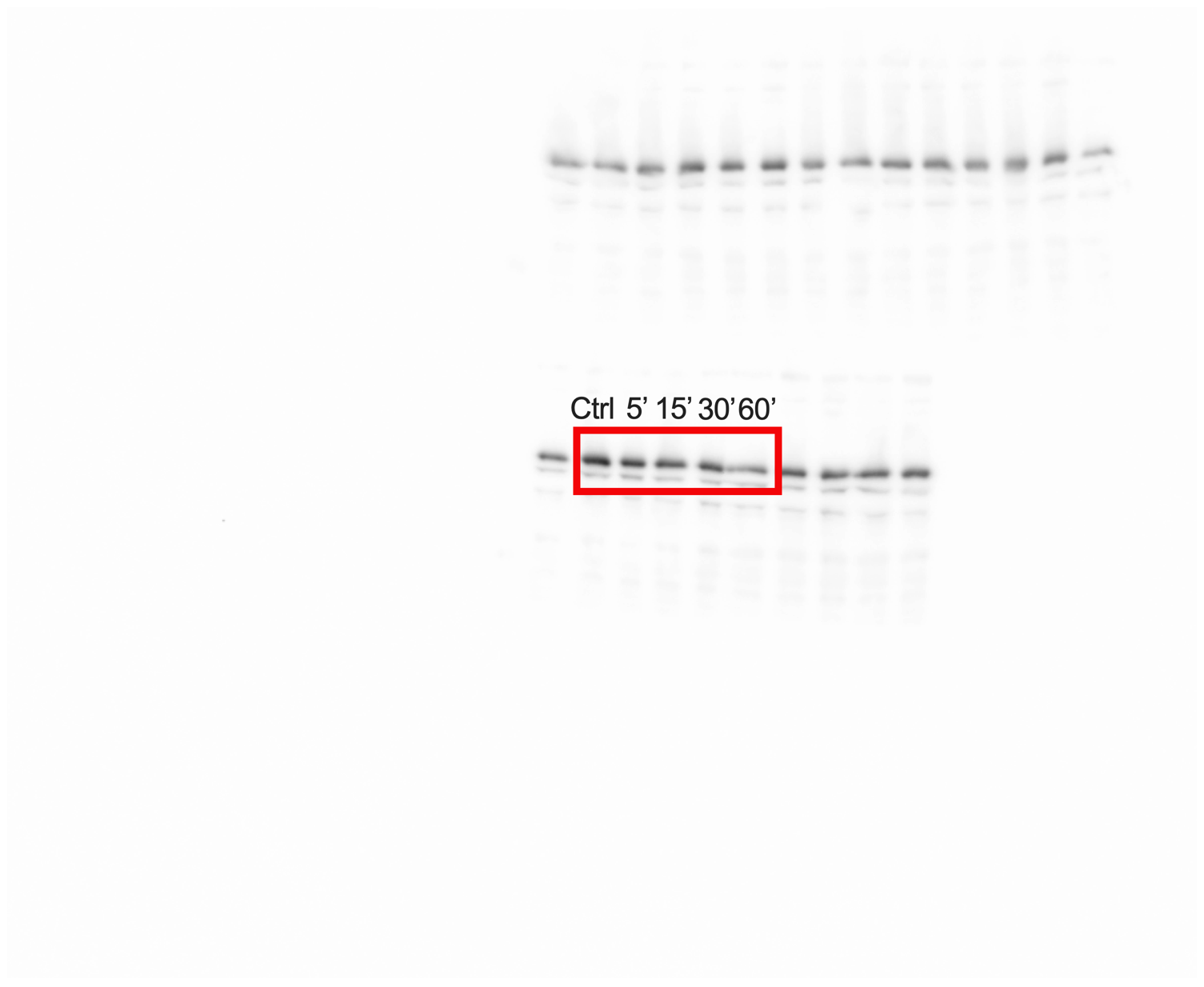

Supplement: Figure 7—source data 1. [file elife-84782-fig7-data1.zip › Figure 7D Total AMPK_labeled.tiff]

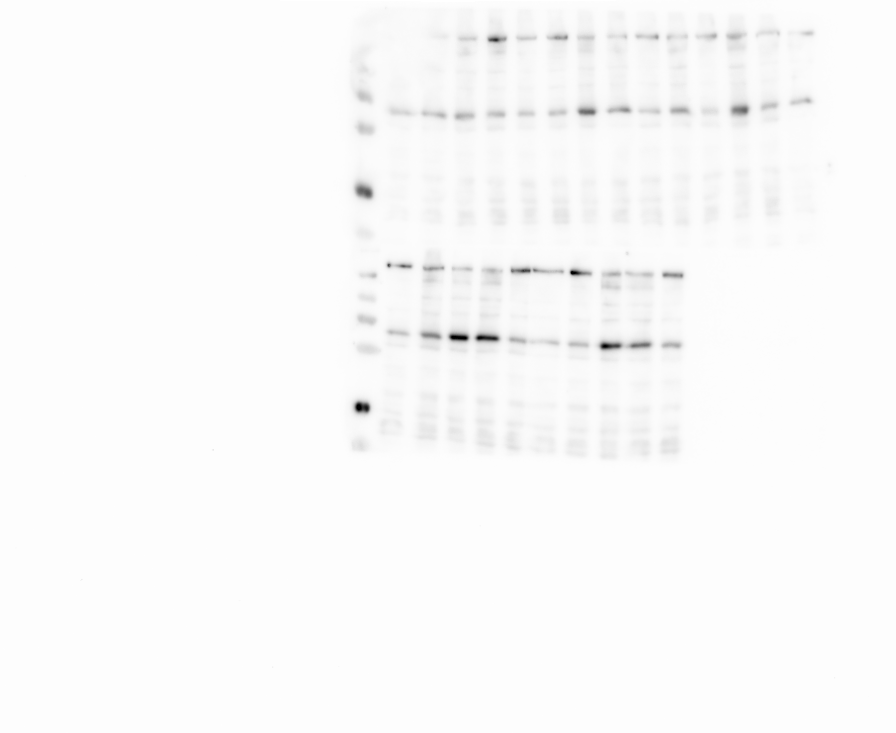

Supplement: Figure 7—source data 1. [file elife-84782-fig7-data1.zip › Figure 7D_p-AMPK.tif]

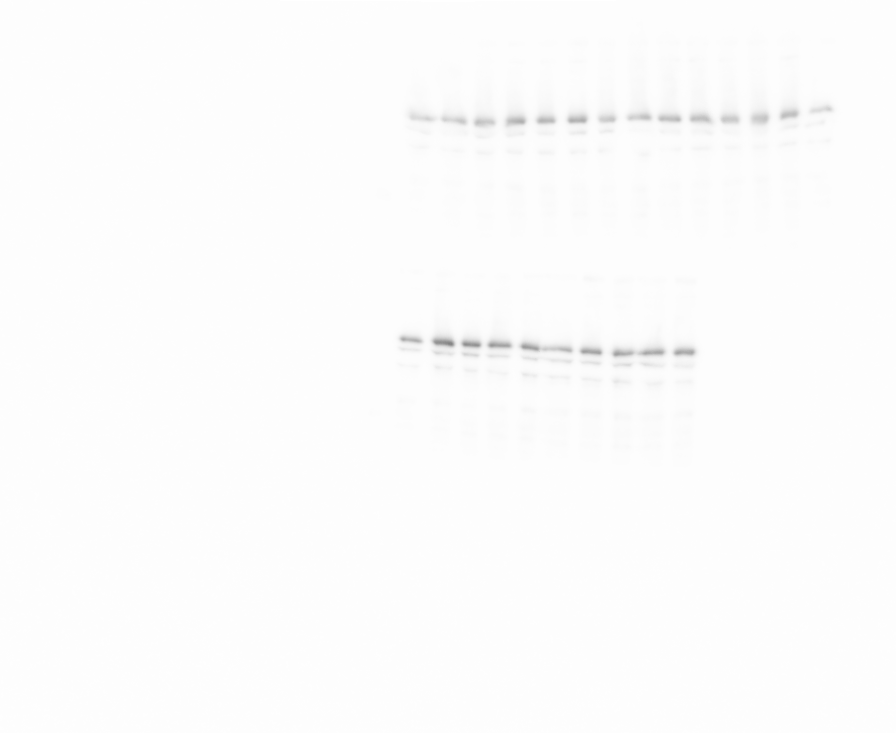

Supplement: Figure 7—source data 1. [file elife-84782-fig7-data1.zip › Figure 7D_Total_AMPK.tif]

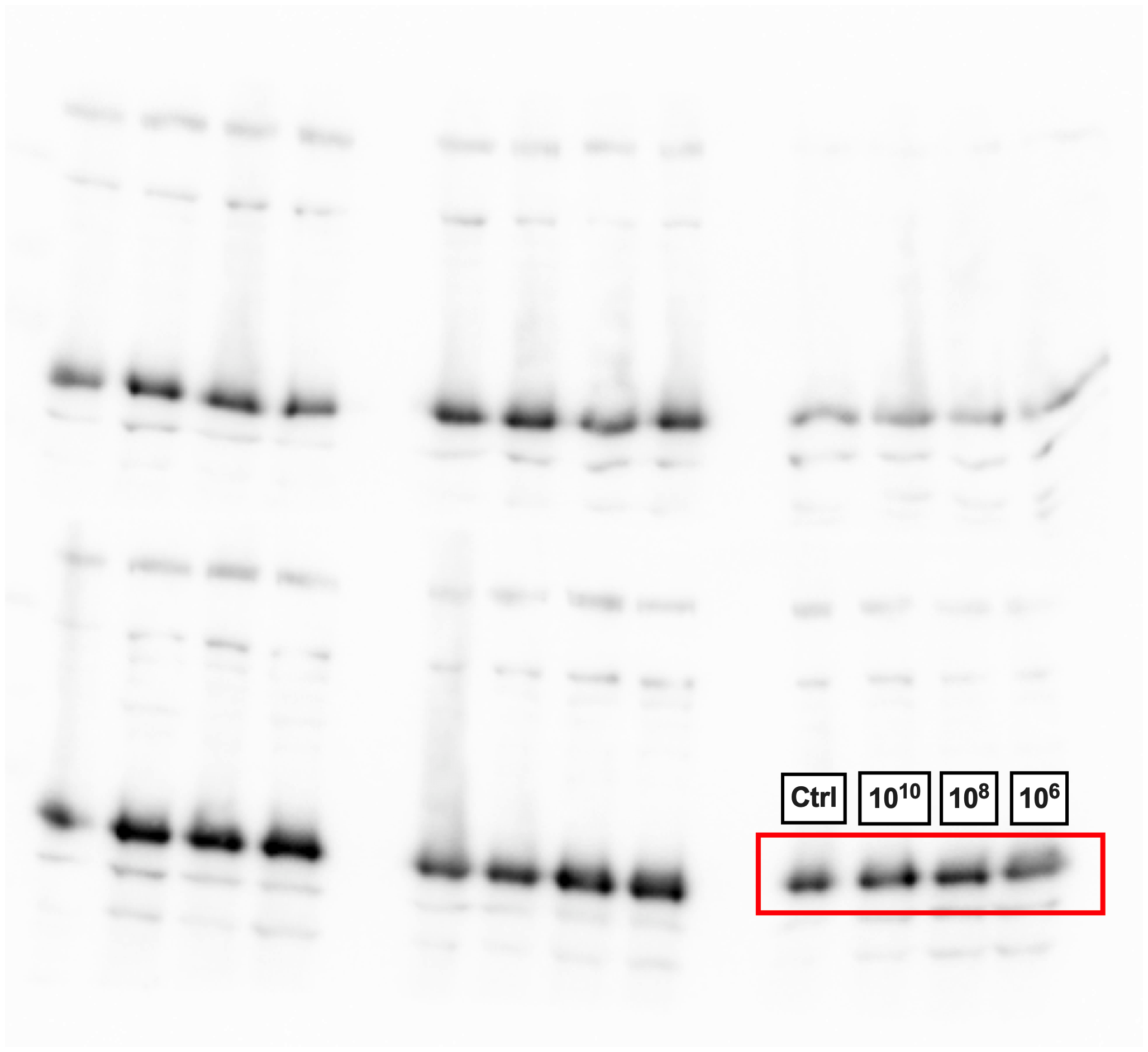

Supplement: Figure 7—figure supplement 1—source data 1. [file elife-84782-fig7-figsupp1-data1.zip › Figure S9C_total AMPK_labelled.tiff]

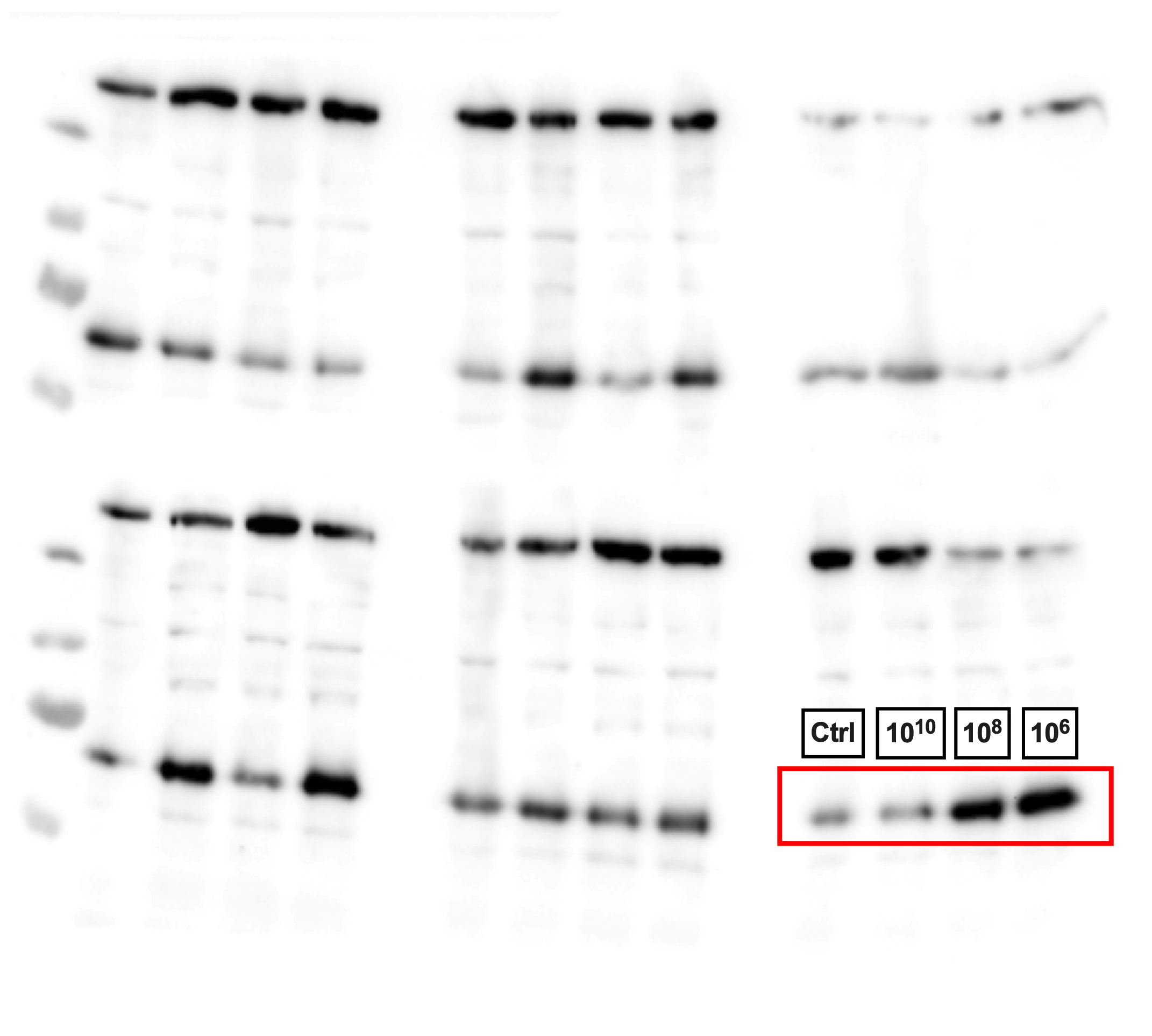

Supplement: Figure 7—figure supplement 1—source data 1. [file elife-84782-fig7-figsupp1-data1.zip › Figure S9C_p-AMPK_labelled.tiff]

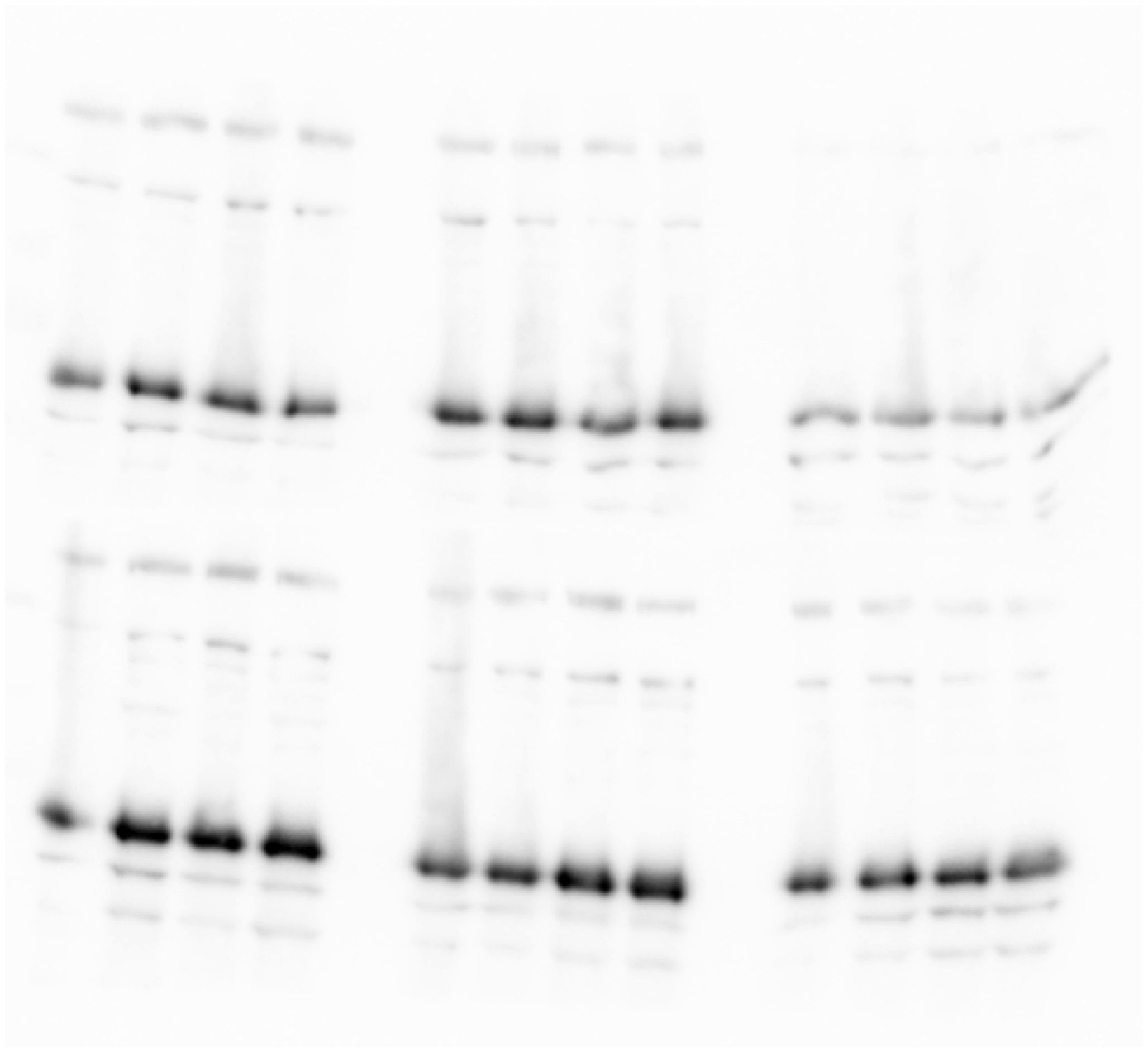

Supplement: Figure 7—figure supplement 1—source data 1. [file elife-84782-fig7-figsupp1-data1.zip › Figure S9C_total AMPK.tiff]

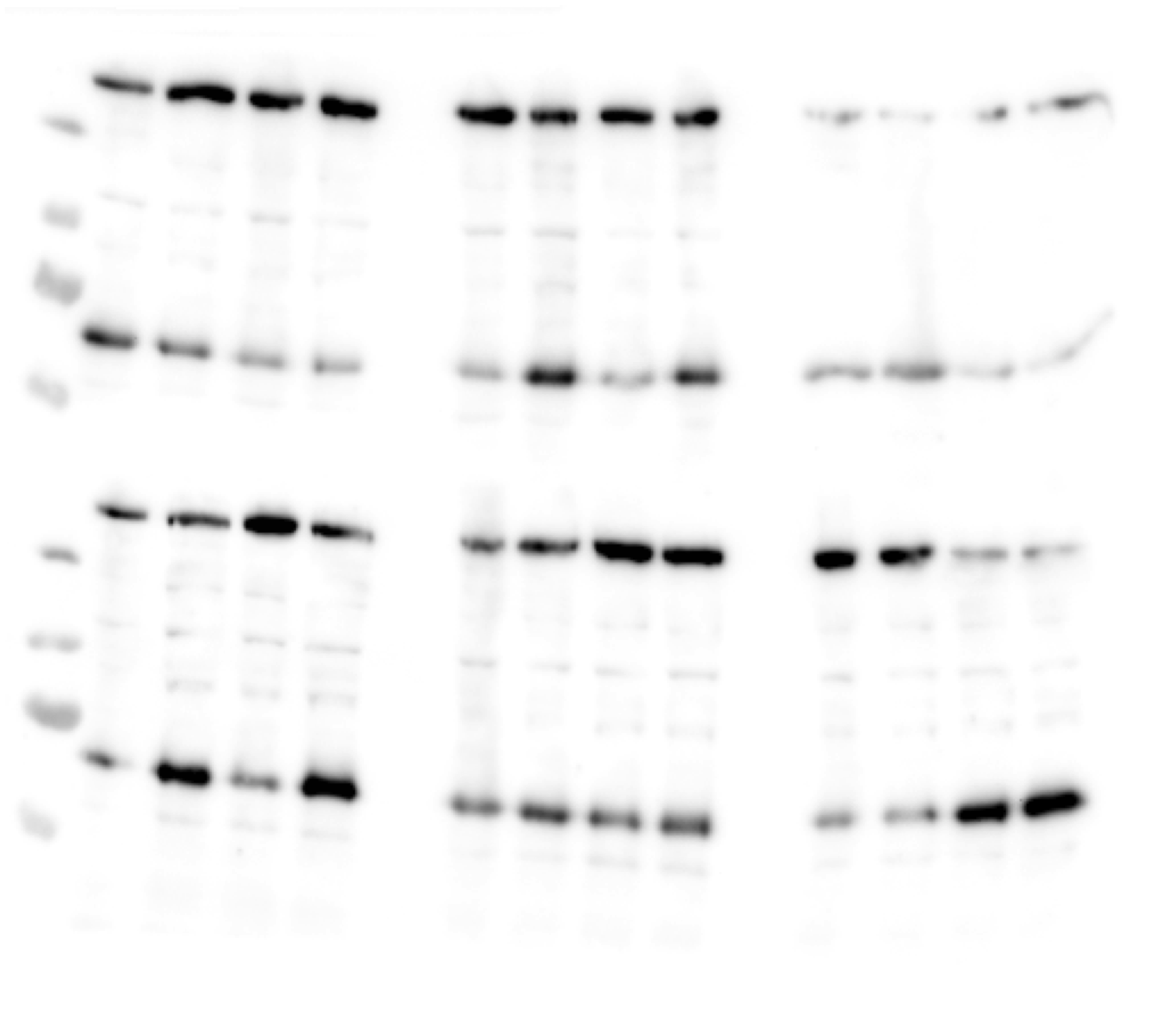

Supplement: Figure 7—figure supplement 1—source data 1. [file elife-84782-fig7-figsupp1-data1.zip › Figure S9C_p-AMPK.tiff]

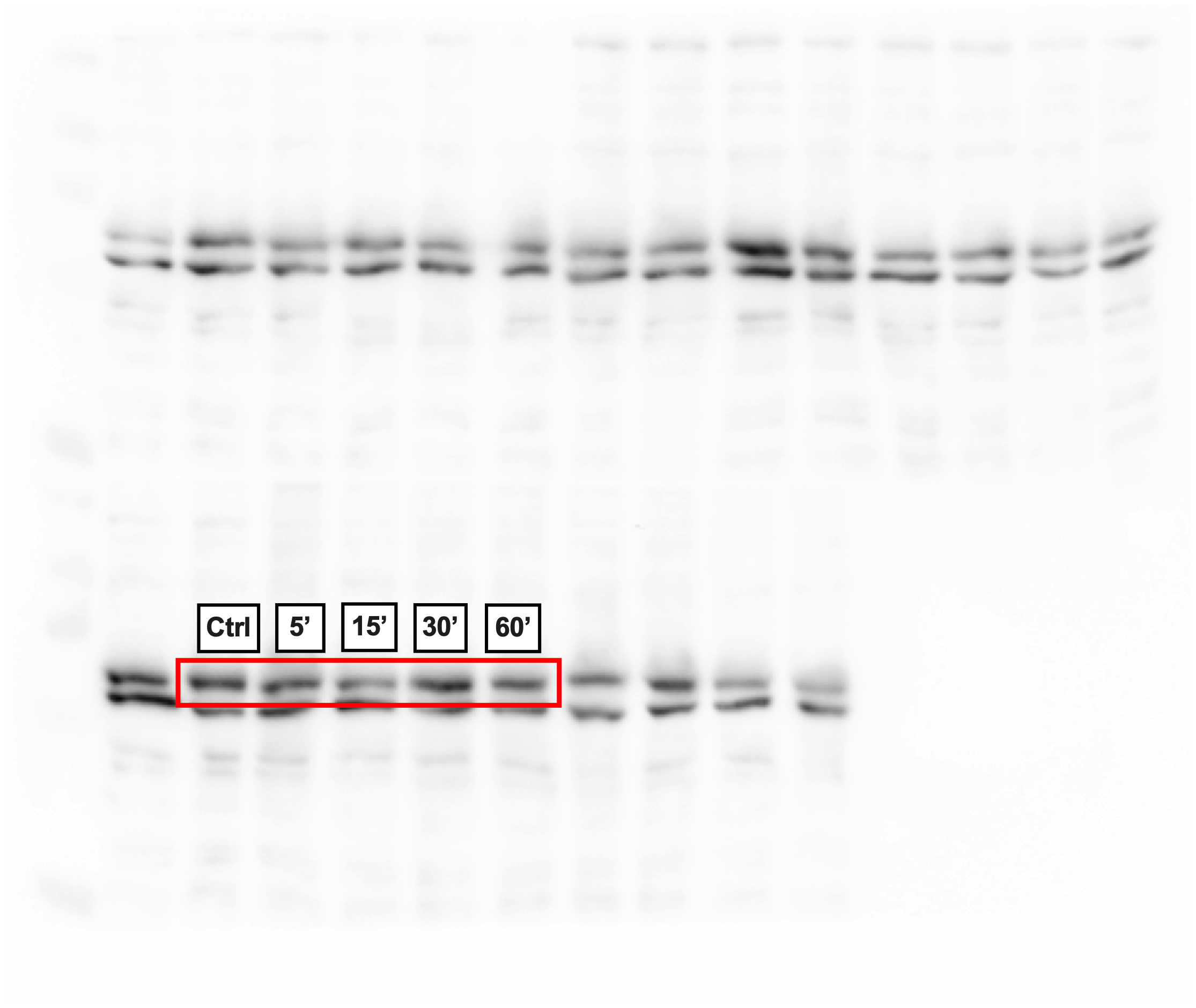

Supplement: Figure 7—figure supplement 2—source data 1. [file elife-84782-fig7-figsupp2-data1.zip › Figure S10D_total AMPK_labeled.tiff]

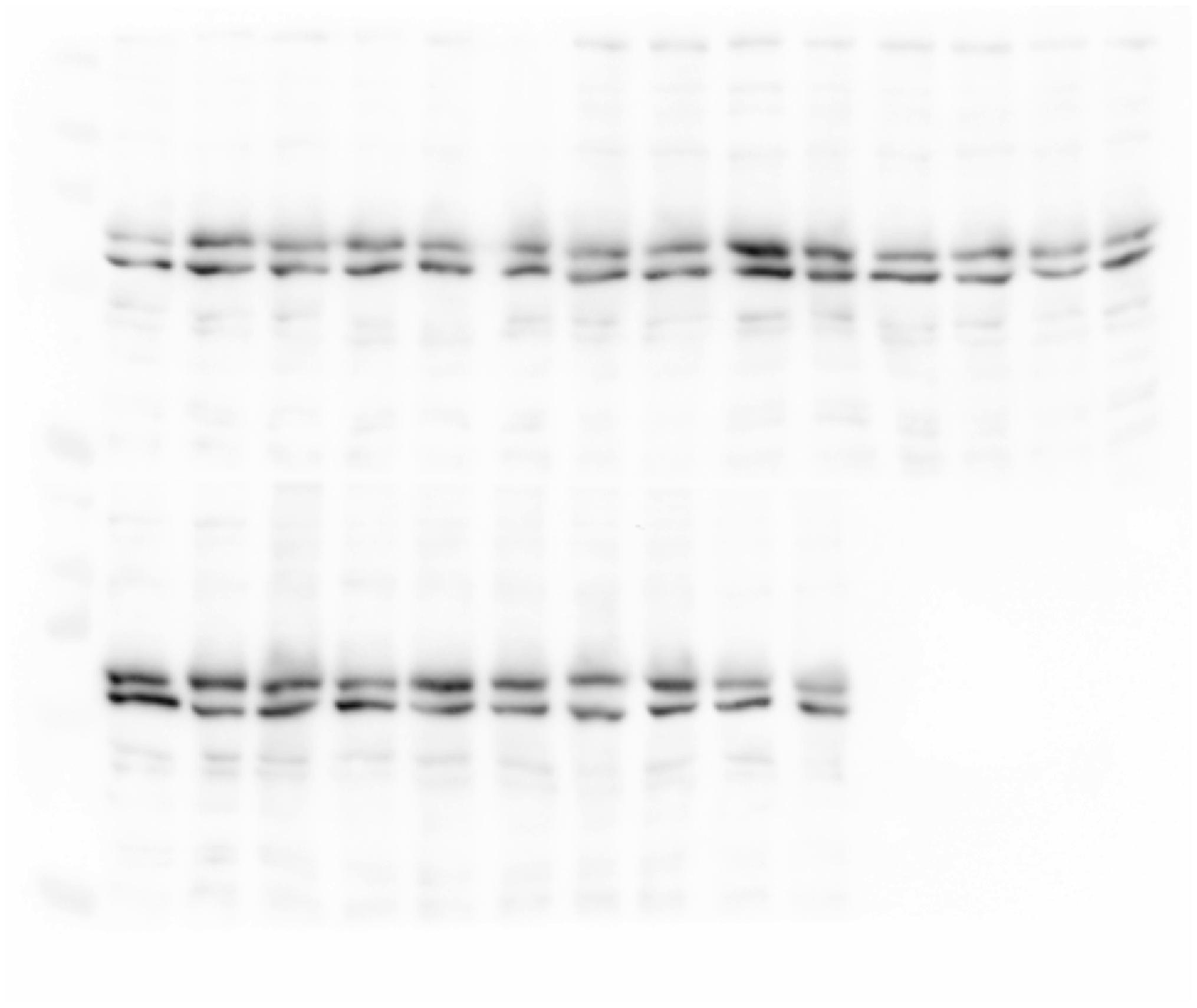

Supplement: Figure 7—figure supplement 2—source data 1. [file elife-84782-fig7-figsupp2-data1.zip › Figure S10D_total AMPK.tiff]

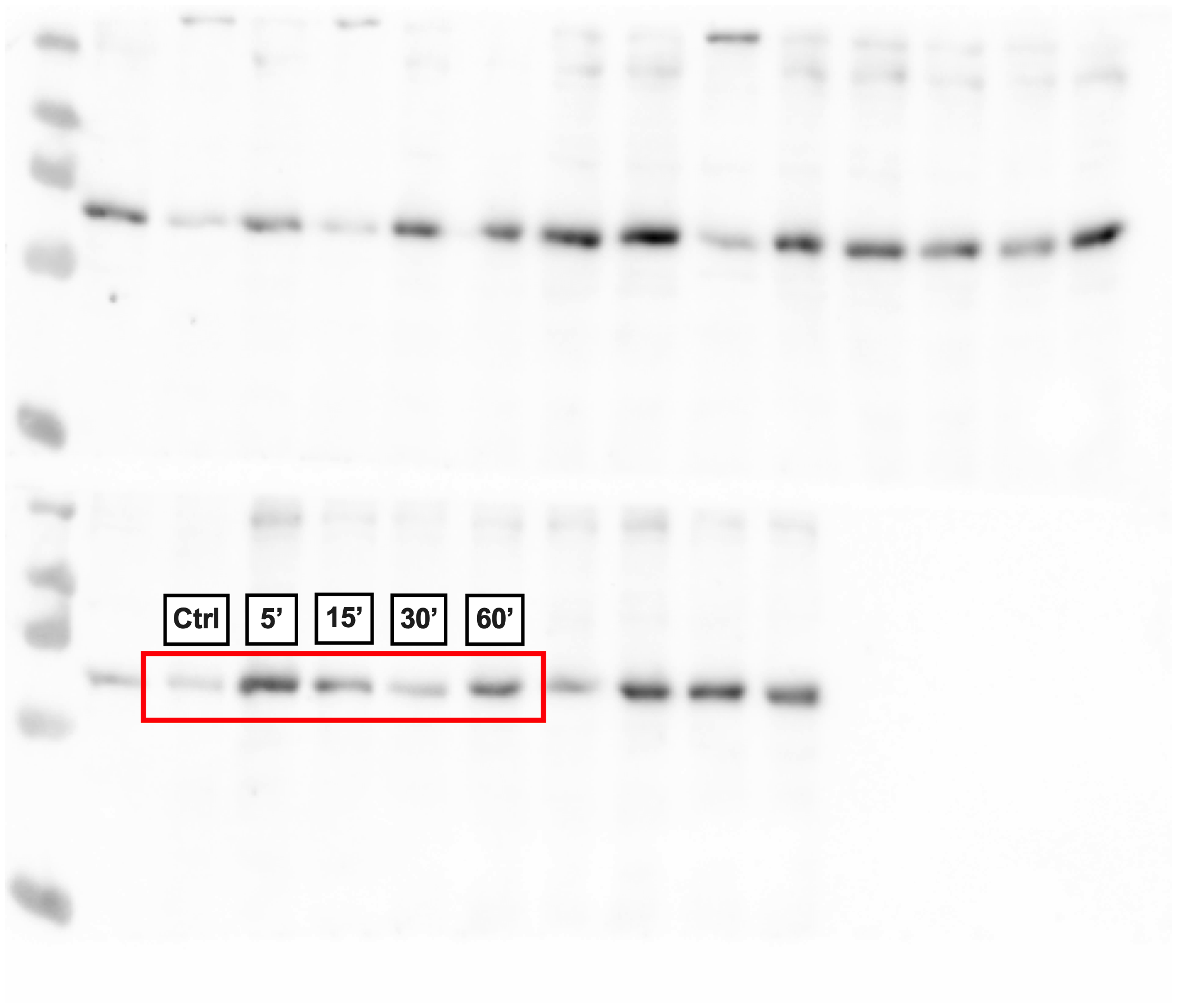

Supplement: Figure 7—figure supplement 2—source data 1. [file elife-84782-fig7-figsupp2-data1.zip › Figure S10D_p-AMPK_labeled.tiff]

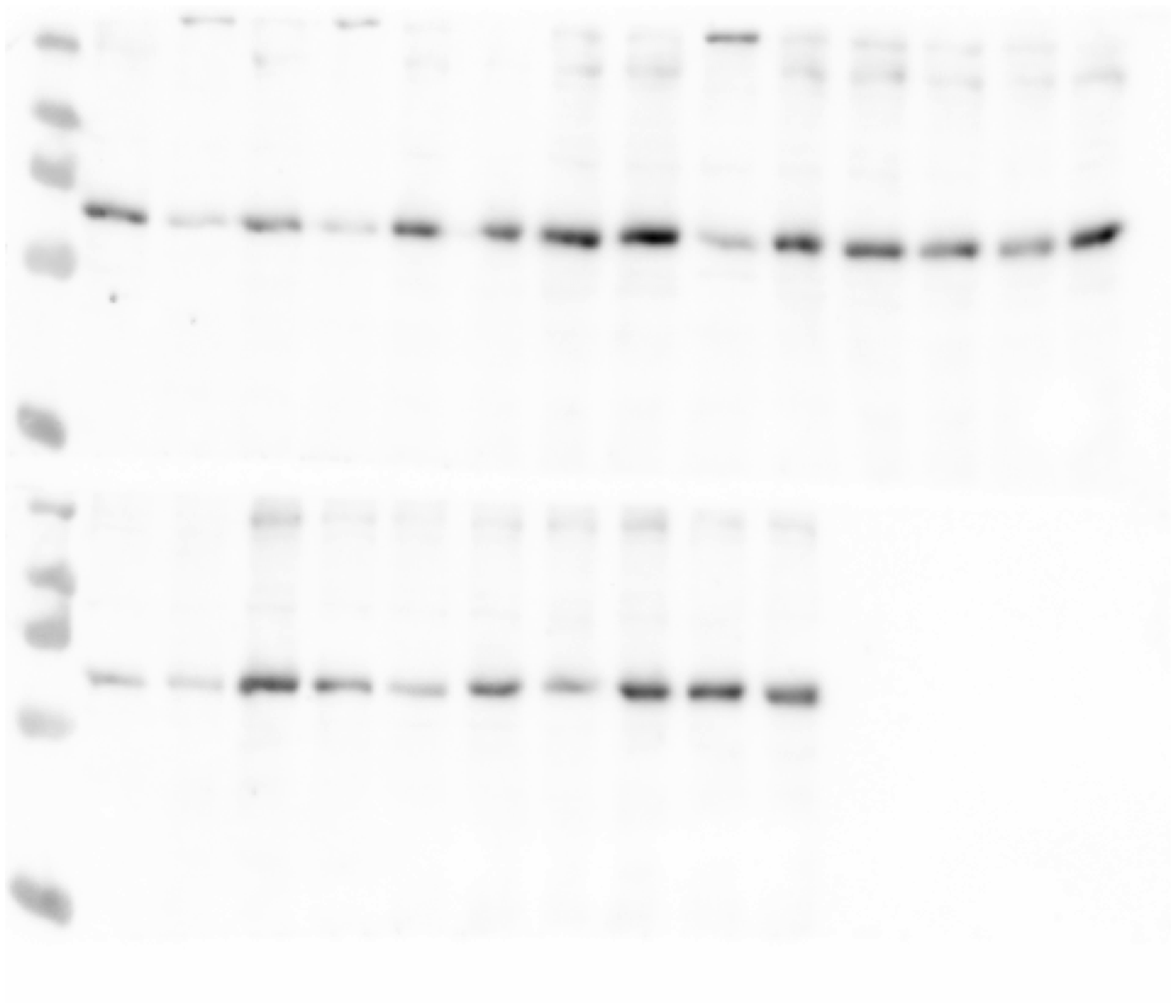

Supplement: Figure 7—figure supplement 2—source data 1. [file elife-84782-fig7-figsupp2-data1.zip › Figure S10D_p-AMPK.tiff]
